# Oxidative Addition of C–Cl Bonds to a Rh(PONOP) Pincer Complex

**DOI:** 10.1021/acs.organomet.2c00400

**Published:** 2022-10-31

**Authors:** Alexandra Longcake, Martin R. Lees, Mark S. Senn, Adrian B. Chaplin

**Affiliations:** † Department of Chemistry, University of Warwick, Gibbet Hill Road, CoventryCV4 7AL, U.K.; ‡ Department of Physics, University of Warwick, Gibbet Hill Road, CoventryCV4 7AL, U.K.

## Abstract

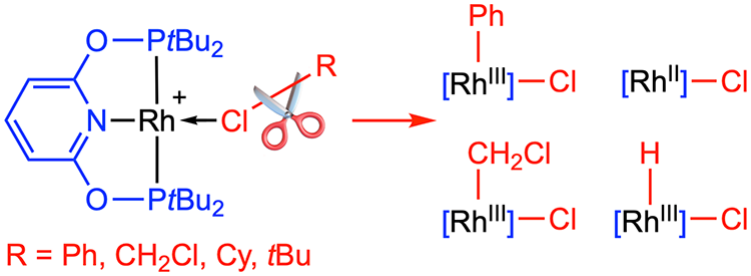

Straightforward procedures for the generation of rhodium(I) κ_Cl_–chlorocarbon complexes of the form [Rh(PONOP-*t*Bu)(*κ*_Cl_–ClR)][BAr^F^_4_] [R = CH_2_Cl, **A**; Ph, **1**; Cy, **2**; *t*Bu, **3**; PONOP-*t*Bu = 2,6-bis(di-*tert*-butylphosphinito)pyridine; Ar^F^ = 3,5-bis(trifluoromethyl)phenyl] in solution are described, enabling isolation of analytically pure **A** and crystallographic characterization of the new complexes **1** and **2**. Complex **1** was found to be stable at ambient temperature, but prolonged heating in chlorobenzene at 125 °C resulted in formation of [Rh(PONOP-*t*Bu)(Ph)Cl][BAr^F^_4_] **4** with experimental and literature evidence pointing toward a concerted C(sp^2^)–Cl bond oxidative addition mechanism. C(sp^3^)–Cl bond activation of dichloromethane, chlorocyclohexane, and 2-chloro-2-methylpropane by the rhodium(I) pincer occurred under considerably milder conditions, and radical mechanisms that commence with chloride atom abstraction and involve generation of the rhodium(II) metalloradical [Rh(PONOP-*t*Bu)Cl][BAr^F^_4_] **6** are instead proposed. For dichloromethane, [Rh(PONOP-*t*Bu)(CH_2_Cl)Cl][BAr^F^_4_] **5** was formed in the dark, but facile photo-induced reductive elimination occurred when exposed to light. Net dehydrochlorination affording [Rh(PONOP-*t*Bu)(H)Cl][BAr^F^_4_] **7** and an alkene byproduct resulted for chlorocyclohexane and 2-chloro-2-methylpropane, consistent with hydrogen atom abstraction from the corresponding alkyl radicals by **6**. This suggestion is supported by dynamic hydrogen atom transfer between **6** and **7** on the ^1^H NMR time scale at 298 K in the presence of TEMPO.

## 1 Introduction

**Scheme 1:**
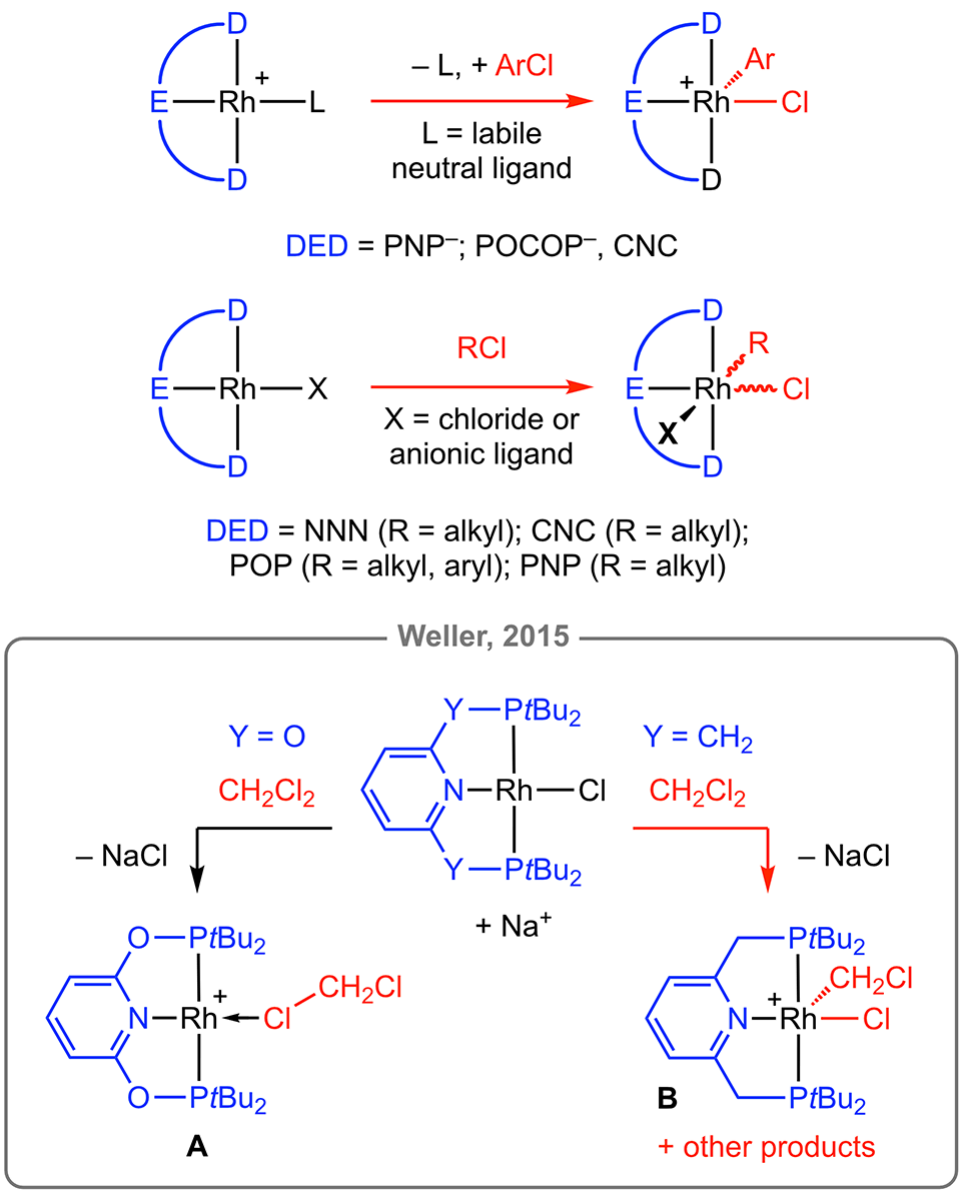
Oxidative Addition of C–Cl Bonds to Rhodium(I) Pincer Complexes

The activation of organohalides by C–X bond oxidative addition to late transition metal complexes is a keystone organometallic transformation with diverse applications in catalysis.^1^ Despite economic and environmental imperatives for the use of chlorocarbons as substrates, the robust nature of C–Cl bonds remains a significant practical impediment, conferring attenuated or divergent reactivity compared to heavier halide counterparts.^1,2^ With respect to well-defined rhodium complexes, only a limited number of examples of C–Cl bond activation can be found in the literature, but the use of rigid *mer*-tridentate “pincer” ligands is an emerging trend (Scheme 1).^3−12^ These versatile ancillary ligands are evidently well-suited to supporting the reactive rhodium centers required to bring about cleavage of a C–Cl bond.^13^

The activation of aryl chlorides by rhodium(I) pincers is of particular interest for applications in catalysis^14^ and typically associated with transient three-coordinate rhodium(I) derivatives, for which concerted oxidative addition mechanisms that proceed with high selectivity over C–H bond activation have been substantiated by computational studies.^3,4^ A wider range of mechanisms have been proposed for the activation of alkyl chlorides, but classification is obfuscated by more facile entry into nucleophilic and radical oxidative addition manifolds. Indeed, most documented examples are based on reactions of square planar rhodium(I) chloride complexes (X = Cl in Scheme 1), where the stereochemistry of the oxidative addition can be masked in the product.^5,6^ As part of their work with rhodium(I) xantphos complexes, Esteruelas and co-workers have examined the activation of a range of chlorocarbons by neutral square planar derivatives.^6,7^ In most cases, direct concerted oxidative addition was invoked, including aryl chlorides. Competitive nucleophilic oxidative addition was, however, suggested for dichloromethane to reconcile the formation of *cis*- and *trans*-rhodium(III) dichloride products. This S_N_2 pathway has been proposed for the oxidative addition of dichloromethane to phosphine-based complexes of the form [Rh(PNP)Cl] by comparison to reactions with methyl iodide and studying the effect of the phosphine substituents on the reaction rate (Ph > *i*Pr > *t*Bu > Mes).^8^ Evidence for single-electron reactivity has also emerged for reactions of alkyl chlorides with rhodium(I) pincer complexes. For instance, a cascade of chloride abstraction and single-electron transfer steps is advocated by Hulley and co-workers to account for the formation of the methylidene complex [Rh(POP-*t*Bu)(=CH_2_)]^+^ from the reaction between [Rh(POP-*t*Bu)Cl] and K[B(C_6_F_5_)_4_] in dichloromethane {POP-*t*Bu = 4,6-bis(di-*tert*-butylphosphino)dibenzo[*b*,*d*]furan}.^9^

**Chart 1:**
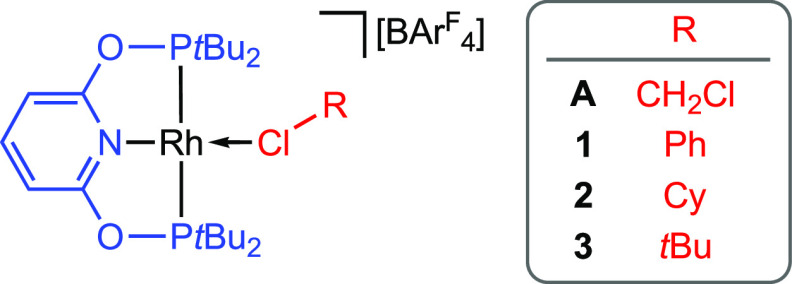
Target rhodium(I) κ_Cl_–chlorocarbon complexes.

Most relevant to the present work, Weller and co-workers have examined reactions of [Rh(PONOP-*t*Bu)Cl] (PONOP-*t*Bu = 2,6-bis(di-*tert*-butylphosphinito)pyridine) and [Rh(PNP-*t*Bu)Cl] (PNP-*t*Bu = 2,6-bis(di-*tert*-butylphosphinomethyl)pyridine) with dichloromethane that are induced by the halide abstracting agent Na[BAr^F^_4_] (Ar^F^ = 3,5-bis(trifluoromethyl)phenyl).^10^ The labile rhodium(I) solvent adduct **A** was obtained in 81% isolated yield from the former, while a mixture of products including rhodium(III) complex **B** were generated from the latter. Late transition metal κ_Cl_–chlorocarbon complexes such as **A** are rare, with dichloroethane complexes [Rh(R_2_PCH_2_PR_2_)(*κ*_Cl,Cl_–ClCH_2_CH_2_Cl)][BAr^F^_4_] (R = *i*Pr, *i*Bu) the only other crystallographically characterized rhodium(I) examples deposited in the Cambridge Structural Database (CSD v.5.43, update June 2022).^11,15,16^ Intrigued by the prospect of studying their onward reactivity, especially in connection to C–Cl bond activation, we targeted isolation of new rhodium(I) κ_Cl_–chlorocarbon complexes of the form [Rh(PONOP-*t*Bu)(*κ*_Cl_–ClR)][BAr^F^_4_] (R = Ph, **1**; Cy, **2**; *t*Bu, **3**; Chart 1). We herein report upon our efforts to this end, along with re-examining the synthesis and reactivity of **A**.

## 2 Results and Discussion

**Figure 1:**
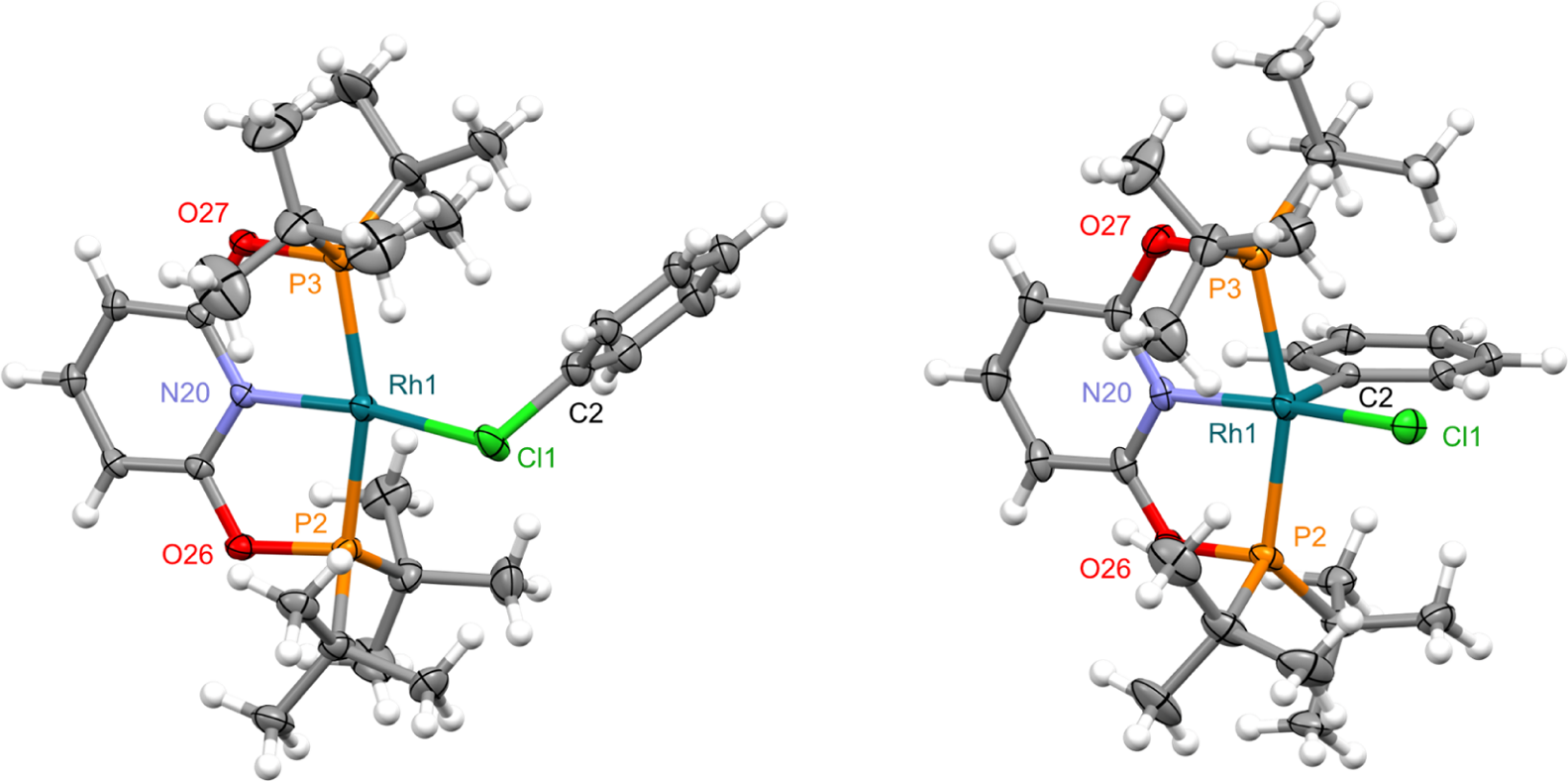
Solid-state structures of **1** (left) and **4** (right) with thermal ellipsoids at 30% probability. Minor disordered components (Ph in **1**) and anions omitted. Selected bond lengths (Å) and angles (°): **1**, Rh1–Cl1, 2.3451(9); Rh1–Cl1–C2/C2A, 118.3(3)/117.8(4); Rh1–N20, 2.006(2); N20–Rh1–Cl1, 169.81(7); Rh1–P2, 2.2690(8); Rh1–P3, 2.2985(9); P2–Rh1–P3, 161.45(3); **4**, Rh1–Cl1, 2.3158(13); Rh1–C2, 2.029(5); C2–Rh1–Cl1, 101.97(16); Rh1–N20, 2.020(4); N20–Rh1–Cl1, 168.21(13); Rh1–P2, 2.3386(12); Rh1–P3, 2.3338(13); P2–Rh1–P3, 161.92(5).

**Scheme 2:**
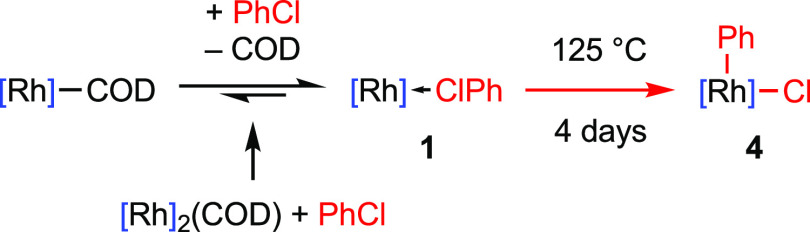
Synthesis and Reactivity of **1** Reactions in PhCl at room temperature unless otherwise stated, [Rh] = [Rh(PONOP-*t*Bu)][BAr^F^_4_].

We have previously established that the cyclooctadiene bridged rhodium(I) dimer [{Rh(PONOP-*t*Bu)}_2_(μ-η^2^:η^2^-COD)][BAr^F^_4_]_2_ is a convenient latent source of the {Rh(PONOP-*t*Bu)}^+^ fragment in solution.^17,18^ Targeting synthesis of the *κ*_Cl_–chlorobenzene adduct **1** in the first instance, this rhodium(I) dimer (20 mM/Rh) was consequently dissolved in chlorobenzene at room temperature. Analysis by ^1^H and ^31^P NMR spectroscopy indicated liberation of cyclooctadiene into solution and generation of a 4:1 equilibrium mixture of **1** (δ_31P_ 203.0, ^1^*J*_RhP_ = 136 Hz) and [Rh(PONOP-*t*Bu)(η^2^-COD)][BAr^F^_4_] (δ_31P_ 202.3, ^1^*J*_RhP_ = 135 Hz) after 6 h (Scheme 2). Analytically pure material of **1** was subsequently isolated in good yield (74%) after two consecutive recrystallizations from chlorobenzene/hexane, to perturb the equilibrium toward the desired product through removal of cyclooctadiene, and fully characterized (Figure 1).

Structural analysis of **1** in the solid state confirmed κ_Cl_-coordination of chlorobenzene (Figure 1). The metal adopts a pseudo square planar geometry, with the datively bound chlorine atom associated with a distinctly non-linear N20–Rh2–Cl1 angle of 168.21(13)° and the aryl substituent skewed to one side of the coordination plane [Rh1–Cl1–C2(aryl) = 101.97(16)°]. The Rh1–Cl1 bond length of 2.3451(9) Å is similar to that reported for **A** [2.350(2) Å]^10^ but considerably shorter than observed in the rhodium(III) pincer complex [Rh(POP-Ar^F^)H_2_(*κ*_Cl_–ClPh)][BAr^F^_4_] [POP-Ar^F^ = 4,5-bis(di-3,5-bis(trifluoromethyl)phenylphosphino)-9,9-dimethylxanthene; 2.5207(12) Å], the only crystallographically characterized rhodium precedent for κ_Cl_-coordination of an aryl chloride to our knowledge.^19^

Facile ligand exchange (vide infra) limited analysis of **1** by NMR spectroscopy to data acquired using chlorobenzene as the solvent. Nevertheless, observation of time-averaged *C*_2*v*_ symmetry indicates a highly fluxional structure and **1** was found to be otherwise stable for extended periods of time in chlorobenzene at room temperature (no change after 3 days, light/dark). Prolonged heating of **1** (20 mM) in chlorobenzene at 125 °C did, however, result in smooth conversion into the rhodium(III) derivative [Rh(PONOP-*t*Bu)(Ph)Cl][BAr^F^_4_] **4** (δ_31P_ 182.5, ^1^*J*_RhP_ = 103 Hz; Scheme 2). The reaction exhibits pseudo-first-order kinetics under these conditions (*t*_1/2_ = 14 h; Figure S7) and **4** was obtained in a quantitative spectroscopic yield after 4 days. The reaction was unaffected by the addition of TEMPO as a radical scavenger. Complex **4** was subsequently isolated in 60% yield and fully characterized in solution and the solid state. In line with structurally related {Rh^I^(pincer)} precedents,^3,4^ we propose that **4** is the product of a concerted—three-center-two-electron—oxidative addition of the C(sp^2^)–Cl bond (BDE = 400 kJmol^–1^).^20^ Mechanistic work on the activation of aryl halides by Ozerov and co-workers points toward an early transition state for concerted insertion into the C(sp^2^)–Cl bond, and explicit isolation of the κ_Cl_-coordinated chlorobenzene adduct **1** supports this conclusion.^3^

A square pyramidal metal geometry is observed for **4** in the solid state, with the aryl ligand in the apical position [Rh1–C2 = 2.029(5) Å] (Figure 1). In line with formation of a covalent bond and the increased oxidation state, the Rh1–Cl1 bond length [2.3158(13) Å] is contracted relative to **1** [2.3451(9) Å]. Complex **4** is stable in dichloromethane solution, with no onward reactivity detected after 24 h at room temperature (light/dark/presence of TEMPO). *C*_*s*_ symmetry is retained in CD_2_Cl_2_ solution with a downfield doublet of triplet aryl ^13^C resonance at δ 141.9 (^1^*J*_RhC_ = 34 Hz, ^2^*J*_PC_ = 8 Hz) and the reduction of the ^1^*J*_RhP_ coupling constant from 136 to 103 Hz fully consistent with the assigned structure.^21^

**Figure 2:**
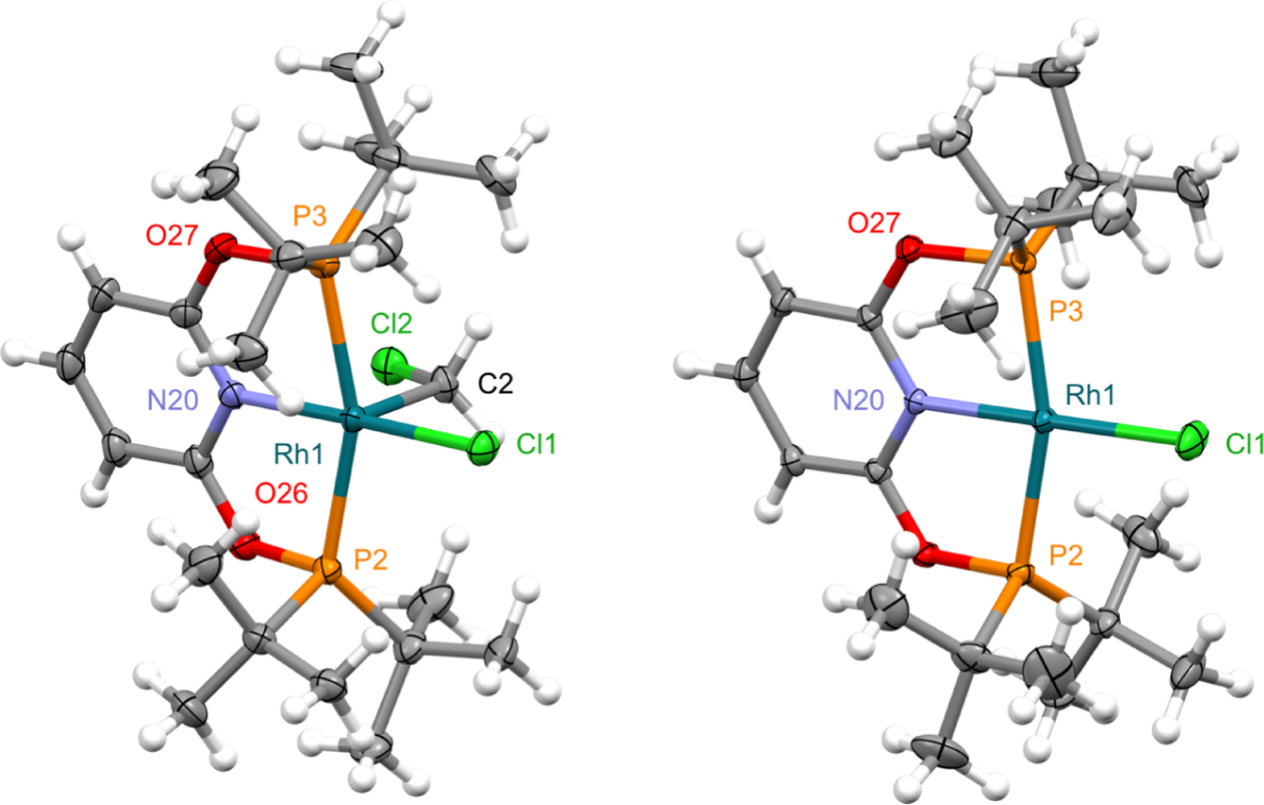
Solid-state structures of **5** (left) and **6** (right) with thermal ellipsoids at 30% probability. The former was established using a 9:1 co-crystalline sample of **5** and **6**.^22^ CH_2_Cl_2_ solvate (**6**) and anions omitted. Selected bond lengths (Å) and angles (°): **5**, Rh1–Cl1, 2.3032(9); Rh1–C2, 2.079(4); C2–Rh1–Cl1, 88.72(12); Rh1–N20, 2.035(3); N20–Rh1–Cl1, 175.41(10); Rh1–P2, 2.3370(10); Rh1–P3, 2.3518(9); P2–Rh1–P3, 160.53(3); **6**, Rh1–Cl1, 2.2956(6); Rh1–N20, 2.023(2); N20–Rh1–Cl1, 178.11(5); Rh1–P2, 2.3008(5); Rh1–P3, 2.3049(6); P2–Rh1–P3, 162.40(2).

**Scheme 3:**
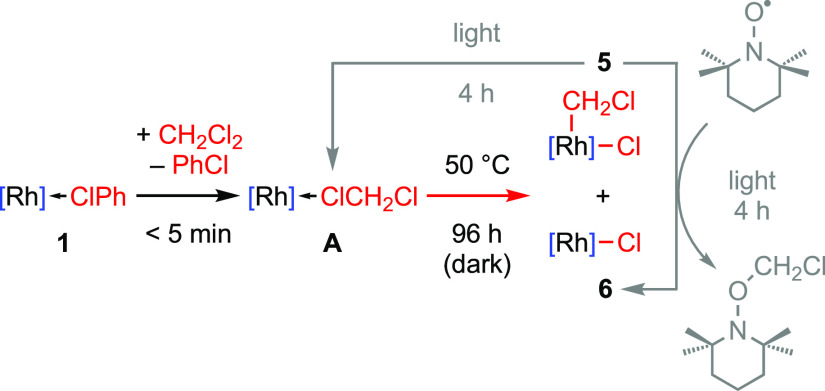
Synthesis and Reactivity of **A** Reactions in CH_2_Cl_2_/CD_2_Cl_2_ at room temperature unless otherwise stated, [Rh] = [Rh(PONOP-*t*Bu)][BAr^F^_4_].

Going forward, **1** proved to be the precursor of choice for synthesis of the other κ_Cl_–chlorocarbon targets through ligand substitution. Notably, given the forcing conditions required to bring about the formation of **4**, the chlorobenzene byproduct generated in this procedure is unlikely to participate in any further metal-based reactivity. Turning to the activation of homolytically weaker C(sp^3^)–Cl bonds, we next chose to re-examine the synthesis and reactivity of **A**, first prepared by Weller and co-workers.^10^ Gratifyingly, dissolution of **1** (20 mM) in dichloromethane resulted in quantitative conversion into **A** upon mixing at room temperature (Scheme 3). Spectroscopic data agree with the literature (time averaged *C*_2*v*_ symmetry; δ_31P_ 204.5, ^1^*J*_RhP_ = 136 Hz) and, in our hands, analytically pure material could be obtained by recrystallization from dichloromethane/hexane in 86% isolated yield. Samples of **A** prepared from CH_2_Cl_2_ are instantaneously converted into the d_2_-isotopologue upon dissolution in CD_2_Cl_2_ (20 mM) with concomitant liberation of CH_2_Cl_2_. Otherwise, no appreciable onward reactivity was detected by ^1^H and ^31^P NMR spectroscopy when left to stand at room temperature in the light for 24 h. In the absence of light, however, 3% conversion to a new species characterized by a doublet ^31^P resonance at δ 182.0 with an appreciably reduced ^1^*J*_RhP_ coupling constant of 104 Hz was observed under otherwise equivalent conditions. A follow-up experiment involving heating a 20 mM CD_2_Cl_2_ solution of **A** at 50 °C in the dark confirmed this onward reactivity, which was found to proceed with pseudo-first-order kinetics (*t*_1/2_ = 14 h, Figure S32) and resulted in complete consumption of the rhodium(I) starting material within 96 h. Analysis of the resulting reaction mixture by ^1^H and ^31^P NMR spectroscopy indicated formation of an 8:2 mixture of organometallic species, which we ultimately identified as the rhodium(III) complex [Rh(PONOP-*t*Bu)(CD_2_Cl)Cl][BAr^F^_4_] d_2_-**5** and the rhodium(II) metalloradical [Rh(PONOP-*t*Bu)Cl][BAr^F^_4_] **6** (Scheme 3 and Figure 2).

Complex **5** is the PONOP pincer homologue of **B** (Scheme 1) and was isolated in highest purity by heating a 50 mM CH_2_Cl_2_ solution of **A** at 50 °C in the dark for 96 h (9:1 ratio of **5**:**6**), followed by recrystallization from CH_2_Cl_2_/hexane at −30 °C in the dark (co-crystallization of **5**:**6** in a 9:1 ratio).^22^ This sample was sufficiently enriched in **5** to permit structural elucidation in CD_2_Cl_2_ solution by ^1^H, ^13^C, and ^31^P NMR spectroscopy (in the dark) despite contamination by paramagnetic **6**. Complex **5** is characterized by *C*_*s*_ symmetry, with the coordination of the chloroalkyl ligand confirmed by a 2H triplet of doublet resonance at δ 5.65 (^3^*J*_PH_ = 6.8, ^2^*J*_RhH_ = 3.4 Hz) and doublet of triplets ^13^C resonance at δ 48.1 (^1^*J*_RhC_ = 30, ^2^*J*_PC_ = 5 Hz).^23^ Additionally, the ^31^P NMR signature (δ_31P_ 181.9, ^1^*J*_RhP_ = 104 Hz) is strikingly similar to **4** (δ_31P_ 182.8, ^1^*J*_RhP_ = 103 Hz). The proposed structure of **5** is further borne by crystallographic analysis of the co-crystalline mixture (Figure 2). As for **B**,^10^ the solid-state structure of **5** is notable for the adoption of a square pyramidal metal geometry, with the chloroalkyl ligand in the apical position [Rh1–C2 = 2.079(4) Å] and the chloride projected over the pyridine donor [Cl1–Rh1–C2–Cl2 dihedral angle of 172.0(2)°]. Co-crystallization of **B** and **5** with structurally related [Rh(PNP-*t*Bu)(H)Cl][BAr^F^_4_] and **6**, respectively, prevents meaningful analysis of their metrics and comparison to **4**: an unusual and slightly disturbing coincidence.

Assignment of **6** as a metalloradical was informed by the detection of a very broad ^1^H resonance at δ 25 during in situ analysis of the reaction of **A** with dichloromethane, the aforementioned work by Hulley and co-workers,^9^ and isolation of the PNP homologue [Rh(PNP-*t*Bu)Cl][BAr^F^_4_] **C** by Milstein and co-workers 15 years ago.^24^ Independent synthesis of purple **6** by one-electron oxidation of [Rh(PONOP-*t*Bu)Cl] with Fc[BAr^F^_4_] (*E*_1/2_ = −0.01 V vs Fc/Fc^+^, 48% yield; Fc = ferrocene) corroborates this assignment and enabled full characterization in solution and the solid state. No ^31^P resonance could be located for **6** between δ −600 and 600, but paramagnetically shifted *t*Bu (δ 24.6), 3-py (δ 1.5), and 4-py (δ −17.3) resonances are evident in the ^1^H NMR spectrum. The crystal structure shows **6** with a square planar metal geometry and a Rh1–Cl1 bond length of 2.2956(6) Å that is considerably shorter than that observed in both the rhodium(I) precursor [2.3562(7) Å] and rhodium(III) aryl **4** [2.3158(13) Å, Figure 1].^25^ This metric may help reconcile the short ensemble value for the Rh1–Cl1 bond in the co-crystalline sample of **5** and **6** [2.3032(9) Å] compared to that in **4** [2.3158(13) Å]. A less pronounced rhodium(I/II) contraction was observed for **C** [2.381(1)/2.332(1) Å] and attributed to enhanced chloride-to-rhodium π-donation.^24^

**Figure 3:**
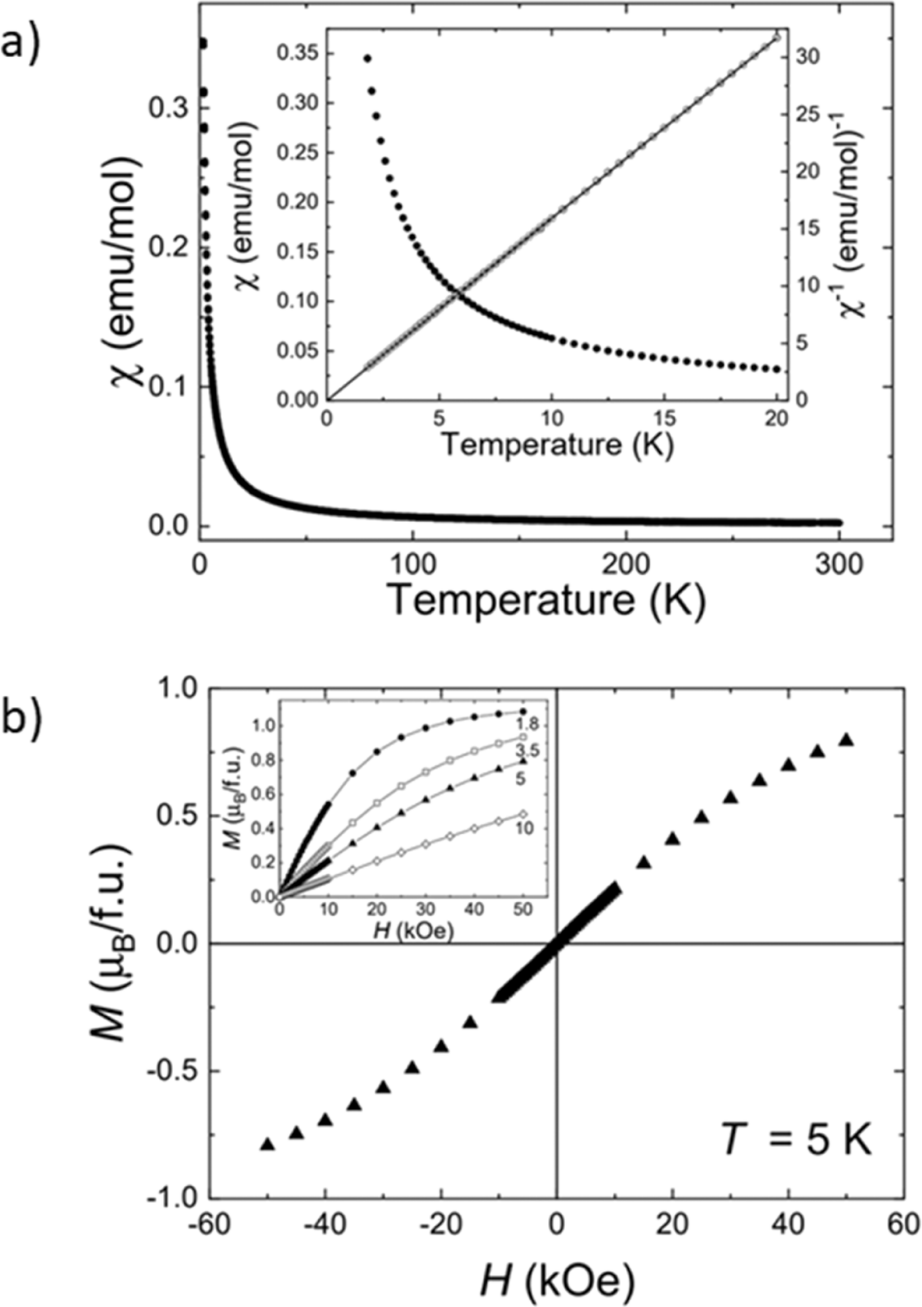
(a) Temperature dependence of the dc magnetic susceptibility χ_dc_(*T*) [●] and the inverse dc magnetic susceptibility vs temperature **χ**_**DC**_^**–1**^**(*T*)** [○] for **6**. The data were collected while cooling in an applied field, *H*, of 1 kOe. The solid line shows a fit using a Curie–Weiss law  between 2 and 20 K. (b) Magnetization vs applied field for **6** at 5 K. The inset shows single quadrant *M*(*H*) curves at 1.8 [●], 3.5 [□], 5 [▲], and 10 K [◊].

Magnetic susceptibility measurements were performed to investigate the spin state of **6**. Figure 3a shows the temperature dependence of dc magnetic susceptibility, χ_dc_(*T*), between 2 and 300 K and at low temperature in the inset. Complex **6** is paramagnetic with no signs of magnetic order or magnetic field history. A fit using a Curie–Weiss model gave an effective moment of 2.22(2) μ_B_ slightly higher than 1.73 μ_B_ expected for a spin *S* = 1/2 ion. Similar values have been reported for rhodium(II) in a square planar environment, including **C**.^24,26^ A Weiss temperature, θ_W_, of +0.007(5) K is also consistent with the absence of magnetic order. Magnetization measurements are linear in magnetic fields below 10 kOe with no hysteresis. Figure 3b shows a four quadrant *M*(*H*) curve collected at 5 K. At higher fields, the magnetization tends to saturate. The inset of Figure 3b shows that **6** has a saturation moment of approximately 1.10(5) μ_B_ at 1.8 K, which is consistent with *S* = 1/2.

Mixtures of **5** and **6** (9:1 ratio, [Rh] = 20 mM) in CD_2_Cl_2_ remained unchanged (with no H/D scrambling of the methylene group) over 48 h at room temperature in the dark, indicating that the rhodium(III) complex is thermodynamically stable in solution. Upon exposure of the solution to light, however, complete reversion of **5** into **A** was observed within 4 h at room temperature (Scheme 3). This photo-induced reductive elimination process reconciles the apparent lack of reactivity of **A** when exposed to light in solution and suggests that the rhodium(I)–dichloromethane complex should be viewed as a photo-stationary rather than a thermodynamic ground state. To interrogate the mechanism associated with reversion of **5** to **A**, the experiment was repeated in the presence of TEMPO as a radical trapping agent. No reaction was apparent in the dark, but exposure to light resulted in complete conversion of **5** into **6** within 4 h at room temperature with contaminant generation of a species assigned as TEMPO–CH_2_Cl.^27^ Control experiments involving heating isolated **6** in CD_2_Cl_2_ at 50 °C for 24 h in the presence or absence of light were conducted, but no onward reactivity of the metalloradical was detected. Based on these observations and recognizing that oxidative addition and reductive elimination processes follow the same pathway, we propose that **5** is the product of non-chain radical oxidative addition of the C(sp^3^)–Cl bond (BDE = 338 kJmol^–1^).^1,20^ Interpreted this way, the formation of **6** during the reaction is ascribed to incomplete recombination with the ClCH_2_^•^ radical.^28^ While it is currently unclear what organic byproduct is formed alongside **6**, we note that thermolysis of **A** in the solid state (110 °C for 18 h) also gives a mixture of **5** and **6**.

**Figure 4:**
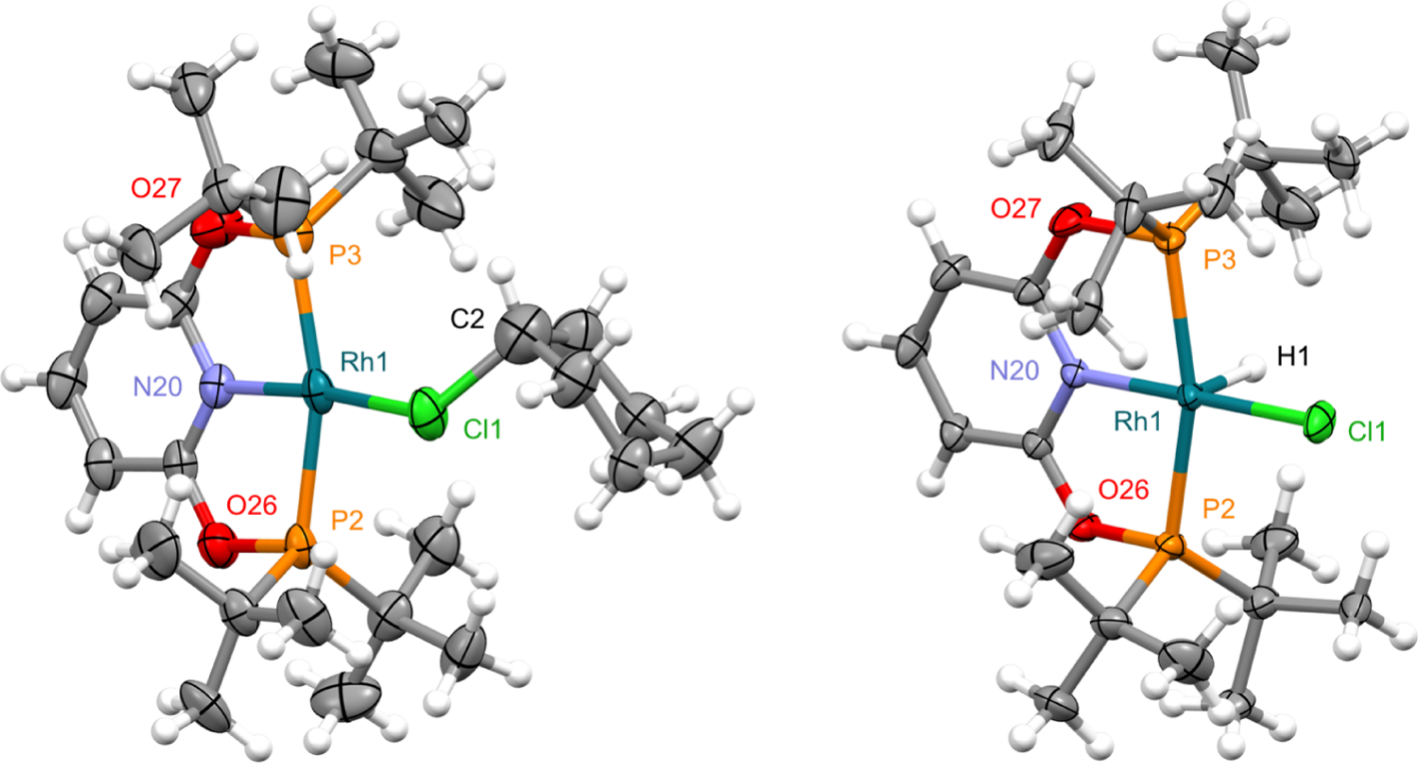
Solid-state structures of **2** (left) and **7** (right) with thermal ellipsoids at 30% probability. Only one of the two unique cations is shown for **2** (*Z*′ = 2); the hydride in **7** was located off the Fourier difference map and the Rh–H distance was thereafter tightly restrained; minor disordered components (Cy in **2**, 3× *t*Bu in **7**) and anions omitted. Selected bond lengths (Å) and angles (°): **2** as shown, Rh1–Cl1/Cl1A, 2.370(9)/2.308(15); Rh1–Cl1–C2/Cl1A-C2A, 126.5(6)/114.4(9); Rh1–N20, 2.015(5); N20–Rh1–Cl1/Cl1A, 173.0(3)/170.8(4); Rh1–P2, 2.281(2); Rh1–P3, 2.283(2); P2–Rh1–P3, 161.90(7); **2** other cation, Rh1B–Cl1B/Cl1C, 2.376(3)/2.402(4); Rh1B–Cl1B–C2B/Cl1C–C2C, 115.9(5)/115.5(9); Rh1B–N20B, 2.003(5); N20B–Rh1B–Cl1B/Cl1C, 160.4(2)/164.7(2); Rh1B–P2B, 2.274(2); Rh1B–P3B, 2.271(2); P2B–Rh1B–P3B, 162.24(7); **7**, Rh1–Cl1, 2.3049(8); Rh1–H1, restrained to 1.69; H1–Rh1–Cl1 96.0; Rh1–N20, 2.018(2); N20–Rh1–Cl1, 178.53(7); Rh1–P2, 2.2913(8); Rh1–P3, 2.2988(8); P2–Rh1–P3, 163.09(3).

**Scheme 4:**
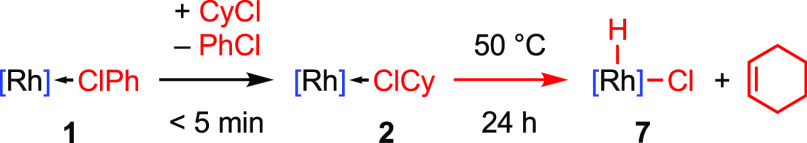
Synthesis and Reactivity of **2** Reactions in CyCl at room temperature unless otherwise stated, [Rh] = [Rh(PONOP-*t*Bu)][BAr^F^_4_].

Moving onto examination of other alkyl chlorides, dissolution of **1** (20 mM) in chlorocyclohexane resulted in quantitative spectroscopic conversion into the corresponding rhodium(I) κ_Cl_-bound complex **2** (time averaged *C*_2*v*_ symmetry, δ_31P_ 204.5, ^1^*J*_RhP_ = 138 Hz) upon mixing at room temperature (Scheme 4). Complex **2** is sufficiently stable at room temperature to permit isolation from solution, and analytically pure material was obtained on a preparative scale by recrystallization from chlorocyclohexane/hexane in 79% yield. Crystals grown in this way were suitable for analysis by X-ray diffraction, and the resulting solid-state structure revealed chemically similar but unique cations (*Z*′ = 2) and enabled intact coordination of chlorocyclohexane (heavily disordered) to be corroborated with Rh–Cl bond lengths ranging between 2.31 and 2.40 Å (Figure 4). We are not aware of any crystallographic precedents for κ_Cl_-coordination of chlorocyclohexane (CSD v.5.43, update June 2022). Upon standing in chlorocyclohexane solution at room temperature for 24 h, partial conversion of **2** into the new rhodium(III) hydride [Rh(PONOP-*t*Bu)(H)Cl][BAr^F^_4_] **7** (*C*_*s*_ symmetry; δ_31P_ 197.1, ^1^*J*_RhP_ = 100 Hz; δ_1H_ −26.12, ^1^*J*_RhH_ = 42.3, ^2^*J*_PH_ = 10.6 Hz) was observed (ca. 10% conversion). Quantitative spectroscopic conversion into **7** and 1 equiv of cyclohexene was subsequently achieved within 24 h by heating **4** (20 mM) in chlorocyclohexane at 50 °C (Scheme 4). The dehydrochlorination was unaffected by the presence of light.

**Scheme 5:**
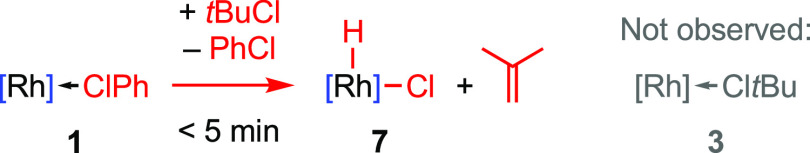
Attempted Synthesis of **3** Reaction in *t*BuCl at room temperature, [Rh] = [Rh(PONOP-*t*Bu)][BAr^F^_4_].

A considerably faster dehydrochlorination resulted when **1** (20 mM) was dissolved in 2-chloro-2-methylpropane. The putative κ_Cl_–chlorocarbon complex **3** could not be detected and instead complete conversion into **7** and isobutene was observed upon mixing at room temperature (Scheme 5). This proved to be our method of choice for the preparation of **7**, which was isolated as an analytically pure material in 87% yield following removal of volatiles and recrystallization from CH_2_Cl_2_/hexane. Crystals grown in this way were suitable for analysis by X-ray diffraction and the solid-state structure is fully consistent with our assignment (Figure 4). In particular, while requiring tight restraints, the hydride ligand was located off the Fourier difference map during the refinement. The component Rh1–Cl1 bond length [2.3049(8) Å] is notably shorter than that in rhodium(III) aryl **4** [2.3158(13) Å] and approaching that observed in the rhodium(II) metalloradical **6** [2.2956(6) Å]. Indeed, we cannot exclude the possibility that the single crystal analyzed was free of co-crystallized **6**.^29^

**Scheme 6:**
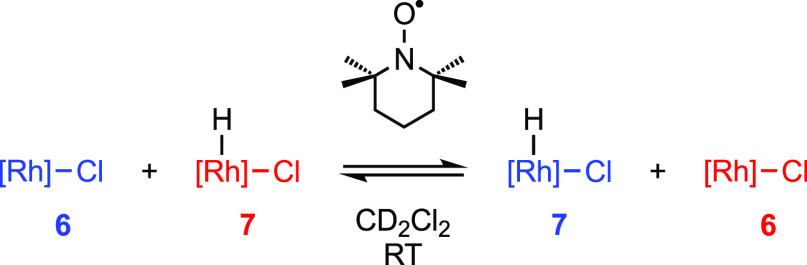
Hydrogen Atom Transfer Between **6** and **7** [Rh] = [Rh(PONOP-*t*Bu)][BAr^F^_4_].

Extrapolating from our mechanistic work with **A**, we propose that activation of chlorocyclohexane and 2-chloro-2-methylpropane involves homolytic cleavage of the C(sp^3^)–Cl bonds (BDE = 356 and 352 kJmol^–1^, respectively)^20^ through chlorine atom abstraction by the latent {Rh(PONOP)}^+^ fragment, generating **6** and an alkyl radical. Compared to methyl chloride, the cyclohexyl and *tert*-butyl radicals are more thermodynamically stable (Δ_f_*H*^0^ = +117, +75, and +48 kJmol^–1^, respectively) and characterized by considerably weaker C–H bonds (BDE = 427, 138, and 153 kJmol^–1^, respectively).^20^ Informed by these data, we suggest that formation of **7** and alkene occurs by hydrogen atom abstraction from the alkyl radical, rather than direct C-radical recombination with **6** and β-H elimination. Supporting this hypothesis, addition of 0.5–2.0 equiv of TEMPO to **7** (20 mM) in CD_2_Cl_2_ resulted in hydrogen atom abstraction [BDE(O–H) = 292 kJmol^–1^]^20^ and establishment of a dynamic equilibrium involving hydrogen atom transfer between **6** and **7** on the ^1^H NMR time scale at 298 K (400 MHz; Scheme 6). The latter is most notably evidenced by the presence of a board 36H resonance at δ 13.2 (∼ equally weighted average of the *t*Bu signals of **6** and **7**), which was sharper with higher concentrations of added TEMPO (Figure S70). No hydrogen atom shuttling was observed when a 1:1 mixture of **6** and **7** in CD_2_Cl_2_ was prepared in the absence of TEMPO, confirming that the aminoxyl radical is required to mediate the process. Moreover, 40% conversion of **6** into **7** was observed after heating with 0.9 equiv of dihydroanthracene in CD_2_Cl_2_ at 50 °C for 2 weeks.

## 3 Conclusions

As a platform for investigating C–Cl bond activation reactions, we have developed operationally simple procedures for the generation of low-valent rhodium κ_Cl_–chlorocarbon complexes of the form [Rh(PONOP-*t*Bu)(*κ*_Cl_–ClR)][BAr^F^_4_] (R = CH_2_Cl, **A**; Ph, **1**; Cy, **2**; *t*Bu, **3**) in solution. Notably, the chlorobenzene derivative **1** was isolated by displacement of cyclooctadiene from [{Rh(PONOP-*t*Bu)}_2_(μ-η^2^:η^2^-COD)][BAr^F^_4_]_2_ and serves as a well-defined precursor for the other κ_Cl_–chlorocarbon complexes through facile ligand substitution, with only innocuous chlorobenzene as a byproduct. In this way, the first rhodium(I) κ_Cl_–complexes of chlorobenzene and chlorocyclohexane have been isolated and structurally characterized in the solid state by single-crystal X-ray diffraction.

**Scheme 7:**
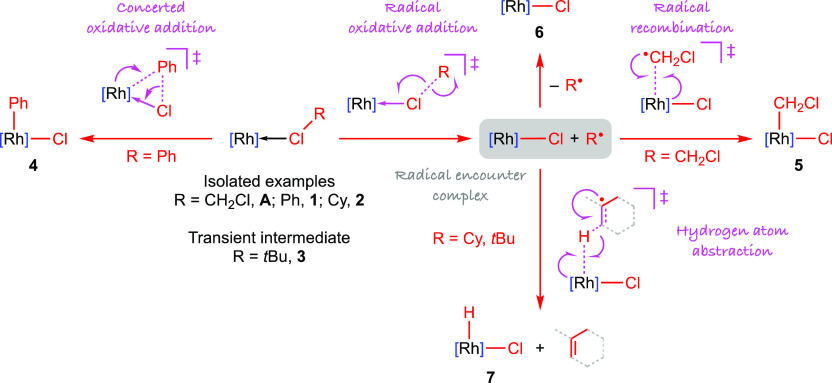
Summary of Findings [Rh] = [Rh(PONOP-*t*Bu)][BAr^F^_4_].

Complex **1** is stable under ambient conditions, but onward C–Cl bond oxidative addition of chlorobenzene to the rhodium(I) pincer affording [Rh(PONOP-*t*Bu)(Ph)Cl][BAr^F^_4_] **4** could be induced by prolonged heating in the neat chlorocarbon at 125 °C (Scheme 7). This reaction proceeded in one step and was unaffected by addition of TEMPO. Informed by these reaction characteristics, literature precedents, and the robust nature of the C(sp^2^)–Cl bond, we propose that formation of **4** occurs by a concerted oxidative addition mechanism. Consistent with their homolytically weaker C(sp^3^)–Cl bonds, activation of dichloromethane (96 h at 50 °C in the dark), chlorocyclohexane (24 h at 50 °C), and 2-chloro-2-methylpropane (<5 min at RT) by the rhodium(I) pincer occurred under considerably milder conditions and were rationalized by radical mechanisms that commence with chloride atom abstraction and involve generation of the rhodium(II) metalloradical [Rh(PONOP-*t*Bu)Cl][BAr^F^_4_] **6** (Scheme 7). For dichloromethane, subsequent recombination of **6** with the ClCH_2_^•^ radical and formation of the rhodium(III) product [Rh(PONOP-*t*Bu)(CH_2_Cl)Cl][BAr^F^_4_] **5** is masked by rapid photo-induced reductive elimination when the reaction is conducted in the light. The metalloradical **6** was directly observed as a minor reaction component in the dark. Net dehydrochlorination affording [Rh(PONOP-*t*Bu)(H)Cl][BAr^F^_4_] **7** and an alkene byproduct resulted when **1** was dissolved in chlorocyclohexane and 2-chloro-2-methylpropane. With these substrates, we believe that hydrogen atom abstraction from the corresponding alkyl radicals is considerably faster than C-radical recombination with **6**. This suggestion is supported by the observation of dynamic hydrogen atom transfer between **6** and **7** on the ^1^H NMR time scale at 298 K in the presence of TEMPO (Scheme 6).

## 4 Experimental Section

### 4.1 General Methods

All manipulations were performed in the light under an atmosphere of argon using Schlenk and glovebox techniques unless otherwise stated. Glassware was oven-dried at 150 °C overnight and flame-dried under vacuum prior to use. Molecular sieves were activated by heating at 300 °C in vacuo overnight. Anhydrous CH_2_Cl_2_ and hexane were purchased from commercial suppliers, freeze–pump–thaw degassed, and stored over activated 3 Å molecular sieves. Chlorobenzene, chlorocyclohexane, 2-chloro-2-methylpropane, and CD_2_Cl_2_ were freeze–pump–thaw degassed and stored over activated 3 Å molecular sieves. 1,2-Difluorobenzene was stirred over neutral aluminum oxide, filtered, dried over CaH_2_, vacuum distilled, freeze–pump–thaw degassed, and then stored over activated 3 Å molecular sieves.^30^ [{Rh(PONOP-*t*Bu)}_2_(μ-η^2^:η^2^-COD)][BAr^F^_4_]_2_ was prepared from [Rh(COD)_2_][BAr^F^_4_]^31^ and PONOP-*t*Bu^32^ in 1,2-difluorobenzene using a procedure developed by our group.^17^ [Rh(PONOP-*t*Bu)Cl]^33^ and Fc[BAr^F^_4_]^34^ were prepared using literature protocols. All other reagents are commercial products and were used as received. NMR spectra were recorded on Bruker spectrometers under argon at 298 K unless otherwise stated. Chemical shifts are quoted in parts per million, and coupling constants are given in hertz. Virtual coupling constants are reported as the separation between the first and third lines.^21^ NMR spectra in non-deuterated solvents were recorded using an internal capillary of C_6_D_6_. High-resolution electrospray ionization mass spectrometry (HR-ESI-MS) spectra were recorded on a Bruker MaXis mass spectrometer. Microanalyses were performed by Elemental Microanalysis Ltd. Measurements of dc magnetization were performed using a Quantum Design MPMS-5S SQUID (superconducting quantum interference device) magnetometer. The powdered sample was immobilized in a small quantity of *n*-eicosane and sealed in a quartz tube. Measurements of dc magnetic susceptibility, χ_dc_, versus temperature, *T*, were performed between 2 and 300 K in zero-field-cooled warming (ZFCW) and field-cooled cooling (FCC) modes in applied fields, *H*, between 50 Oe and 5 kOe. Magnetization versus field measurements were performed at fixed temperatures in magnetic fields between −50 and 50 kOe.

### 4.2 NMR Scale Reaction of [{Rh(PONOP-*t*Bu)}_2_(μ-η^2^:η^2^-COD)][BAr^F^_4_]_2_ with PhCl

To a J. Young’s valve NMR tube charged with [{Rh(PONOP-*t*Bu)}_2_(μ-η^2^:η^2^-COD)][BAr^F^_4_]_2_ (14.1 mg, 5.0 μmol) was added PhCl (0.5 mL). The resulting orange homogeneous solution was analyzed in situ using ^1^H and ^31^P NMR spectroscopy, with constant mixing at room temperature when not in the spectrometer. Liberation of COD and formation of [Rh(PONOP) (*κ*_Cl_–ClPh)][BAr^F^_4_] [**1**; δ_31P_ 203.0 (d, ^1^*J*_RhP_ = 136)] were observed, with a 4:1 equilibrium mixture of **1** and [Rh(PONOP)(η^2^-COD)][BAr^F^_4_] [δ_31P_ 202.3 (d, ^1^*J*_RhP_ = 135)]^17^ obtained after 6 h.

### 4.3 Preparation of [Rh(PONOP-*t*Bu)(*κ*_Cl_–ClPh)][BAr^F^_4_] **1**

To a flask charged with [{Rh(PONOP-*t*Bu)}_2_(μ-η^2^:η^2^-COD)][BAr^F^_4_]_2_ (100.7 mg, 35.5 μmol) was added PhCl (10 mL) with vigorous stirring. The resulting orange solution was left to stand for 18 h at room temperature, and the analytically pure material obtained as orange crystals after two consecutive crystallizations from ClPh/hexane at room temperature. Yield: 77.4 mg (52.4 μmol, 74%). Crystals grown in this way were suitable for analysis by X-ray diffraction.

^1^H NMR (400 MHz, PhCl; selected data): δ 8.04–8.10 (m, 8H, Ar^F^), 7.43 (br, 4H, Ar^F^), 6.12 (d, ^3^*J*_HH_ = 8.1, 2H, 3-py), 0.91 (vt, *J*_PH_ = 14.7, 36H, *t*Bu). No paramagnetic signals observed in the range −50 to +50 ppm.

^31^P{^1^H} NMR (162 MHz, PhCl): δ 203.0 (d, ^1^*J*_RhP_ = 138).

Anal. Calcd for C_59_H_56_BClF_24_NO_2_P_2_Rh (1478.18 gmol^–1^): C, 47.94; H, 3.82; N, 0.95. Found: C, 48.16; H, 3.84; N, 1.00.

### 4.4 NMR Scale Reactions of [Rh(PONOP-*t*Bu)(*κ*_Cl_–ClPh)][BAr^F^_4_] **1**

Reactions were performed within J. Young’s valve NMR tubes using 20 mM solutions of **1** (14.8 mg, 10.0 μmol) in the respective solvent (0.5 mL) and monitored in situ using ^1^H and ^31^P NMR spectroscopy.

#### 4.4.1 Stability at Room Temperature in PhCl

No onward reaction of **1** was apparent upon standing in PhCl at room temperature for 72 h, both in the presence and absence of light (orange solution).

#### 4.4.2 Stability at 125 °C in PhCl

Heating **1** in PhCl at 125 °C for 96 h in the dark resulted in quantitative formation of [Rh(PONOP-*t*Bu)(Ph)Cl][BAr^F^_4_] [**4**; δ_31P_ 182.5 (d, ^1^*J*_RhP_ = 103); dark orange solution]. The same outcome was observed when repeated in the presence of light.

#### 4.4.3 Stability at 125 °C in PhCl in the Presence of TEMPO.

Heating **1** and TEMPO (1 equiv) in PhCl at 125 °C for 24 h in the dark resulted in the partial formation (ca. 40%) of [Rh(PONOP-*t*Bu) (Ph)Cl][BAr^F^_4_] [**4**; δ_31P_ 182.7 (d, ^1^*J*_RhP_ = 102); dark orange solution]. No paramagnetic species were observed by ^1^H NMR spectroscopy.

#### 4.4.4 Stability at Room Temperature in CD_2_Cl_2_

Dissolution of **1** in CD_2_Cl_2_ at room temperature resulted in displacement of PhCl and quantitative formation of [Rh(PONOP-*t*Bu)(*κ*_Cl_–ClCD_2_Cl)][BAr^F^_4_] [d_2_-**5**; δ_31P_ 204.5 (d, ^1^*J*_RhP_ = 136)] within 5 min (orange solution).

#### 4.4.5 Stability at Room Temperature in CyCl

Dissolution of **1** in CyCl at room temperature resulted in displacement of PhCl and quantitative formation of [Rh(PONOP-*t*Bu)(*κ*_Cl_–ClCy)][BAr^F^_4_] [**2**; δ_31P_ 204.5 (d, ^1^*J*_RhP_ = 138)] within 5 min (orange solution).

#### 4.4.6 Stability at Room Temperature in *t*BuCl

Dissolution of **1** in *t*BuCl at room temperature resulted in displacement of PhCl, generation of isobutene [δ_1H_ 5.00 (s, Me_2_C=CH_2_)], and quantitative formation of [Rh(PONOP-*t*Bu)(H)Cl][BAr^F^_4_] [**7**; δ_1H_ −25.89 (br d, ^1^*J*_RhH_ = 42.3, 1H, RhH), δ_31P_ 197.3 (d, ^1^*J*_RhP_ = 103)] within 5 min (yellow solution).

### 4.5 Preparation of [Rh(PONOP-*t*Bu)(Ph)Cl][BAr^F^_4_] **4**

A 20 mM solution of **4** in PhCl (0.5 mL) was prepared in situ as described above. Volatiles were removed in vacuo, and the analytically pure product obtained as dark orange crystals following recrystallization from CH_2_Cl_2_/pentane at 5 °C. Yield: 8.9 mg (6.0 μmol, 60%). Crystals suitable for analysis by X-ray diffraction were grown from PhCl/hexane at room temperature.

^1^H NMR (500 MHz, CD_2_Cl_2_): δ 8.10 (t, ^3^*J*_HH_ = 8.2, 1H, 4-py), 8.04 (br d, ^3^*J*_HH_ = 7.0, 1H, *o*-Ph), 7.70–7.76 (m, 8H, Ar^F^), 7.56 (br, 4H, Ar^F^), 7.17 (d, ^3^*J*_HH_ = 8.2, 2H, 3-py), 6.91–6.98 (m, 2H, *m*-Ph + *p*-Ph), 6.53 (ddt, ^3^*J*_HH_ = 9.1, 6.5, ^4^*J*_HH_ = 3.3, 1H, *m*-Ph), 5.01 (dt, ^3^*J*_HH_ = 8.6, ^4^*J*_HH_ = 2.5, 1H, *o*-Ph), 1.46 (vt, *J*_PH_ = 15.5, 18H, *t*Bu), 1.07 (vt, *J*_PH_ = 16.5, 18H, *t*Bu). No paramagnetic signals observed in the range −50 to +50 ppm.

^13^C{^1^H} NMR (126 MHz, CD_2_Cl_2_): δ 164.7 (s, 2-py), 162.3 (q, ^1^*J*_BC_ = 50, Ar^F^), 147.7 (s, 4-py), 141.9 (dt, ^1^*J*_RhC_ = 34, ^2^*J*_PC_ = 8, *i*-Ph), 140.0 (br, *o*-Ph), 135.4 (s, Ar^F^), 132.1 (br, *o*-Ph), 131.0 (s, *m*-Ph), 129.6 (s, *m*-Ph), 129.4 (qq, ^2^*J*_FC_ = 32, ^3^*J*_CB_ = 3, Ar^F^), 127.8 (s, *p*-Ph), 125.1 (q, ^1^*J*_FC_ = 272, Ar^F^), 118.0 (sept, ^3^*J*_FC_ = 4, Ar^F^), 106.9 (vt, *J*_PC_ = 4, 3-py), 44.4 (vt, *J*_PC_ = 10, *t*Bu{C}), 43.4 (vtd, *J*_PC_ = 10, ^2^*J*_RhC_ = 2, *t*Bu{C}), 28.4 (vt, *J*_PC_ = 5, *t*Bu{CH_3_}), 27.8 (vt, *J*_PC_ = 5, *t*Bu{CH_3_}).

^31^P{^1^H} NMR (162 MHz, CD_2_Cl_2_): δ 182.8 (d, ^1^*J*_RhP_ = 103).

HR-ESI-MS (positive ion, 4 kV): 614.1580 ([M]^+^, calcd for C_27_H_44_ClNO_2_P_2_Rh: 614.1585) *m/z*.

Anal. Calcd for C_59_H_56_BClF_24_NO_2_P_2_Rh (1478.18 gmol^–1^): C, 47.94; H, 3.82; N, 0.95. Found: C, 48.25; H, 3.83; N, 1.00.

### 4.6 NMR Scale Reactions of [Rh(PONOP-*t*Bu)(Ph)Cl][BAr^F^_4_] **4**

#### 4.6.1 Stability at Room Temperature in CD_2_Cl_2_

20 mM solutions of **4** (14.8 mg, 10.0 μmol) in CD_2_Cl_2_ (0.5 mL) were prepared within J. Young’s valve NMR tubes in the presence and absence of light and thereafter monitored in situ using ^1^H and ^31^P NMR spectroscopy. No significant onward reaction of **4** was apparent upon standing at room temperature for 24 h in the presence or absence of light (dark orange solution).

#### 4.6.2 Stability in the Presence of TEMPO in CD_2_Cl_2_

To a J. Young’s valve NMR tube charged with **4** (14.8 mg, 10.0 μmol) and TEMPO (1.6 mg, 10.2 μmol) was added CD_2_Cl_2_ (0.5 mL) at room temperature in the dark. The solution remained orange in color, and no onward reactivity with TEMPO was apparent from analysis in situ using ^1^H and ^31^P NMR spectroscopy after 24 h in the dark at room temperature. The same outcome was observed when the solution was subsequently exposed to light for 24 h.

### 4.7 Preparation of [Rh(PONOP-*t*Bu)(*κ*_Cl_–ClCH_2_Cl)][BAr^F^_4_] **A**

To a flask charged with [Rh(PONOP-*t*Bu)(*κ*_Cl_–ClPh)][BAr^F^_4_]_2_**1** (34.8 mg, 23.5 μmol) was added CH_2_Cl_2_ (1 mL). The resulting orange solution was left to stand for 5 min at room temperature, and the analytically pure material obtained as orange crystals upon crystallization from CH_2_Cl_2_/hexane at room temperature. Yield: 29.2 mg (20.1 μmol, 86%). Spectroscopic data are in agreement with the data reported in the literature for this compound.^10^ The use of paramagnetic ^1^H NMR spectroscopy in the dark confirmed that <1% [Rh(PONOP-*t*Bu)Cl][BAr^F^_4_] **6** was present.

Instantaneous exchange of coordinated dichloromethane, resulting in the liberation of CH_2_Cl_2_ and formation of d_2_-**A**, was apparent upon dissolution in CD_2_Cl_2_ by ^1^H NMR spectroscopy.

^1^H NMR (500 MHz, CD_2_Cl_2_): δ 7.73 (obscured t, ^3^*J*_HH_ = 8.1, 1H, 4-py), 7.70–7.74 (m, 8H, Ar^F^), 7.56 (br, 4H, Ar^F^), 6.73 (d, ^3^*J*_HH_ = 8.1, 2H, 3-py), 1.43 (vt, *J*_PH_ = 15.2, 36H, *t*Bu). Coordinated CH_2_Cl_2_ was not observed due to rapid ligand exchange.

^31^P{^1^H} NMR (161 MHz, CD_2_Cl_2_): δ 204.5 (d, ^1^*J*_RhP_ = 136).

### 4.8 NMR Scale Reactions of [Rh(PONOP-*t*Bu)(*κ*_Cl_–ClCX_2_Cl)][BAr^F^_4_] (X = H, A; D, d_2_-**A**)

#### 4.8.1 Stability at Room Temperature in CD_2_Cl_2_

20 mM solutions of d_2_-**A** (14.5 mg, 10.0 μmol) in CD_2_Cl_2_ (0.5 mL) were prepared within J. Young’s valve NMR tubes in the presence and absence of light and thereafter monitored in situ using ^1^H and ^31^P NMR spectroscopy. In the dark, standing at room temperature for 24 h resulted in partial conversion (3%) of d_2_-**A** into [Rh(PONOP-*t*Bu)(CD_2_Cl)Cl][BAr^F^_4_] [d_2_-**5**; δ_31P_ 182.0 (d, ^1^*J*_RhP_ = 104); orange solution] by ^1^H NMR spectroscopy. No onward reaction of d_2_-**A** was apparent upon standing at room temperature for 24 h in the light (orange solution).

#### 4.8.2 Stability at 50 °C in CD_2_Cl_2_

A 20 mM solution of d_2_-**A** (14.5 mg, 10.0 μmol) in CD_2_Cl_2_ (0.5 mL) was prepared within a J. Young’s valve NMR tube in the dark, heated at 50 °C in the dark, and periodically monitored in situ using ^1^H and ^31^P NMR spectroscopy at room temperature in the dark. After heating for 96 h, d_2_-**A** was completely consumed, and [Rh(PONOP-*t*Bu)(CD_2_Cl)Cl][BAr^F^_4_] [d_2_-**5**; δ_31P_ 182.0 (d, ^1^*J*_RhP_ = 104)] and [Rh(PONOP-*t*Bu)Cl][BAr^F^_4_] [**6**; δ_1H_ 24.53 (vbr, fwhm = 600 Hz, *t*Bu)] were observed in an 8:2 ratio by ^1^H NMR spectroscopy (orange solution). The formation of methyl chloride (∼δ_13C_ 25.1) or 1,2-dichloroethane (∼δ_13C_ 44.4) was not apparent from the analysis of the reaction mixture in the dark using ^13^C NMR spectroscopy.

#### 4.8.3 Solid-State Stability

Crystals of **A** (14.5 mg, 10.0 μmol) were heated in the solid state at 110 °C for 18 h, during which time they changed color from pale to dark orange. The sample was dissolved in CD_2_Cl_2_ (0.5 mL) and analyzed by ^1^H and ^31^P NMR spectroscopy in the dark, revealing generation of a 5:90:5 mixture of d_2_-**A**, [Rh(PONOP-*t*Bu)(CH_2_Cl)Cl][BAr^F^_4_] [**5**; δ_31P_ 182.0 (d, ^1^*J*_RhP_ = 104)] and [Rh(PONOP-*t*Bu)Cl][BAr^F^_4_] [**6**; δ_1H_ 24.39 (vbr, fwhm = 580 Hz, *t*Bu)] by ^1^H NMR spectroscopy.

#### 4.8.4 Characterization of [Rh(PONOP-*t*Bu)(CH_2_Cl)Cl][BAr^F^_4_] **5**

A 50 mM solution of **A** was prepared within a J. Young’s valve NMR tube in the dark by dissolution of **1** (36.4 mg, 24.6 μmol) in CH_2_Cl_2_ (0.5 mL). The resulting orange solution was heated at 50 °C in the dark and periodically monitored in situ using ^1^H and ^31^P NMR spectroscopy at room temperature in the dark. After being heated for 96 h, **A** was completely consumed, and **5** and [Rh(PONOP-*t*Bu)Cl][BAr^F^_4_] **6** were observed in a 9:1 ratio. Recrystallization from CH_2_Cl_2_/hexane in the dark afforded 29.5 mg of dark orange crystals, some of which were suitable for analysis by X-ray diffraction. Analysis of the sample in CD_2_Cl_2_ in the dark by ^1^H, ^13^C, and ^31^P NMR spectroscopy indicated co-crystallization of **5** and **6** in a 9:1 ratio.

Data for **5**: ^1^H NMR (500 MHz, CD_2_Cl_2_): δ 7.99 (t, ^3^*J*_HH_ = 8.2, 1H, py), 7.69–7.74 (m, 8H, Ar^F^), 7.55 (br, 4H, Ar^F^), 7.05 (d, ^3^*J*_HH_ = 8.2, 2H, py), 5.65 (dt, ^3^*J*_PH_ = 6.8, ^2^*J*_RhH_ = 3.4, 2H, CH_2_Cl), 1.64 (vt, *J*_PH_ = 16.1, 18H, *t*Bu), 1.43 (vt, *J*_PH_ = 15.6, 18H, *t*Bu).

^13^C{^1^H} NMR (126 MHz, CD_2_Cl_2_): δ 164.4 (s, 2-py), 162.3 (q, ^1^*J*_BC_ = 50, Ar^F^), 147.6 (s, 4-py), 135.3 (s, Ar^F^), 129.4 (qq, ^2^*J*_FC_ = 32, ^3^*J*_CB_ = 3 Ar^F^), 125.1 (q, ^1^*J*_FC_ = 272, Ar^F^), 118.0 (sept, ^3^*J*_FC_ = 4, Ar^F^), 106.6 (vt, *J*_PC_ = 4, 3-py), 48.1 (dt, ^1^*J*_RhC_ = 30, ^2^*J*_PC_ = 5, CH_2_Cl), 44.1 (vt, *J*_PC_ = 10, *t*Bu{C}), 42.9 (vtd, *J*_PC_ = 10, ^2^*J*_RhC_ = 2, *t*Bu{C}), 28.5 (vt, *J*_PC_ = 6, *t*Bu{CH_3_}), 28.4 (vt, *J*_PC_ = 6, *t*Bu{CH_3_}).

^31^P{^1^H} NMR (162 MHz, CD_2_Cl_2_): δ 181.9 (d, *J*_RhP_ = 104).

HR-ESI-MS (positive ion, 4 kV): 586.1034 ([M]^+^, calcd for C_22_H_41_Cl_2_NO_2_P_2_Rh: 586.1039) *m/z*.

Data for the mixture: Anal. Calcd for (C_54_H_53_BCl_2_F_24_NO_2_P_2_Rh)_0.9_(C_53_H_51_BClF_24_NO_2_P_2_Rh)_0.1_ (1445.60 gmol^–1^): C, 44.78; H, 3.68; N, 0.97. Found: C, 45.06; H, 3.61; N, 1.05.

### 4.9 Preparation of [Rh(PONOP-*t*Bu)Cl][BAr^F^_4_] **6**

To a flask charged with [Rh(PONOP-*t*Bu)Cl] (30.0 mg, 55.7 μmol) and Fc[BAr^F^_4_] (55.6 mg, 52.9 μmol) was added 1,2-C_6_H_4_F_2_ (2 mL). The resulting dark green solution was stirred at room temperature for 1 h before volatiles were removed in vacuo. The residue was washed with hexane (2 × 5 mL) and then dried in vacuo. Recrystallization from CH_2_Cl_2_/hexane at room temperature afforded the analytically pure product as purple crystals. Yield: 35.4 mg (25.3 μmol, 48%). Crystals grown in this way were suitable for analysis by X-ray diffraction.

^1^H NMR (400 MHz, CD_2_Cl_2_): δ 24.57 (vbr, fwhm = 600 Hz, 36H, *t*Bu), 7.51–7.60 (m, 8H, Ar^F^), 7.32 (br, 4H, Ar^F^), 1.45 (vbr, fwhm = 60 Hz, 2H, 3-py), −17.31 (vbr, fwhm = 110 Hz, 1H, 4-py).

^31^P{^1^H} NMR (162 MHz, CD_2_Cl_2_): no resonances observed between δ −600 and +600.

HR ESI-MS (positive ion, 4 kV): not sufficiently stable under the analysis conditions employed.

μ_eff_ (powder dispersed in *n*-eicosane): 2.22(2) μ_B_ (dc magnetic susceptibility), 2.20(2) μ_B_ (ac magnetic susceptibility).

Anal. Calcd for C_53_H_51_BClF_24_NO_2_P_2_Rh (1401.07 gmol^–1^): C, 45.44; H, 3.67; N, 1.00. Found: C, 45.59; H, 3.67; N, 1.03.

### 4.10 NMR Scale Reactions of [Rh(PONOP-*t*Bu)(CH_2_Cl)Cl][BAr^F^_4_] **5**

#### 4.10.1 Stability at Room Temperature in CD_2_Cl_2_

A solution of a 9:1 mixture of **5** and **6** (14.5 mg, 10.0 μmol/Rh) in CD_2_Cl_2_ (0.5 mL) was prepared within a J. Young’s valve NMR tube in the dark and monitored in situ using ^1^H and ^31^P NMR spectroscopy. No onward reaction was apparent after standing at room temperature for 48 h in the dark. The solution was exposed to light, and quantitative conversion of **5** into [Rh(PONOP-*t*Bu)(*κ*_Cl_–ClCD_2_Cl)][BAr^F^_4_] [d_2_-**A**; δ_31P_ 204.5 (d, ^1^*J*_RhP_ = 136)] was observed within 4 h at room temperature (orange solution). The concentration of **6** remained constant.

#### 4.10.2 Stability in the Presence of TEMPO in CD_2_Cl_2_

To a J. Young’s valve NMR tube charged with a 9:1 mixture of **5** and **6** (14.5 mg, 10.0 μmol/Rh) and TEMPO (1.6 mg, 10.2 μmol) was added CD_2_Cl_2_ (0.5 mL) at room temperature in the dark. The resulting solution was left to stand at room temperature for 24 h in the dark. No onward reaction was apparent from analysis in situ using ^1^H and ^31^P NMR spectroscopy. The solution was exposed to light, resulting in a gradual change in color from orange to deep red. Generation of a species assigned as TEMPO-CH_2_Cl [δ_1H_ 5.66 (s, OCH_2_Cl)]^27^ and quantitative conversion of **5** into [Rh(PONOP-*t*Bu)Cl][BAr^F^_4_] [**6**; δ_1H_ 23.96 (vbr, fwhm = 600 Hz, *t*Bu)] were observed within 4 h by ^1^H NMR spectroscopy.

### 4.11 NMR Scale Reactions of [Rh(PONOP-*t*Bu)Cl][BAr^F^_4_] **6**

#### 4.11.1 Stability at 50 °C in CD_2_Cl_2_

A 21 mM solution of **6** (14.5 mg, 10.3 μmol) in CD_2_Cl_2_ (0.5 mL) was prepared within J. Young’s valve NMR tube in the dark, heated at 50 °C in the dark, and periodically monitored in situ using ^1^H and ^31^P NMR spectroscopy at room temperature in the dark. No onward reaction was apparent after heating for 24 h (purple solution). The same outcome was observed when repeated in the presence of light.

#### 4.11.2 Reaction with Dihydroanthracene

A solution of **6** (14.0 mg, 10.0 μmol) and 9,10-dihydroanthracene (1.6 mg, 8.9 μmol) in CD_2_Cl_2_ (0.5 mL) within a J. Young’s valve NMR tube was heated for 2 weeks at 50 °C. Partial conversion (40%) of **6** into [Rh(PONOP-*t*Bu)(H)Cl][BAr^F^_4_] [**7**; δ_1H_ −26.23 (dt, ^1^*J*_RhH_ = 41.9, ^2^*J*_PH_ = 9.9, 1H, RhH), δ_31P_ 197.8 (d, ^1^*J*_RhP_ = 102)] with concomitant generation of anthracene [δ_1H_ 8.47 (s, 2H, CH); red/purple solution] was observed.

### 4.12 Preparation of [Rh(PONOP-*t*Bu)(*κ*_Cl_–ClCy)][BAr^F^_4_] **2**

To a flask charged with [Rh(PONOP-*t*Bu)(*κ*_Cl_–ClPh)][BAr^F^_4_] **1** (18.4 mg, 12.5 μmol) was added CyCl (0.5 mL). The resulting orange solution was left to stand at room temperature for 5 min before the volatiles were removed in vacuo to afford the analytically pure product as a yellow powder. Yield: 14.6 mg (9.8 μmol, 79%). Crystals suitable for analysis by X-ray diffraction were grown from CyCl/hexane at room temperature.

^1^H NMR (400 MHz, CyCl, selected peaks): δ 7.82–7.88 (m, 8H, Ar^F^), 7.78 (t, ^3^*J*_HH_ = 8.2, 1H, 4-py), 7.60 (br, 4H, Ar^F^), 6.78 (d, ^3^*J*_HH_ = 8.2, 2H, 3-py). No paramagnetic signals observed in the range −50 to +50 ppm.

^31^P{^1^H} NMR (162 MHz, CyCl): δ 204.5 (d, *J*_RhP_ = 138).

Anal. Calcd for C_59_H_62_BClF_24_NO_2_P_2_Rh·C_6_H_11_Cl (1602.83 gmol^–1^): C, 48.71; H, 4.59; N, 0.87. Found: C, 49.09; H, 4.53; N, 0.96.

### 4.13 NMR Scale Reactions of [Rh(PONOP-*t*Bu)(*κ*_Cl_–ClCy)][BAr^F^_4_] **2**

Reactions were performed within J. Young’s valve NMR tubes using 20 mM solutions of **2** (14.8 mg, 10.0 μmol) in CyCl (0.5 mL) and monitored in situ using ^1^H and ^31^P NMR spectroscopy.

#### 4.13.1 Stability at Room Temperature in CyCl

Standing at room temperature for 24 h in the dark resulted in partial conversation (10%) of **2** into [Rh(PONOP-*t*Bu)(H)Cl][BAr^F^_4_] [**7**; δ_1H_ −26.12 (dt, ^1^*J*_RhH_ = 42.3, ^2^*J*_PH_ = 10.6, RhH), δ_31P_ 197.1 (d, ^1^*J*_RhP_ = 100)]. The same outcome was observed when repeated in the presence of light.

#### 4.13.2 Stability at 50 °C in CyCl

Heating **2** in CyCl at 50 °C for 24 h in the dark resulted in generation of 1 equiv of cyclohexene [δ_1H_ 5.73 (s, CH=CH)] and quantitative formation of [Rh(PONOP-*t*Bu)(H)Cl][BAr^F^_4_] [**7**; δ_1H_ −26.15 (dt, ^1^*J*_RhH_ = 42.2, ^2^*J*_PH_ = 10.3, 1H, RhH), δ_31P_ 197.1 (d, ^1^*J*_RhP_ = 102); yellow solution]. The same outcome was observed when repeated in the presence of light.

### 4.14 Preparation of [Rh(PONOP-*t*Bu)(H)Cl][BAr^F^_4_] **7**

To a flask charged with [Rh(PONOP-*t*Bu)(*κ*_Cl_–ClPh)][BAr^F^_4_] **1** (29.6 mg, 20.0 μmol) was added *t*BuCl (1 mL) in the dark. The solution was left to stand at room temperature for 5 min before volatiles were removed in vacuo. Recrystallization from CH_2_Cl_2_/hexane at room temperature in the dark afforded the analytically pure product as yellow crystals.^29^ Yield: 24.3 mg (17.3 μmol, 87%). Crystals grown in this way were suitable for analysis by X-ray diffraction.

^1^H NMR (500 MHz, CD_2_Cl_2_): δ 7.96 (t, ^3^*J*_HH_ = 8.2, 1H, 4-py), 7.68–7.74 (m, 8H, Ar^F^), 7.55 (br, 4H, Ar^F^), 7.01 (d, ^3^*J*_HH_ = 8.3, 2H, 3-py), 1.50 (vt, *J*_PH_ = 16.3, 18H, *t*Bu), 1.46 (vt, *J*_PH_ = 16.8, 18H, *t*Bu), −26.25 (dt, ^1^*J*_RhH_ = 41.9, ^2^*J*_PH_ = 10.1, 1H, RhH).

^13^C{^1^H} NMR (126 MHz, CD_2_Cl_2_): δ 165.0 (vt, *J*_PC_ = 4, 2-py), 162.3 (q, ^1^*J*_BC_ = 50, Ar^F^), 147.3 (s, 4-py), 135.3 (s, Ar^F^), 129.4 (qq, ^2^*J*_FC_ = 32, ^3^*J*_CB_ = 3, Ar^F^), 125.1 (q, ^1^*J*_FC_ = 272, Ar^F^), 118.0 (sept, ^3^*J*_FC_ = 4, Ar^F^), 105.7 (vt, *J*_PC_ = 4, py), 43.1 (vt, *J*_PC_ = 12, *t*Bu{C}), 41.0 (vtd, *J*_PC_ = 14, ^2^*J*_RhC_ = 2, *t*Bu{C}), 27.33 (vt, *J*_PC_ = 6, *t*Bu{CH_3_}), 27.31 (vt, *J*_PC_ = 6, *t*Bu{CH_3_}).

^31^P{^1^H} NMR (162 MHz, CD_2_Cl_2_): δ 197.7 (d, ^1^*J*_RhP_ = 100).

HR-ESI-MS (positive ion, 4 kV): not sufficiently stable under the analysis conditions employed.

Anal. Calcd for C_53_H_52_BClF_24_NO_2_P_2_Rh (1402.08 gmol^–1^): C, 45.40; H, 3.74; N, 1.00. Found: C, 45.54; H, 3.80; N, 1.04.

### 4.15 NMR Scale Reactions of [Rh(PONOP-*t*Bu)(H)Cl][BAr^F^_4_] **7**

#### 4.15.1 Stability at Room Temperature in CD_2_Cl_2_

20 mM solutions of **7** (14.0 mg, 10.0 μmol) in CD_2_Cl_2_ (0.5 mL) were prepared within J. Young’s valve NMR tubes in the presence and absence of light and thereafter monitored in situ using ^1^H and ^31^P NMR spectroscopy. No significant onward reaction of **7** was apparent upon standing at room temperature for 72 h in the dark, but partial decomposition (2%) into [Rh(PONOP-*t*Bu)Cl][BAr^F^_4_] [**6**; δ_1H_ 24.2 (vbr, fwhm = 500 Hz, *t*Bu)] was observed in the presence of light under the same conditions (yellow solution).

#### 4.15.2 Reaction with TEMPO in CD_2_Cl_2_

Solutions of **7** (20 mM) and TEMPO (0.5, 1.0 and 2.0 equiv) in CD_2_Cl_2_ were prepared in J. Young’s valve NMR tubes by dissolution of **7** (14.8 mg, 10.0 μmol) in varying ratios of a 50 mM standard solution of TEMPO in CD_2_Cl_2_ and CD_2_Cl_2_ (totalling 0.5 mL). Analysis by ^1^H NMR spectroscopy at 298 K indicated hydrogen atom abstraction and establishment of a dynamic equilibrium involving hydrogen atom transfer between **6** and **7** on the time scale of the NMR experiment, most notably evidenced by the presence of a board 36H resonance at δ 13.2 (∼ equally weighted average of the *t*Bu signals of **6** and **7**), which was sharper with higher concentrations of added TEMPO. A comparatively sharp integral 1H resonance at δ 3.95 is consistent with the formation of TEMPOH. No dynamic exchange was observed for a 1:1 mixture of **6** and **7** ([Rh] = 20 mM) in CD_2_Cl_2_, and a control experiment indicated no direct reaction between **6** (20 mM) and TEMPO (1 equiv) in CD_2_Cl_2_.

### 4.16 Crystallographic Details

Data were collected on a Rigaku Oxford Diffraction SuperNova AtlasS2 CCD diffractometer using graphite monochromated Mo Kα (λ = 0.71073 Å) or CuKα (λ = 1.54184 Å) radiation and an Oxford Cryosystems *N*-HeliX low-temperature device [150(2) K]. Data were collected and reduced using CrysAlisPro and refined using SHELXT^35^ through the Olex2 interface.^36^ The disorder evident in cationic components of **1** (Ph), **2** (Cy), and **7** (3× *t*Bu) was treated by splitting or modeling the affected moiety over two sites, restraining its geometry and restraining the associated thermal parameters. The Ph group in **1** was also constrained to an ideal geometry. Partial co-crystallization of **6** with **5** was treated by freely refining the occupancy of the CH_2_Cl ligand in **5** [0.892(5)]. Full details about the collection, solution, and refinement are documented in CIF format, which have been deposited with the Cambridge Crystallographic Data Centre under CCDC 2195204 (**1**), 2195205 (**2**), 2195206 (**4**), 2195207 (**5**), 2195208 (**6**), and 2195209 (**7**).

## Supporting Information Available

NMR and ESI-MS spectra of new compounds and selected reactions, cyclic voltammograms for the oxidation of [Rh(PONOP-*t*Bu)Cl], and ac magnetic susceptibility measurements for **6** (PDF)
