# Supplementary material for: Oxidative Addition of C–Cl Bonds to a Rh(PONOP) Pincer Complex
Source: Organometallics. 2022 Oct 31;41(23):3557–67. doi: 10.1021/acs.organomet.2c00400 (PMC9749114; doi:10.1021/acs.organomet.2c00400)
Supplement: Supplementary file 1 — om2c00400_si_001.pdf [file om2c00400_si_001.pdf]

Supporting information for:

Oxidative addition of C–Cl bonds to a Rh(PONOP) pincer complex

Alexandra Longcake,[a] Martin R. Lees,[b] Mark S. Senn,[a] Adrian B. Chaplin[a,*]

[a] *Department of Chemistry, University of Warwick, Gibbet Hill Road, Coventry CV4 7AL, UK*

Email: a.b.chaplin@warwick.ac.uk

[b] *Department of Physics, University of Warwick, Gibbet Hill Road, Coventry CV4 7AL, UK*

Table of contents

1 NMR scale reaction of [{Rh(PONOP-*t*Bu)}$_2$(μ-η^2:η^2-COD)][BAr$^F{}_4$]$_2$ with PhCl S2

2 Characterisation of [Rh(PONOP-*t*Bu)(κ_{Cl}–ClPh)][BAr$^F{}_4$] 1 .... S3

3 NMR scale reactions of [Rh(PONOP-*t*Bu)(κ_{Cl}–ClPh)][BAr$^F{}_4$] 1 .... S4

3.1 Stability at room temperature in PhCl S4

3.2 Stability at 125 °C in PhCl S5

3.3 Stability at 125 °C in PhCl in the presence of TEMPO. S7

3.4 Stability at room temperature in CD_2Cl_2 .... S8

3.5 Stability at room temperature in CyCl S9

3.6 Stability at room temperature in tBuCl S10

4 Characterisation of [Rh(PONOP-*t*Bu)(Ph)Cl][BAr$^F{}_4$] 4 .... S11

5 NMR scale reactions of [Rh(PONOP-*t*Bu)(Ph)Cl][BAr$^F{}_4$] 4 .... S13

5.1 Stability at room temperature in CD_2Cl_2 .... S13

5.2 Stability in presence of TEMPO in CD_2Cl_2 .... S14

6 Characterisation of [Rh(PONOP-*t*Bu)(κ_{Cl}–ClCH$_2$Cl)][BAr$^F{}_4$] A S15

7 NMR scale reactions of [Rh(PONOP-*t*Bu)(κ_{Cl}–ClCX$_2$Cl)][BAr$^F{}_4$] (X = H, A; D, d$_2$-A) S16

7.1 Stability at room temperature in CD_2Cl_2 .... S16

7.2 Stability at 50 °C in CD_2Cl_2 .... S18

7.3 Solid-state stability S20

*7.4 Characterisation of [Rh(PONOP-tBu)(CH$_2$Cl)Cl][BAr$^F{}_4$] **5** S20*

8 Cyclic voltammograms for the oxidation of [Rh(PONOP-*t*Bu)Cl] S23

9 Characterisation of [Rh(PONOP-*t*Bu)Cl][BAr$^F{}_4$] 6 .... S24

10 NMR scale reactions of [Rh(PONOP-*t*Bu)(CH$_2$Cl)Cl][BAr$^F{}_4$] 5 .... S26

10.1 Stability at room temperature in CD_2Cl_2 .... S26

10.2 Stability in the presence of TEMPO in CD_2Cl_2 .... S28

11 NMR scale reactions of [Rh(PONOP-*t*Bu)Cl][BAr$^F{}_4$] 6 .... S30

11.1 Stability at 50 °C in CD_2Cl_2 .... S30

11.2 Reaction with dihydroanthracene S31

12 Characterisation of [Rh(PONOP-*t*Bu)(κ_{Cl}–ClCy)][BAr$^F{}_4$] 2 .... S32

13 NMR scale reactions of [Rh(PONOP-*t*Bu)(κ_{Cl}–ClCy)][BAr$^F{}_4$] 2 .... S34

13.1 Stability at room temperature in CyCl S34

13.2 Stability at 50 °C in CyCl S35

14 Characterisation of [Rh(PONOP-*t*Bu)(H)Cl][BAr$^F{}_4$] 7 .... S36

15 NMR scale reactions of [Rh(PONOP-*t*Bu)(H)Cl][BAr$^F{}_4$] 7 .... S38

15.1 Stability at room temperature in CD_2Cl_2 .... S38

15.2 Reaction with TEMPO in CD_2Cl_2 .... S40

1 NMR scale reaction of [{Rh(PONOP-*t*Bu)}$_2$(μ-η^2:η^2-COD)][BAr^F$_4$]$_2$ with PhCl

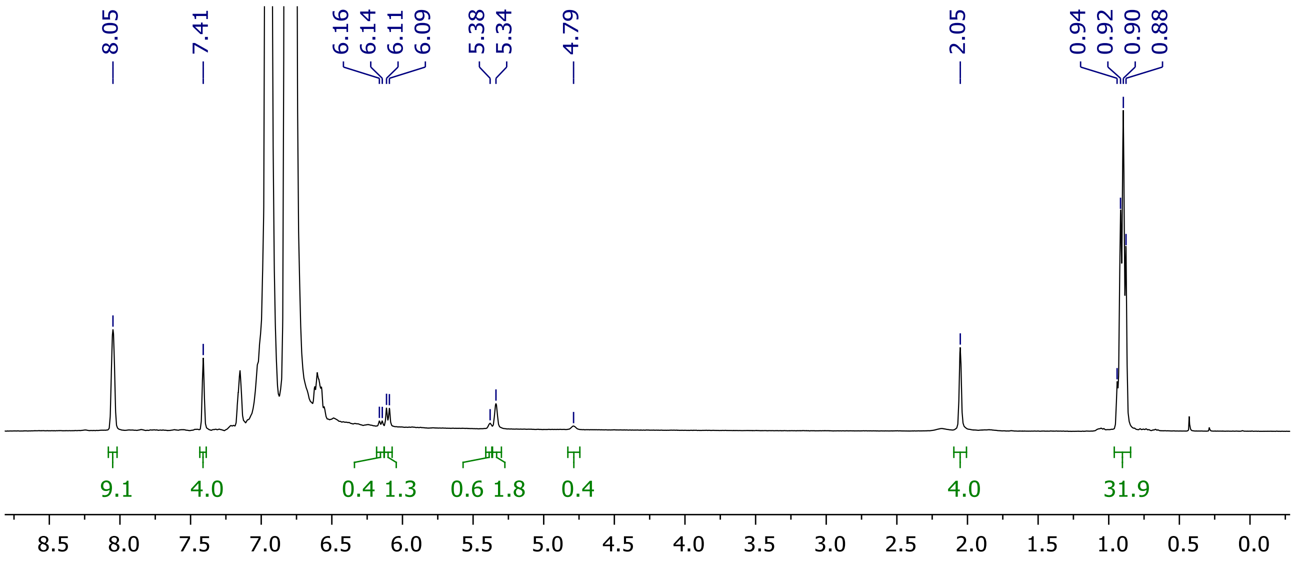

Figure S1. ^{1}H NMR spectrum recorded after 6 h at room temperature (400 MHz, PhCl).

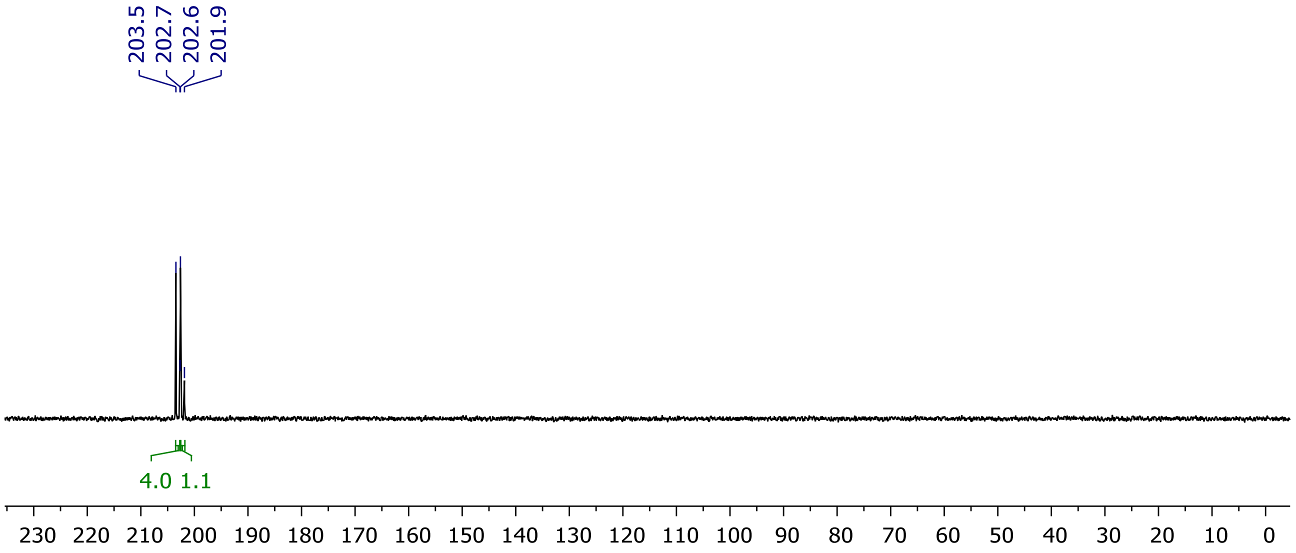

Figure S2. ^{31}P{^{1}H} NMR spectrum recorded after 6 h at room temperature (162 MHz, PhCl).

2 Characterisation of [Rh(PONOP-*t*Bu)(κ_{Cl}–ClPh)][$BAr^F{}_4$] 1

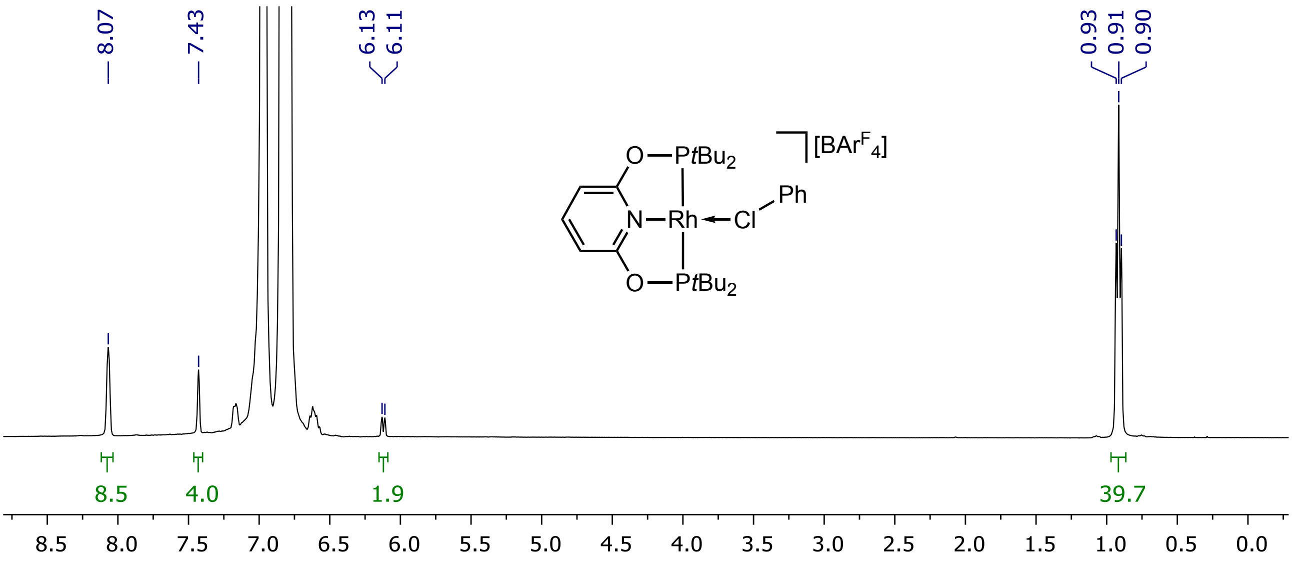

Figure S3. ^{1}H NMR spectrum of **1** (400 MHz, PhCl).

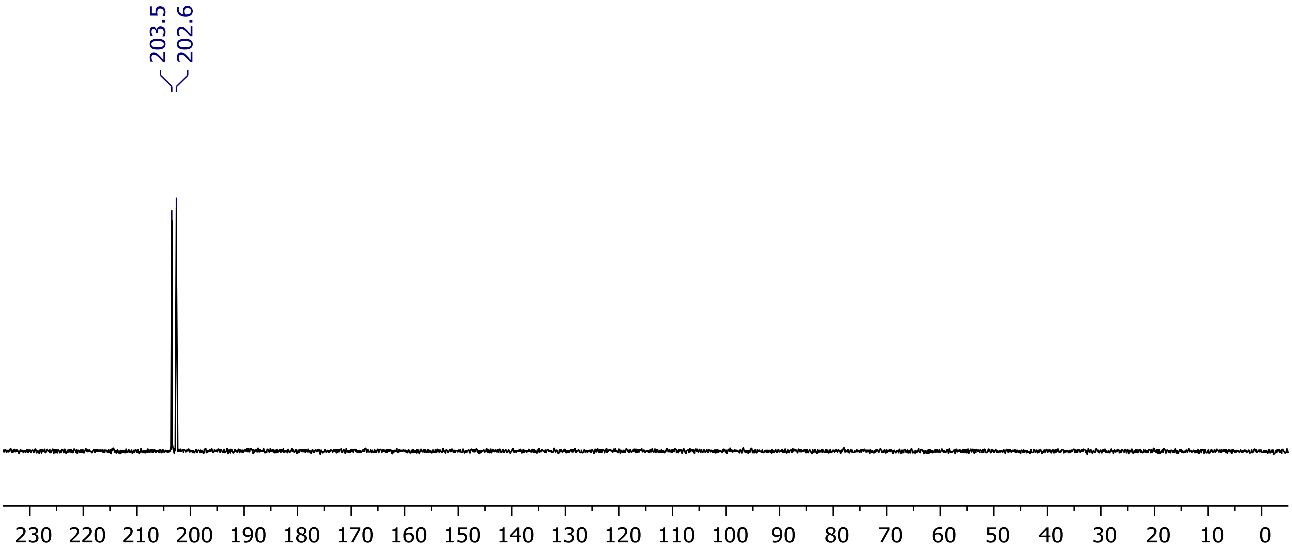

Figure S4. ^{31}P{^{1}H} NMR spectrum of **1** (162 MHz, PhCl).

3 NMR scale reactions of [Rh(PONOP-*t*Bu)(κ_{Cl}–ClPh)][BAr$^F_4$] 1

3.1 Stability at room temperature in PhCl

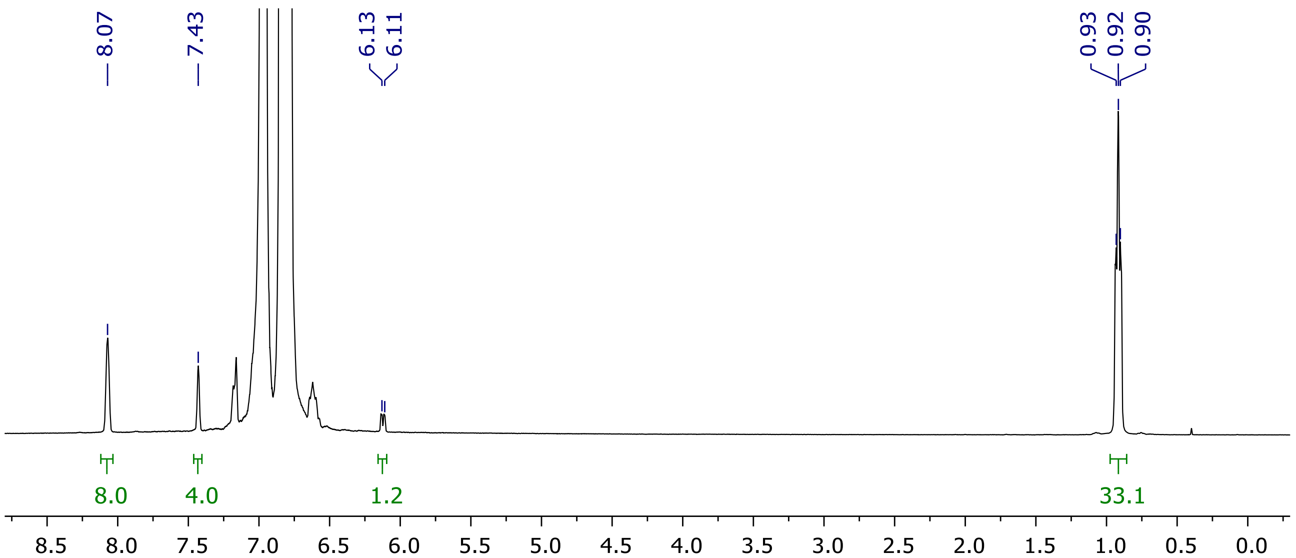

Figure S5. ^{1}H NMR spectrum recorded after 72 h at room temperature in the presence of light (400 MHz, PhCl). Similar data observed in the dark.

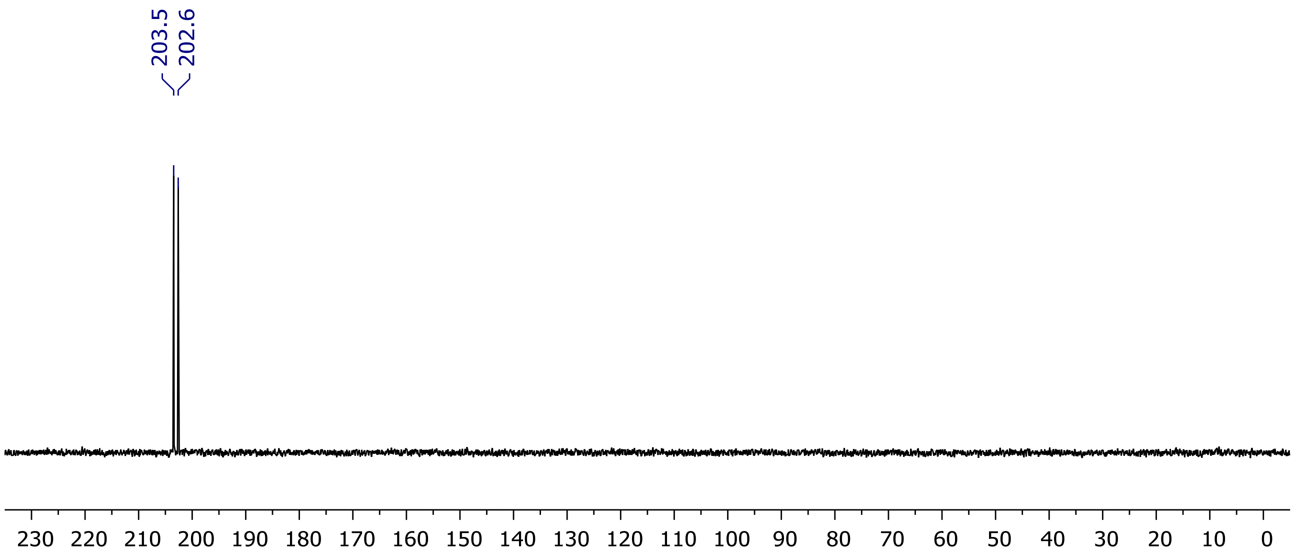

Figure S6. ^{31}P{^{1}H} NMR spectrum recorded after 72 h at room temperature in the presence of light (162 MHz, PhCl). Similar data observed in the dark.

3.2 Stability at 125 °C in PhCl

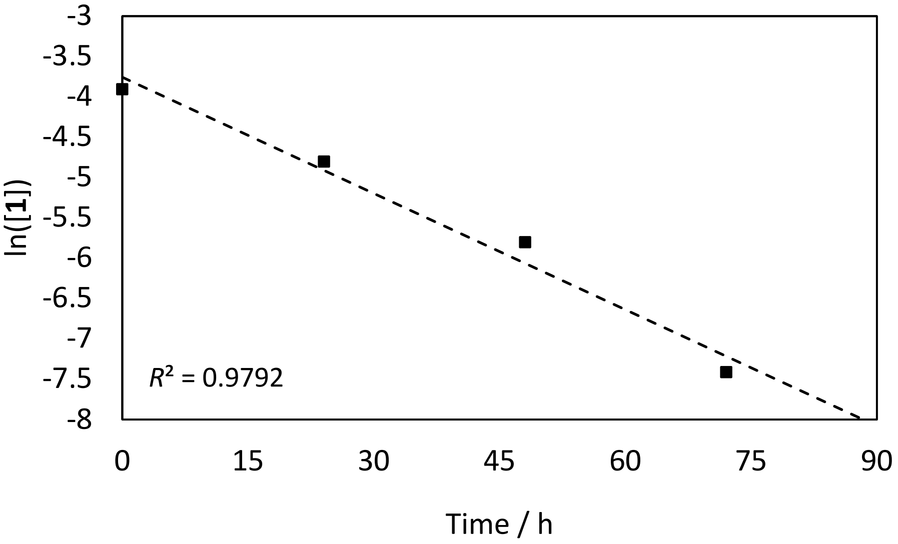

Figure S7. Plot demonstrating pseudo first order consumption of **1** when heated at 125 °C in the dark. Concentrations determined by integration of ^{1}H NMR data; k_{obs} = 0.048 h^{-1} ($t_{1/2}$ = 14 h).

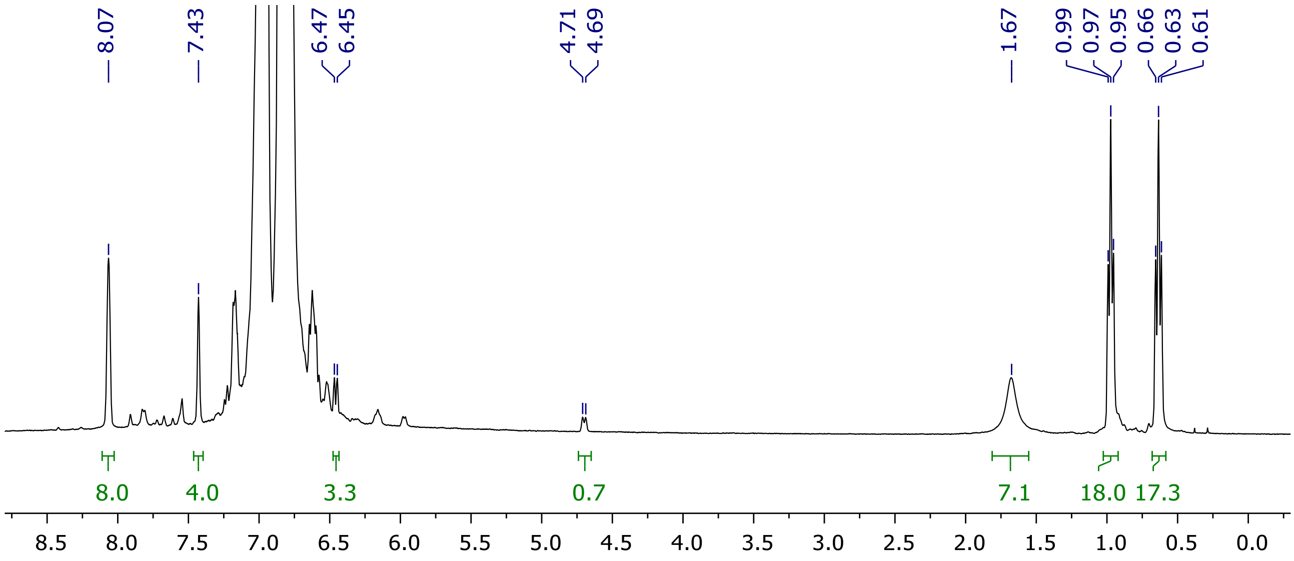

Figure S8. ^{1}H NMR spectrum recorded after 96 h at 125 °C in the dark (400 MHz, PhCl). Similar data observed when exposed to light.

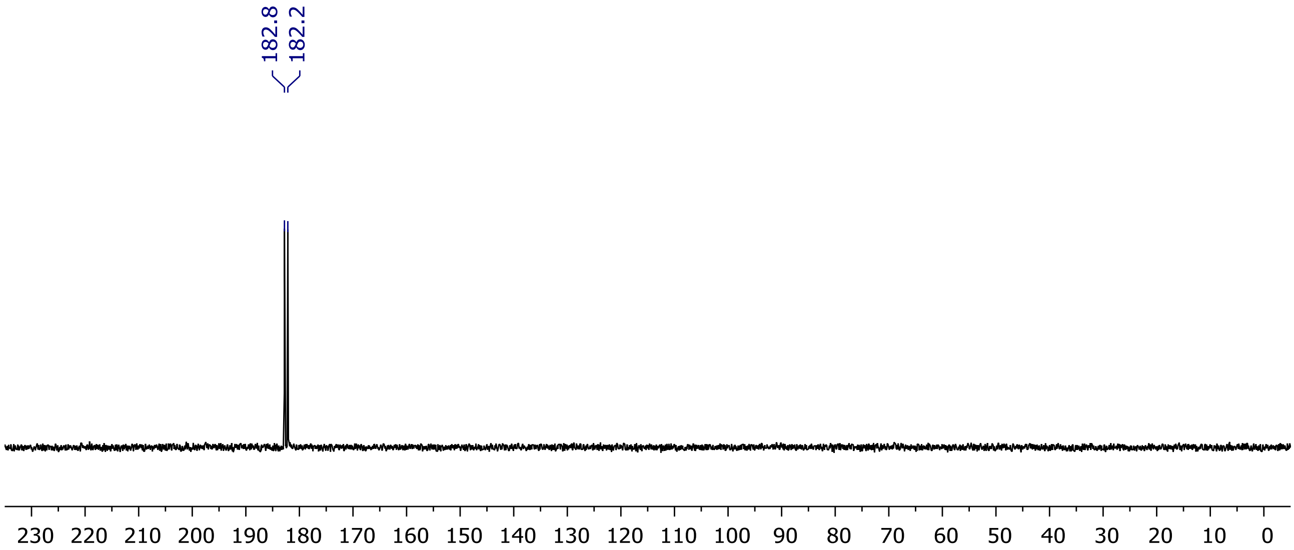

Figure S9. $^{31}P\{^1H\}$ NMR spectrum recorded after 96 h at 125 °C in the dark (162 MHz, PhCl). Similar data observed when exposed to light.

3.3 Stability at 125 °C in PhCl in the presence of TEMPO.

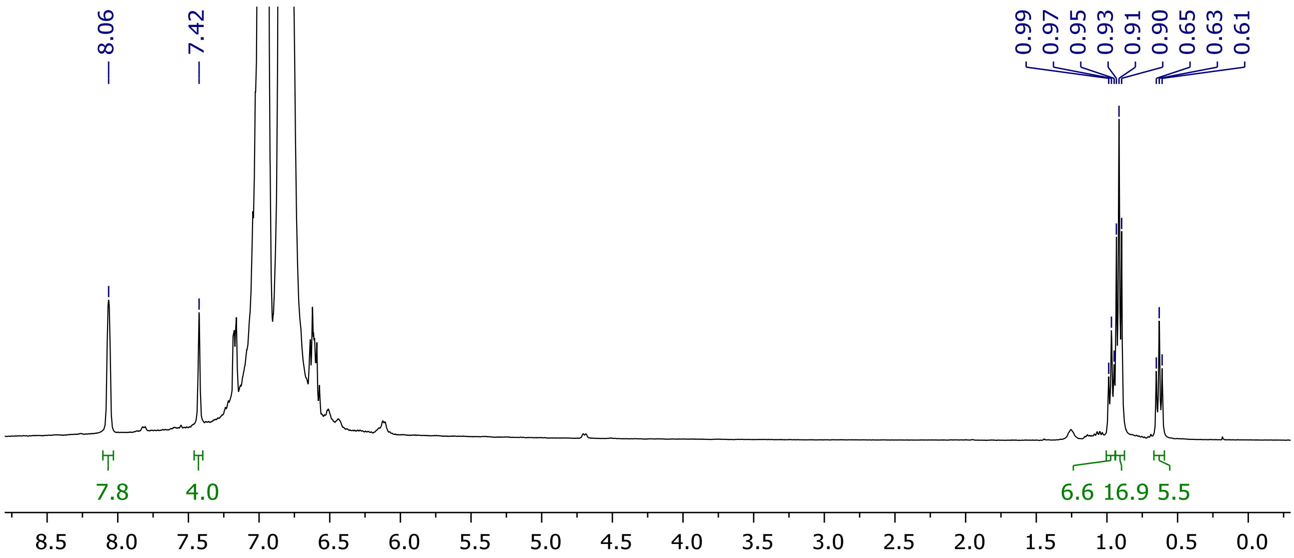

Figure S10. ^{1}H NMR spectrum recorded after 24 h at 125 °C in the dark (400 MHz, PhCl).

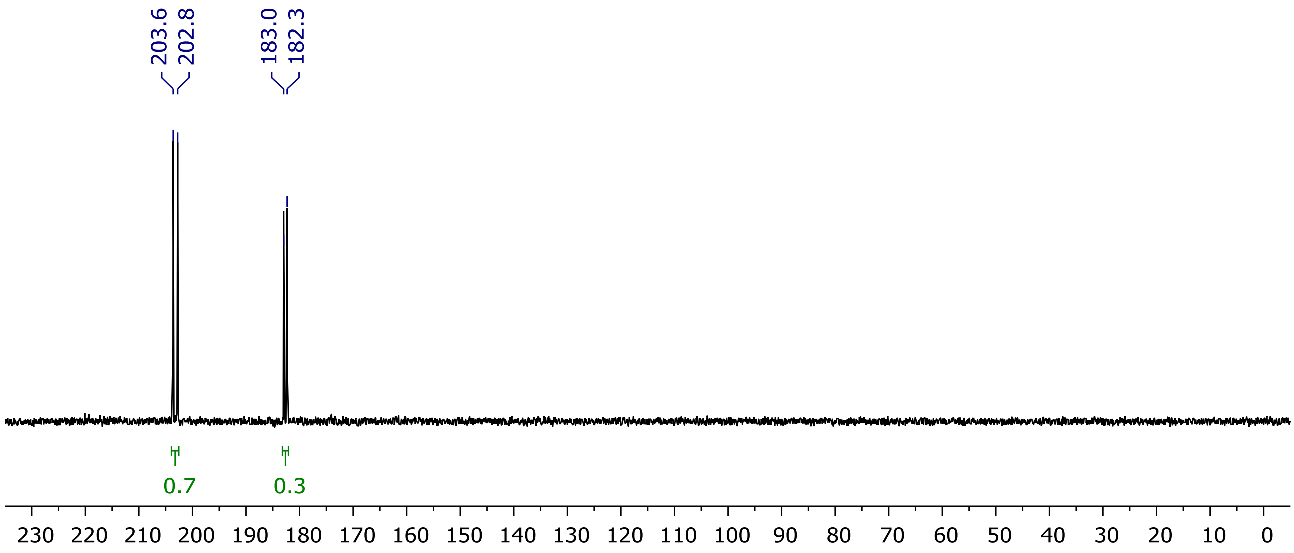

Figure S11. ^{31}P{^{1}H} NMR spectrum recorded after 24 h at 125 C in the dark (162 MHz, PhCl).

3.4 Stability at room temperature in CD_2Cl_2

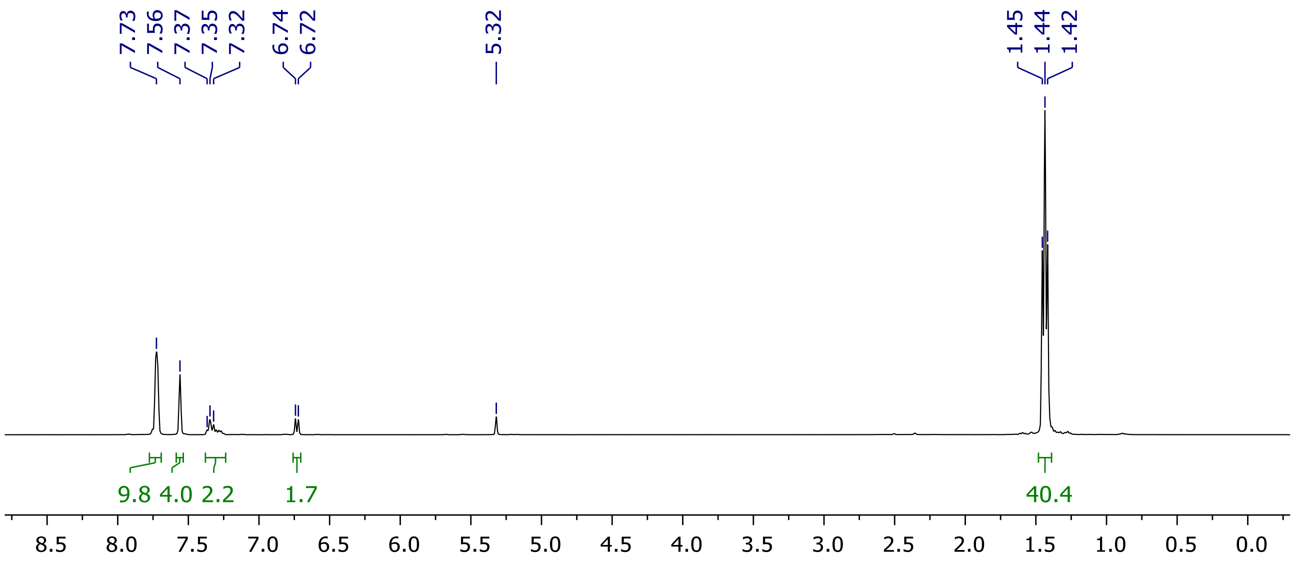

Figure S12. ^{1}H NMR spectrum of recorded within 5 min at room temperature (400 MHz, CD_2Cl_2).

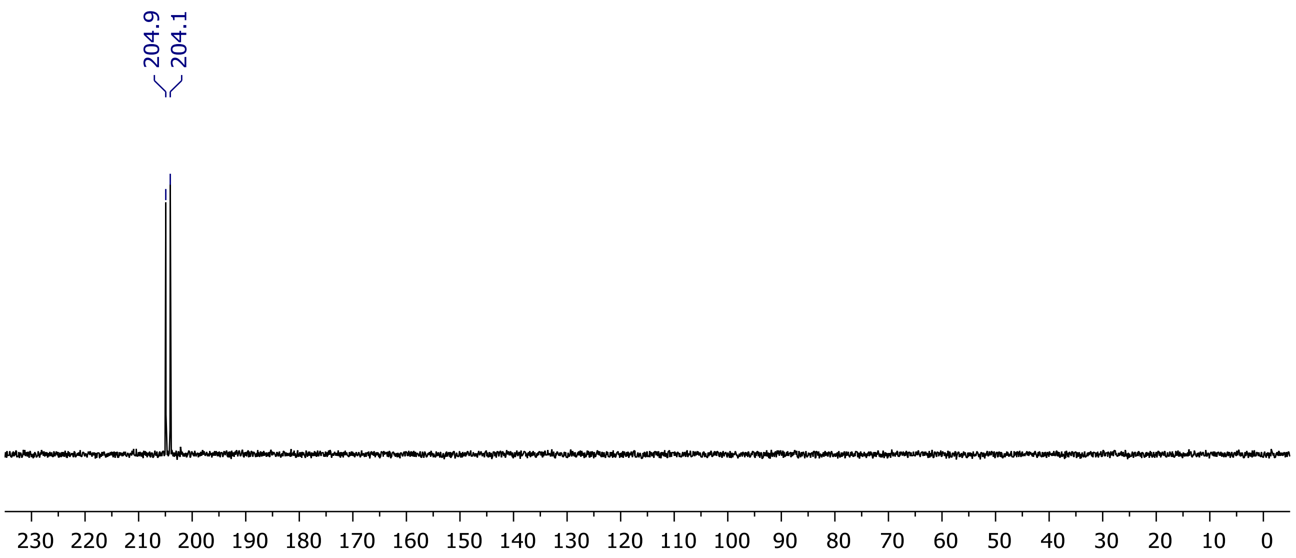

Figure S13. $^{31}P\{^{1}H\}$ NMR spectrum recorded within 5 min at room temperature (162 MHz, CD_2Cl_2).

3.5 Stability at room temperature in CyCl

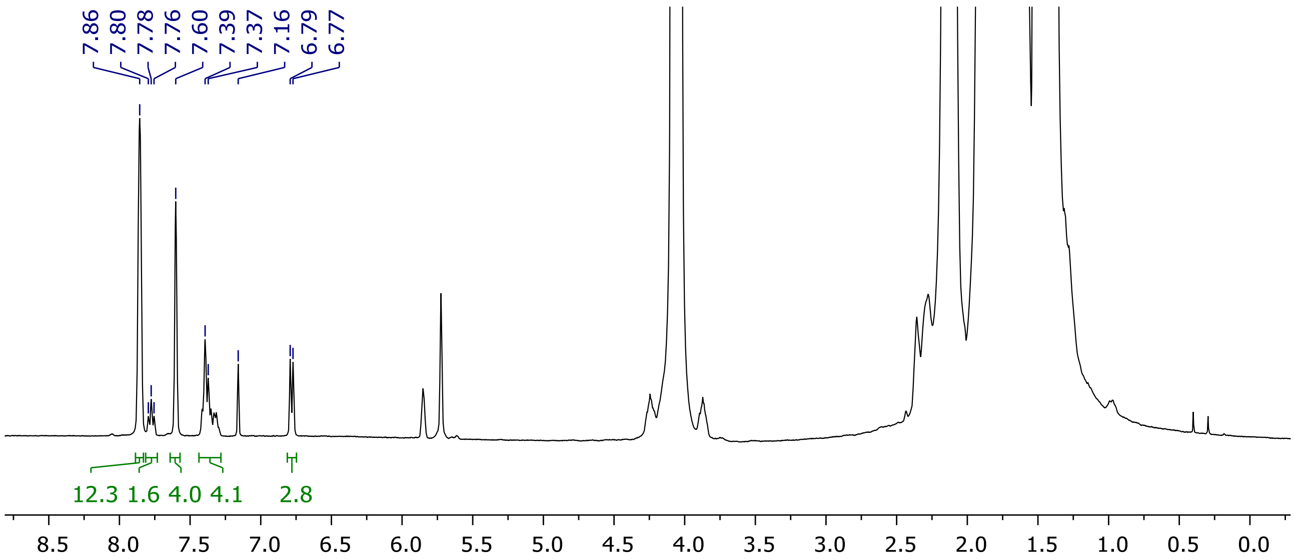

Figure S14. ^{1}H NMR spectrum recorded within 5 min at room temperature (400 MHz, CyCl).

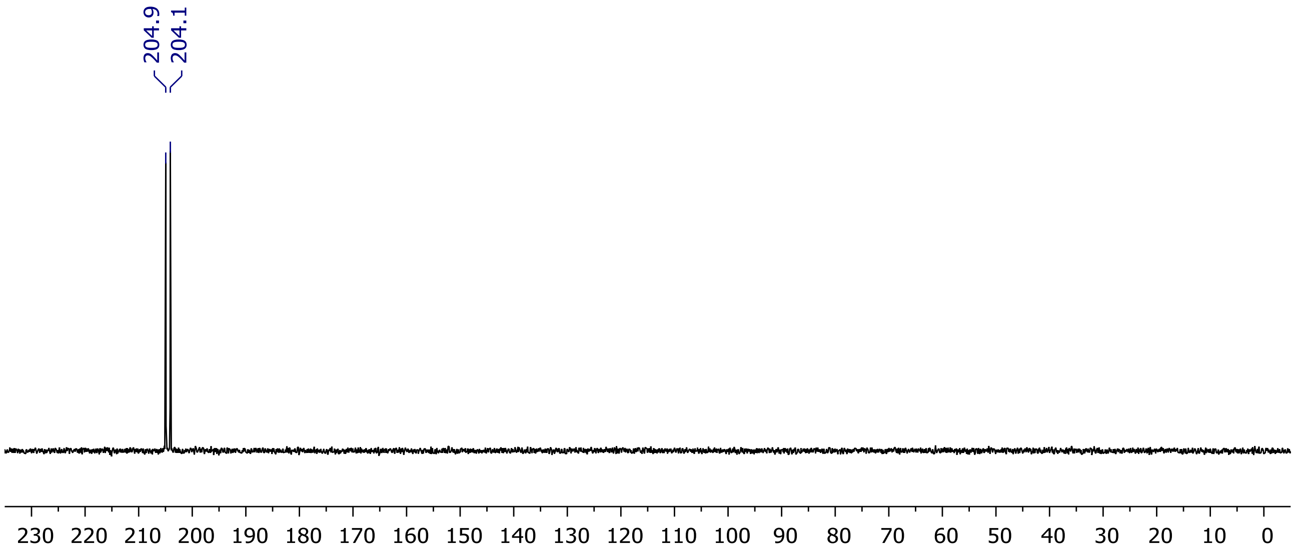

Figure S15. $^{31}P\{^{1}H\}$ NMR spectrum recorded within 5 min at room temperature (162 MHz, CyCl).

3.6 Stability at room temperature in *t*BuCl

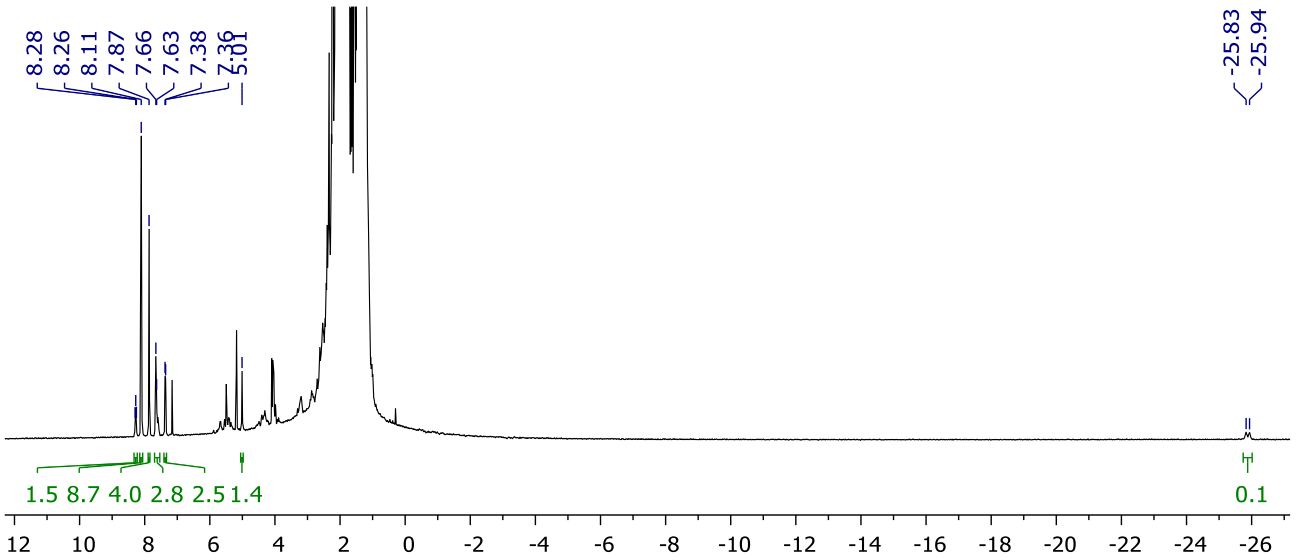

Figure S16. ^{1}H NMR spectrum recorded within 5 min at room temperature (400 MHz, *t*BuCl).

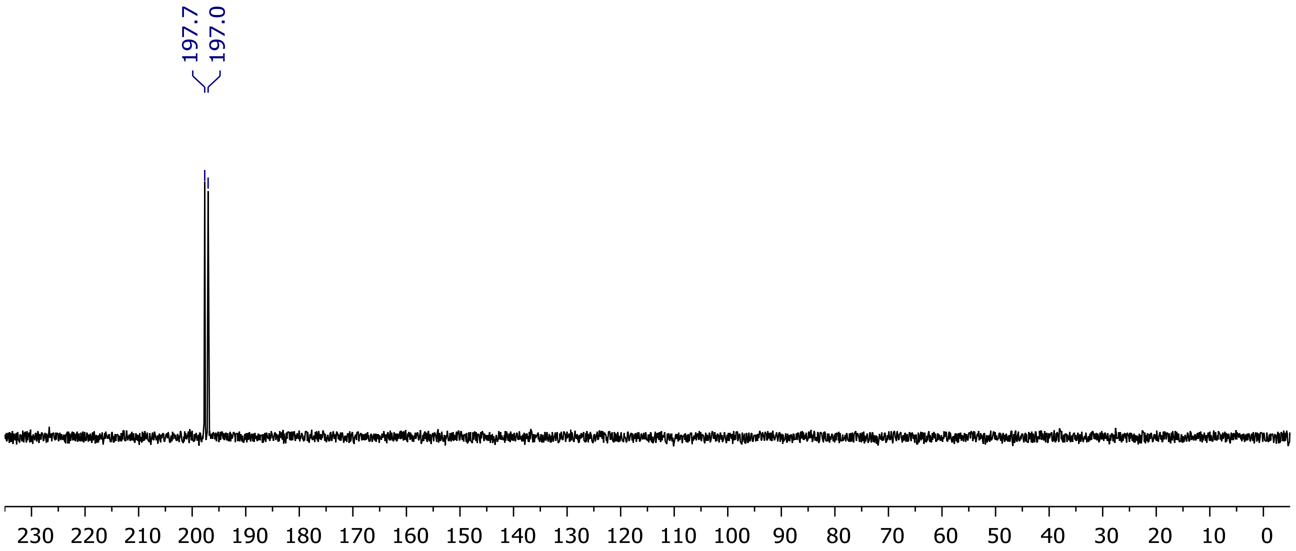

Figure S17. $^{31}P\{^{1}H\}$ NMR spectrum recorded within 5 min at room temperature (162 MHz, *t*BuCl).

4 Characterisation of [Rh(PONOP-*t*Bu)(Ph)Cl][BAr$^F_4$] 4

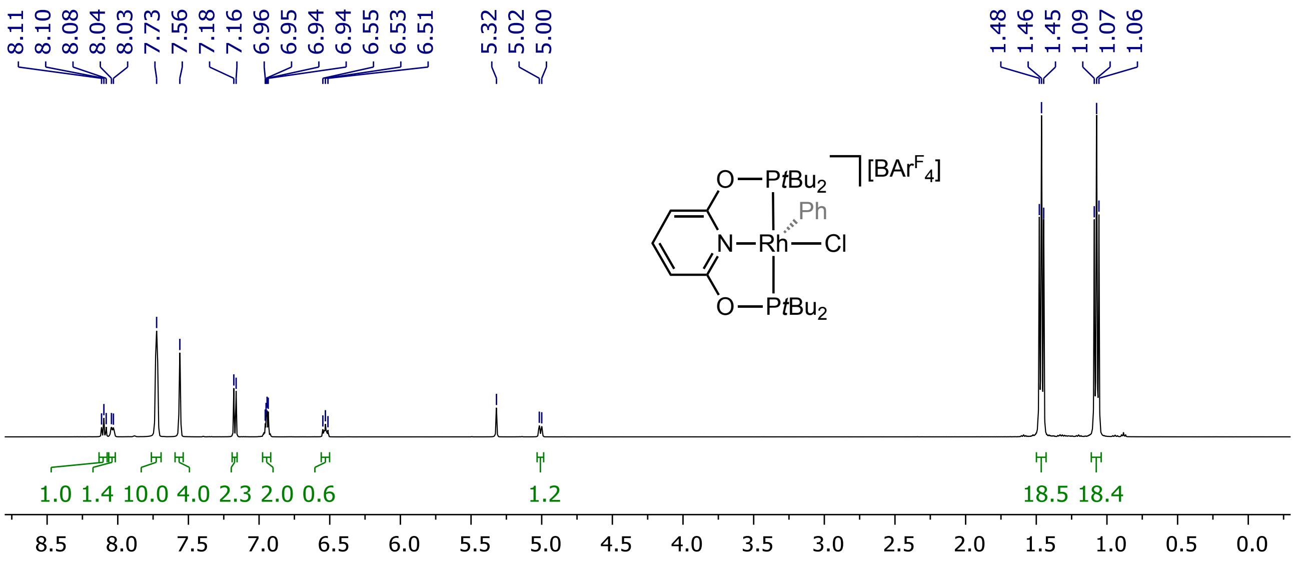

Figure S18. ^{1}H NMR spectrum of **4** (500 MHz, CD_2Cl_2).

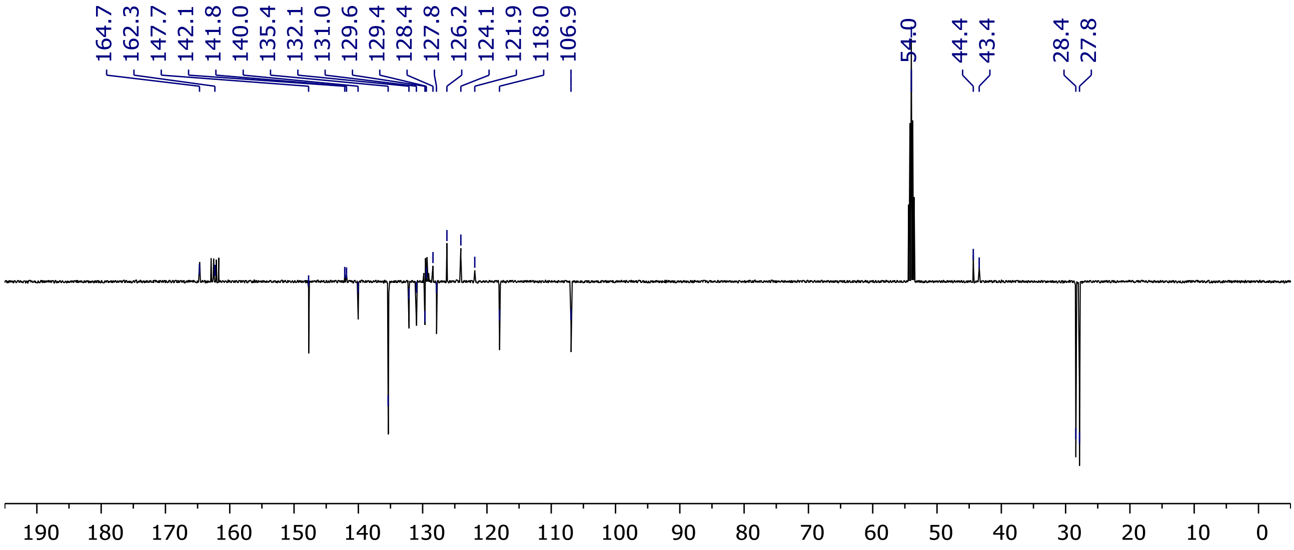

Figure S19. ^{13}C{^{1}H} APT NMR spectrum of **4** (162 MHz, CD_2Cl_2).

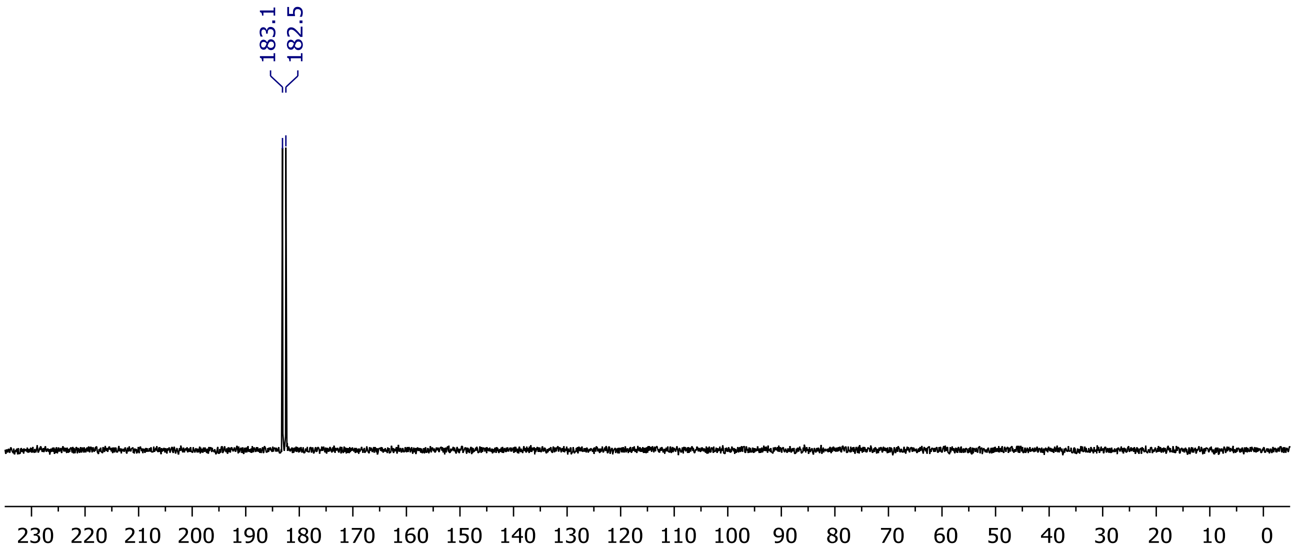

Figure S20. $^{31}P\{^{1}H\}$ NMR spectrum of **4** (162 MHz, CD_2Cl_2).

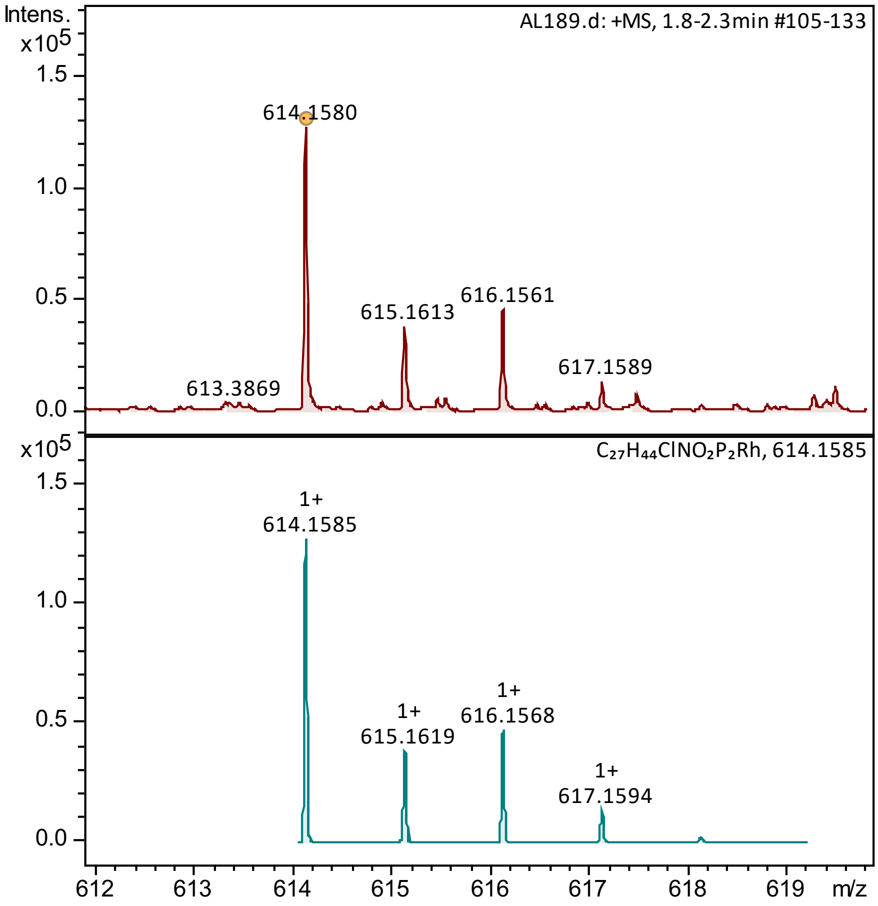

Figure S21. HR ESI-MS of **4**.

5 NMR scale reactions of [Rh(PONOP-*t*Bu)(Ph)Cl][BAr^F$_4$] 4

5.1 Stability at room temperature in CD_2Cl_2

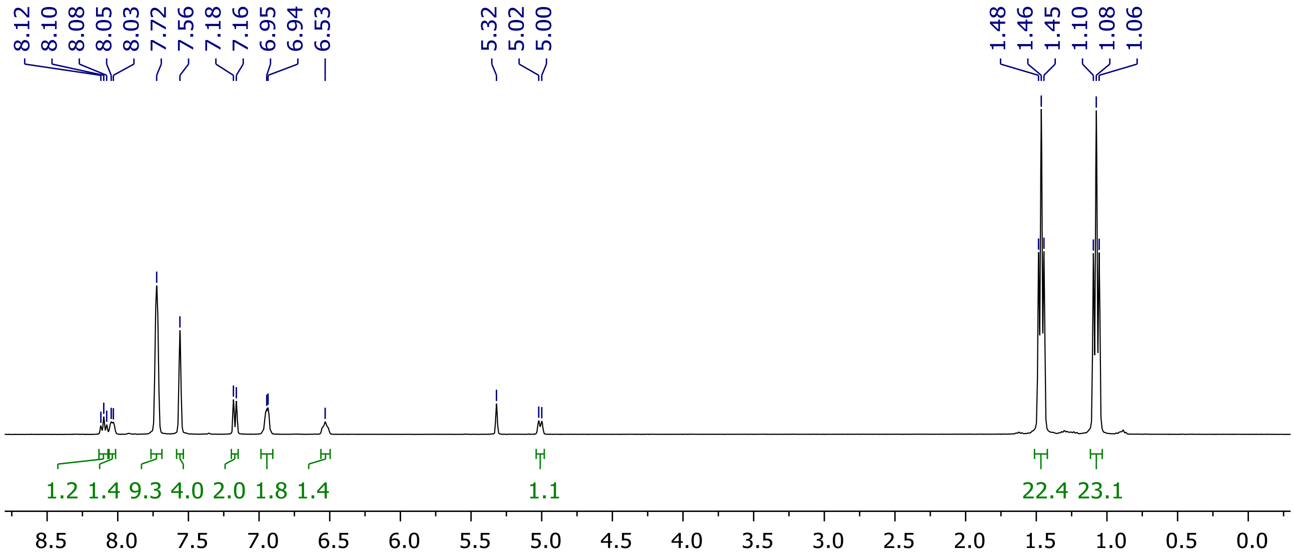

Figure S22. ^{1}H NMR spectrum recorded after 24 h at room temperature in dark, followed by 24 h at room temperature exposed to light (400 MHz, CD_2Cl_2).

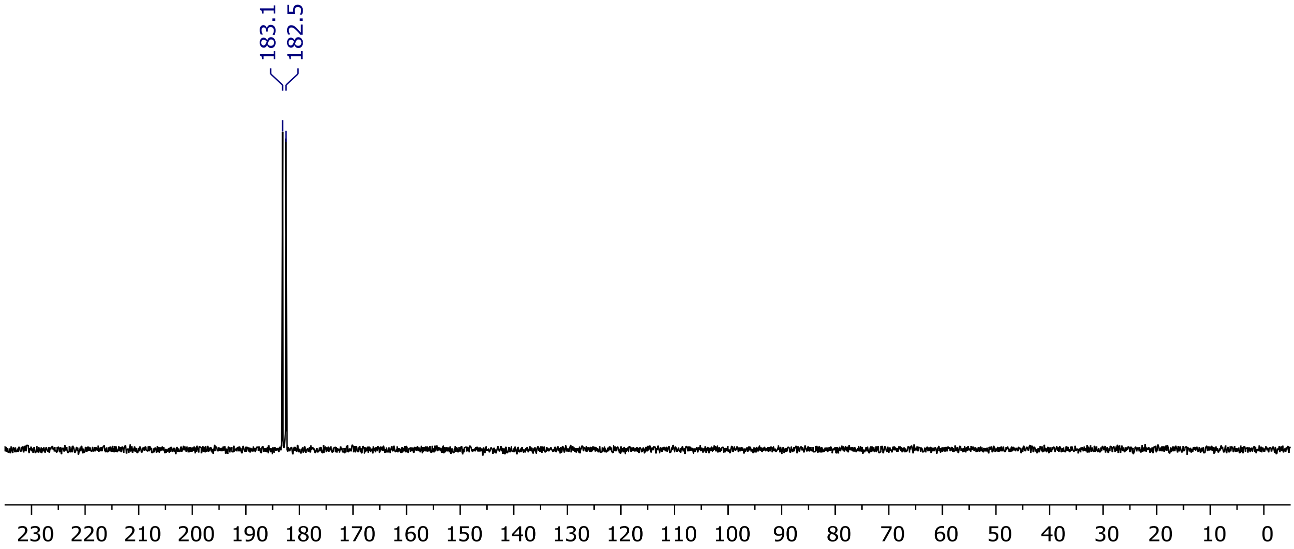

Figure S23. ^{31}P{^{1}H} NMR recorded after 24 h at room temperature in dark, followed by 24 h at room temperature exposed to light (162 MHz, CD_2Cl_2).

5.2 Stability in presence of TEMPO in CD_2Cl_2

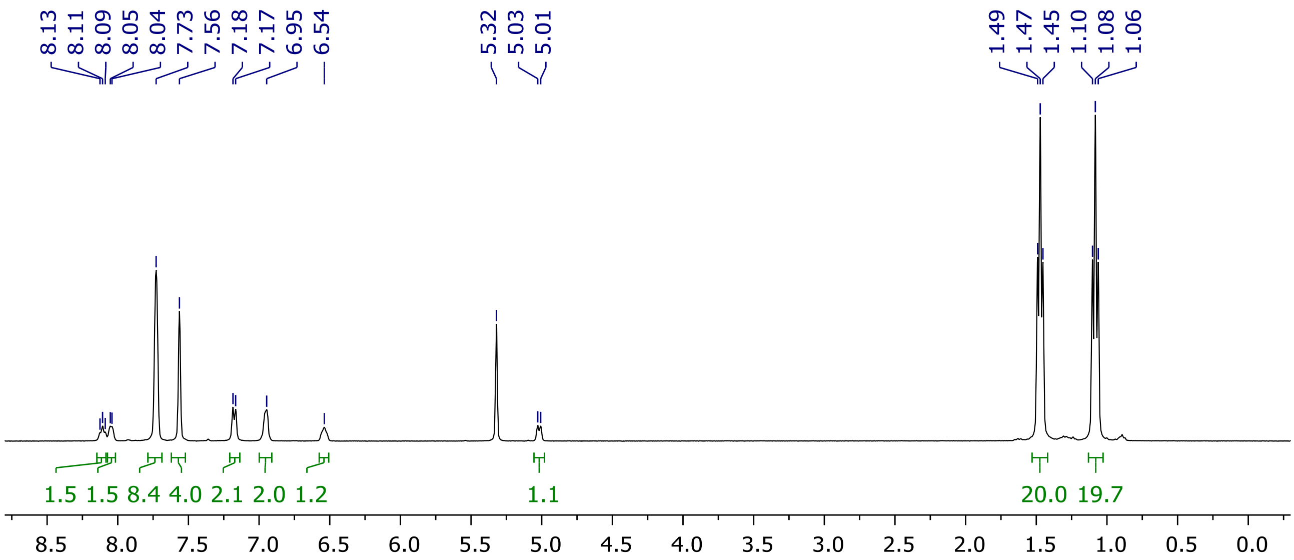

Figure S24. ^{1}H NMR spectrum recorded after 24 h at room temperature in dark, followed by 24 h at room temperature exposed to light (400 MHz, CD_2Cl_2).

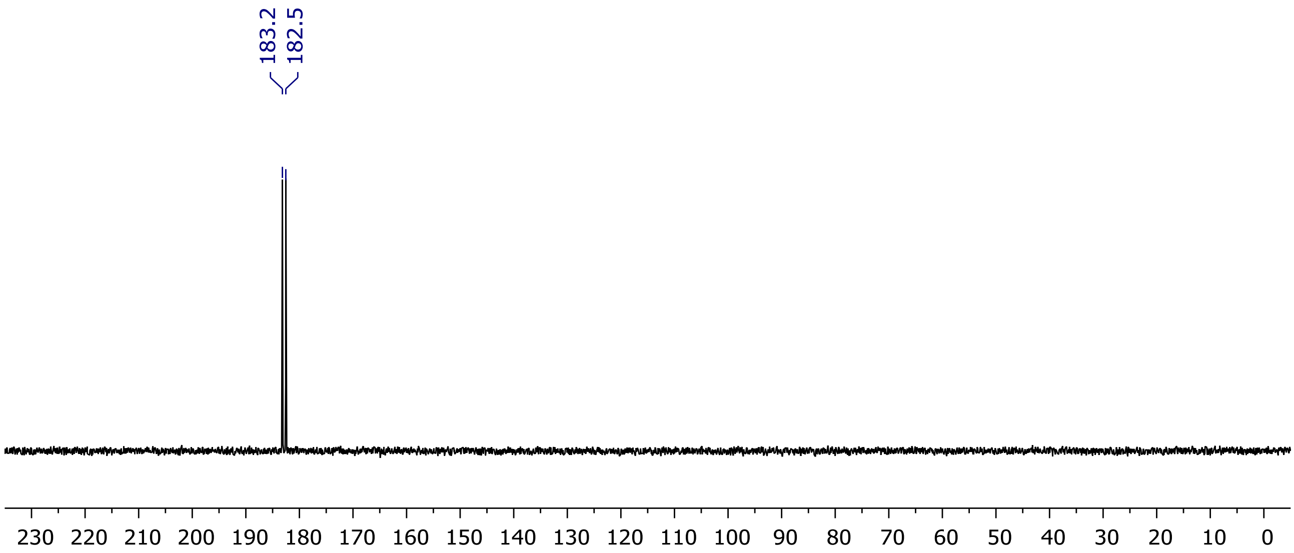

Figure S25. $^{31}P\{^{1}H\}$ NMR spectrum recorded after 24 h at room temperature in dark, followed by 24 h at room temperature exposed to light (162 MHz, CD_2Cl_2).

6 Characterisation of [Rh(PONOP-*t*Bu)(κ_{Cl}–$ClCH_2Cl$)][$BAr^F{}_4$] A

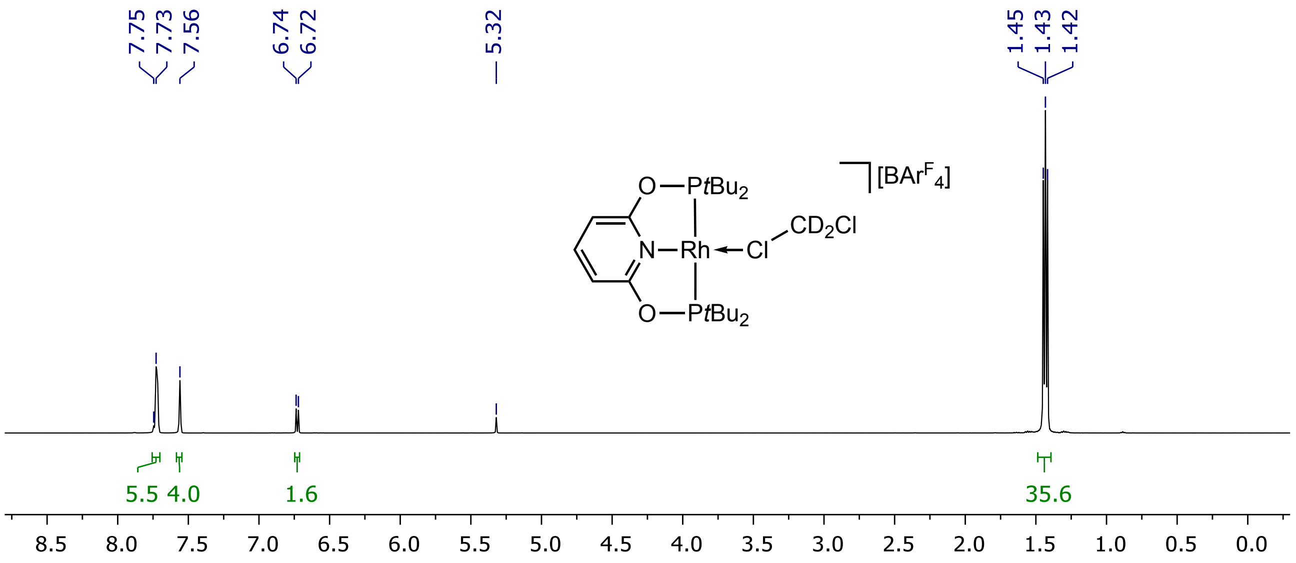

Figure S26. ^{1}H NMR spectrum of d2-**A** (500 MHz, CD_2Cl_2).

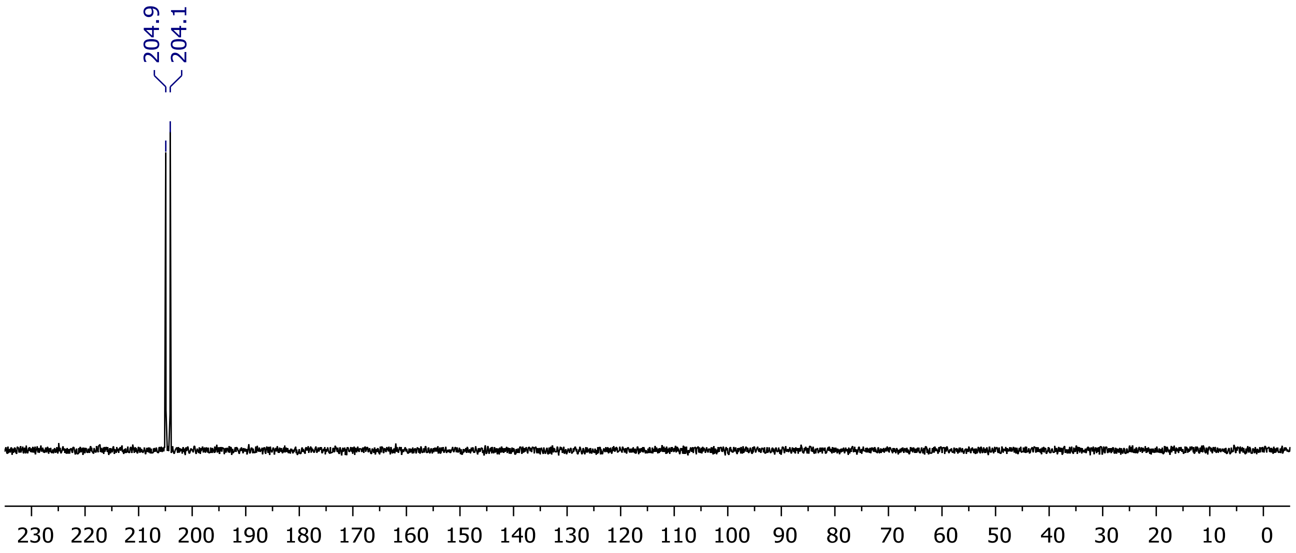

Figure S27. $^{31}P\{^{1}H\}$ NMR spectrum of d2-**A** (162 MHz, CD_2Cl_2).

7 NMR scale reactions of [Rh(PONOP-*t*Bu)(κ_{Cl}–ClCX$_2$Cl)][BAr^F$_4$] (X = H, A; D, d_2-A)

7.1 Stability at room temperature in CD_2Cl_2

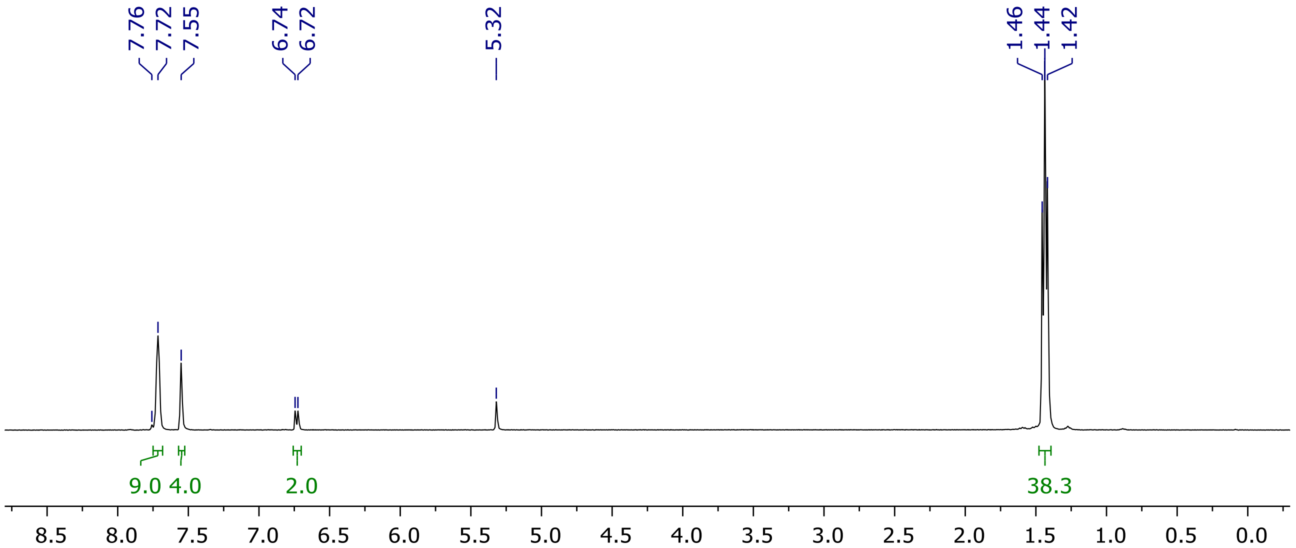

Figure S28. ^{1}H NMR spectrum recorded after 24 h at room temperature in the presence of light (400 MHz, CD_2Cl_2).

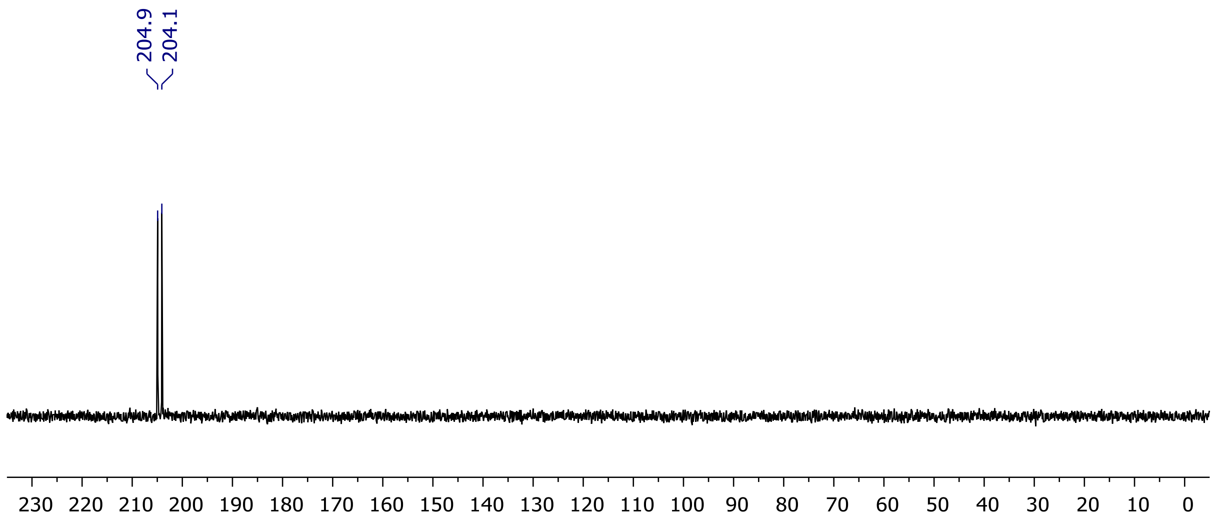

Figure S29. ^{31}P{^{1}H} NMR spectrum recorded after 24 h at room temperature in the presence of light (162 MHz, CD_2Cl_2).

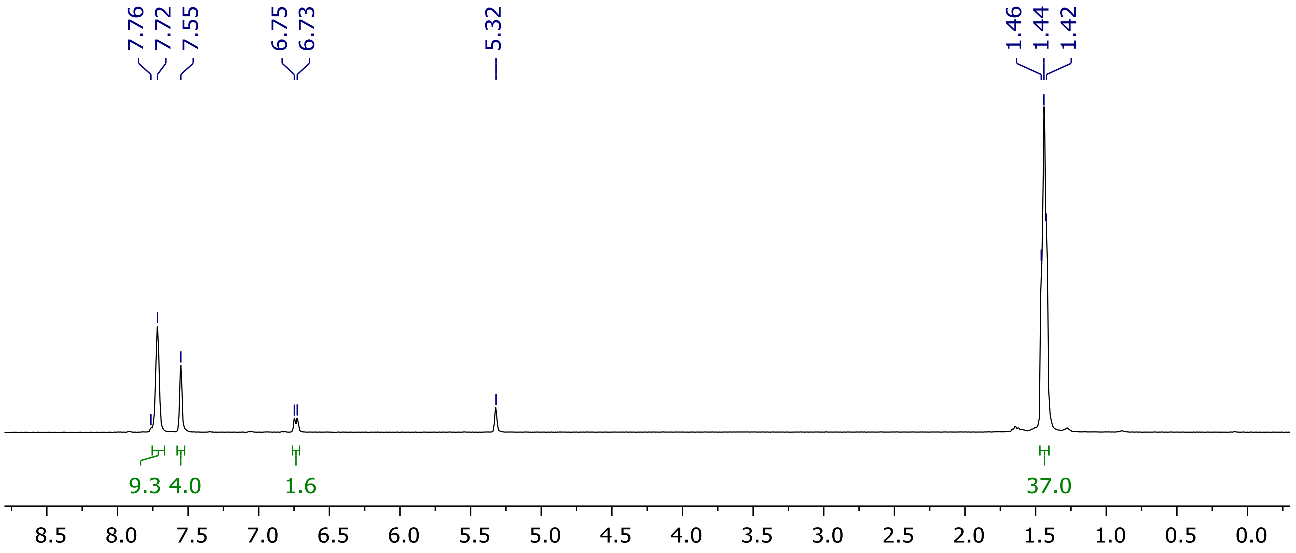

Figure S30. ^{1}H NMR spectrum after 24 h at room temperature in the dark (400 MHz, CD_2Cl_2).

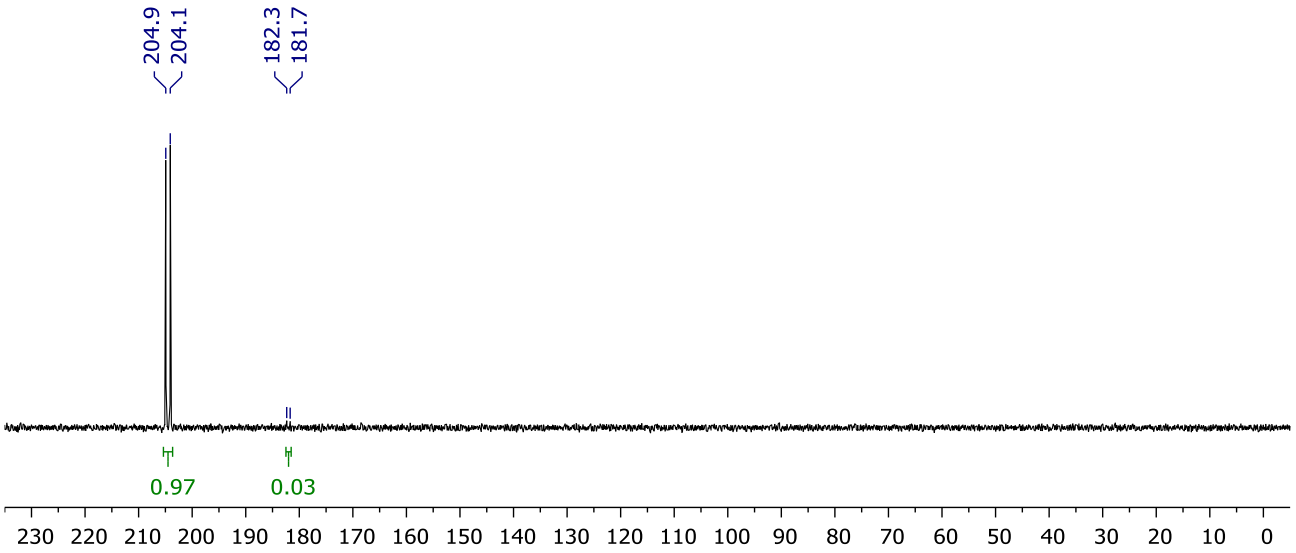

Figure S31. $^{31}P\{^{1}H\}$ NMR spectrum recorded after 24 h at room temperature in the dark (162 MHz, CD_2Cl_2).

7.2 Stability at 50 °C in CD_2Cl_2

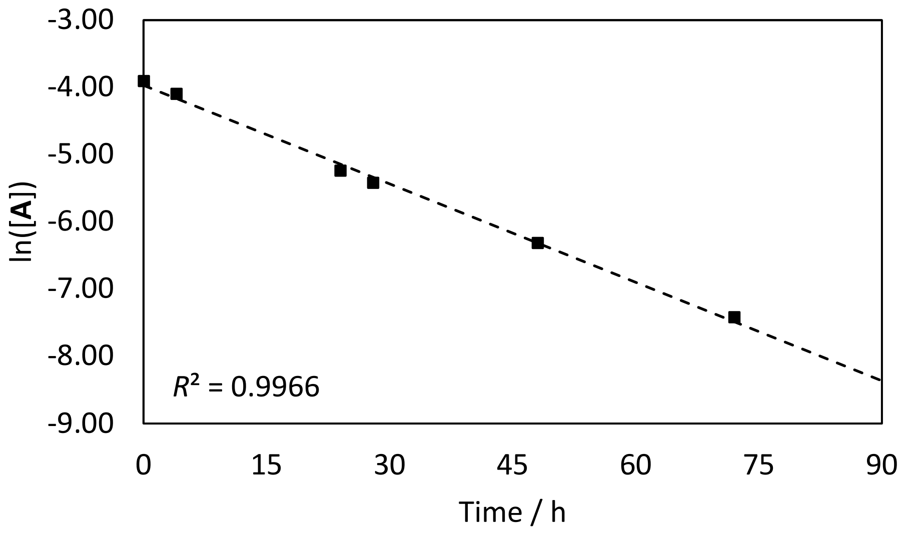

Figure S32. Plot demonstrating pseudo first order consumption of d_2-**A** when heated at 50 °C in the dark. Concentrations determined by integration of ^{1}H NMR data; k_{obs} = 0.049 h^{-1} ($t_{1/2}$ = 14 h).

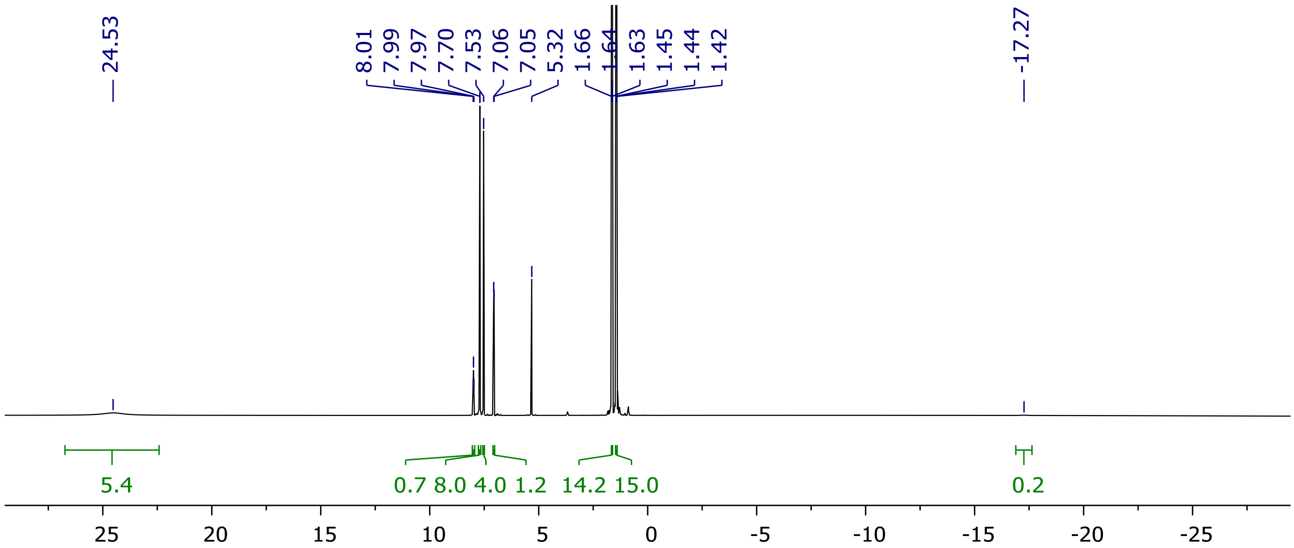

Figure S33. ^{1}H NMR spectrum recorded after 96 h at 50 °C in the dark (500 MHz, CD_2Cl_2).

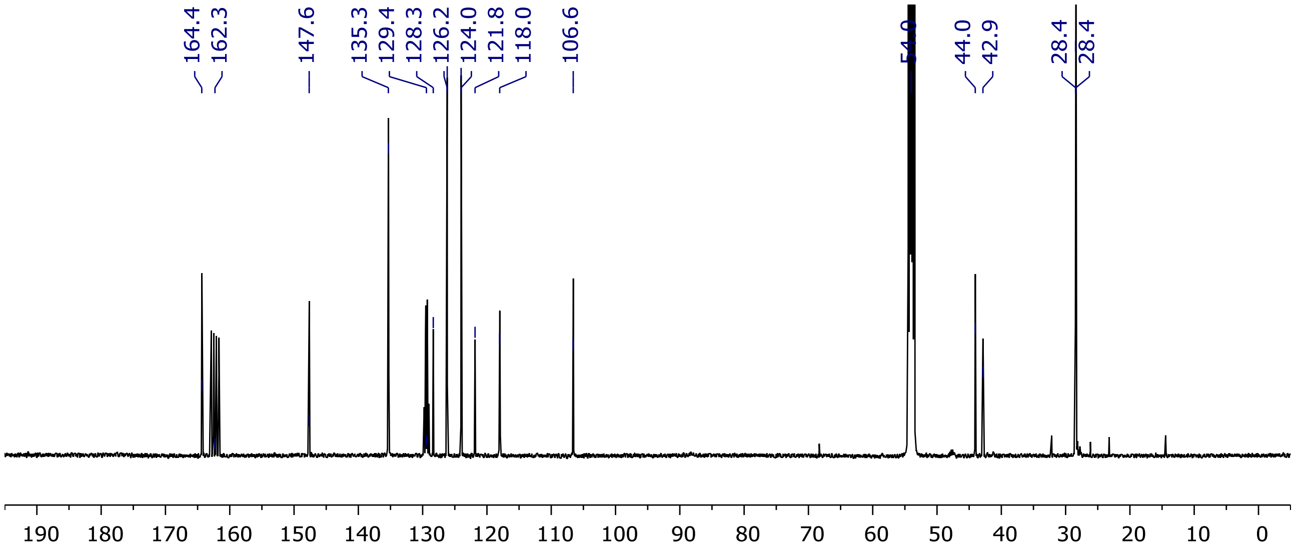

Figure S34. $^{13}C\{^{1}H\}$ NMR spectrum recorded after 96 h at 50 °C in the dark (126 MHz, CD_2Cl_2).

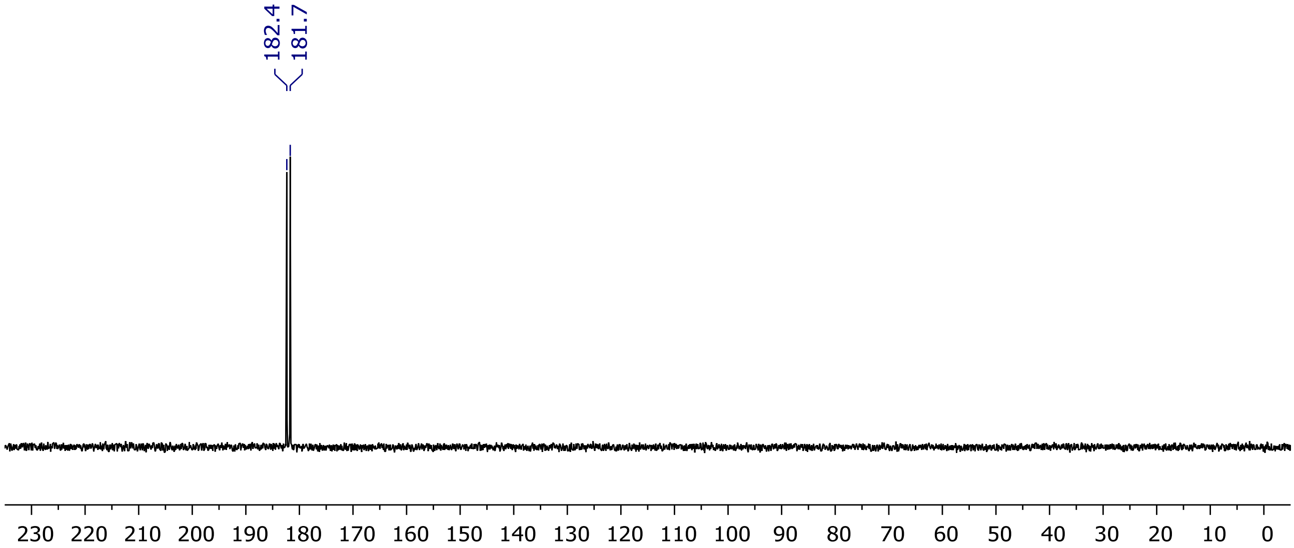

Figure S35. $^{31}P\{^{1}H\}$ NMR spectrum recorded after 96 h at 50 °C in the dark (162 MHz, CD_2Cl_2).

7.3 Solid-state stability

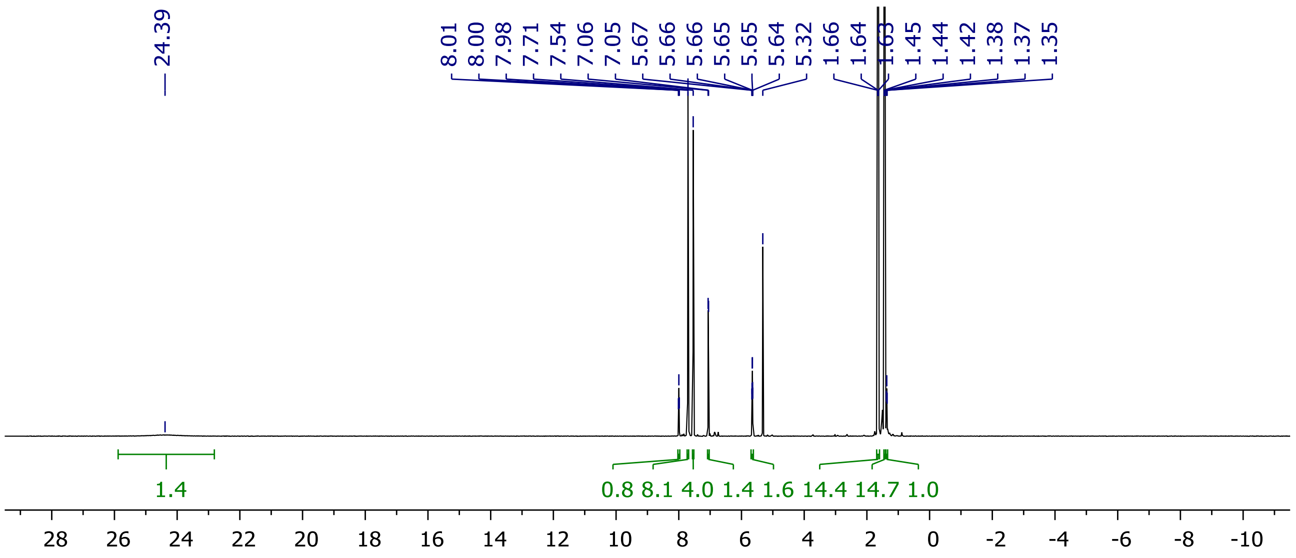

Figure S36. ^{1}H NMR spectrum recorded after 18 h at 110 °C in the solid-state (600 MHz, CD_2Cl_2).

7.4 Characterisation of [Rh(PONOP-*t*Bu)(CH_2Cl)Cl][BAr^F_4] 5

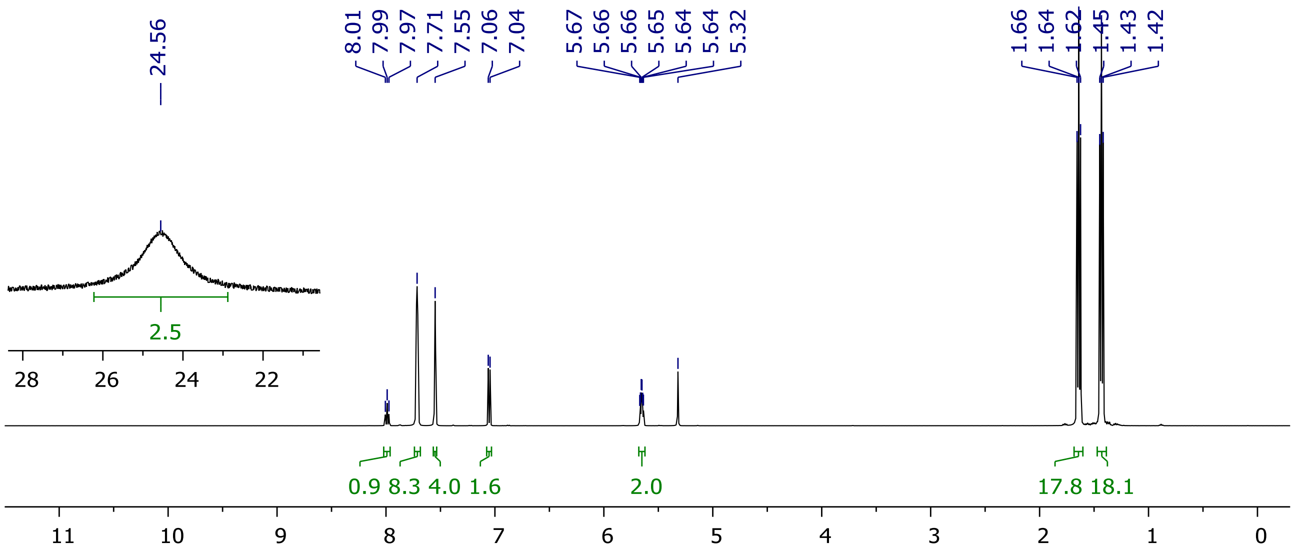

Figure S37. ^{1}H NMR spectrum of 9:1 **5**:**6** (500 MHz, CD_2Cl_2).

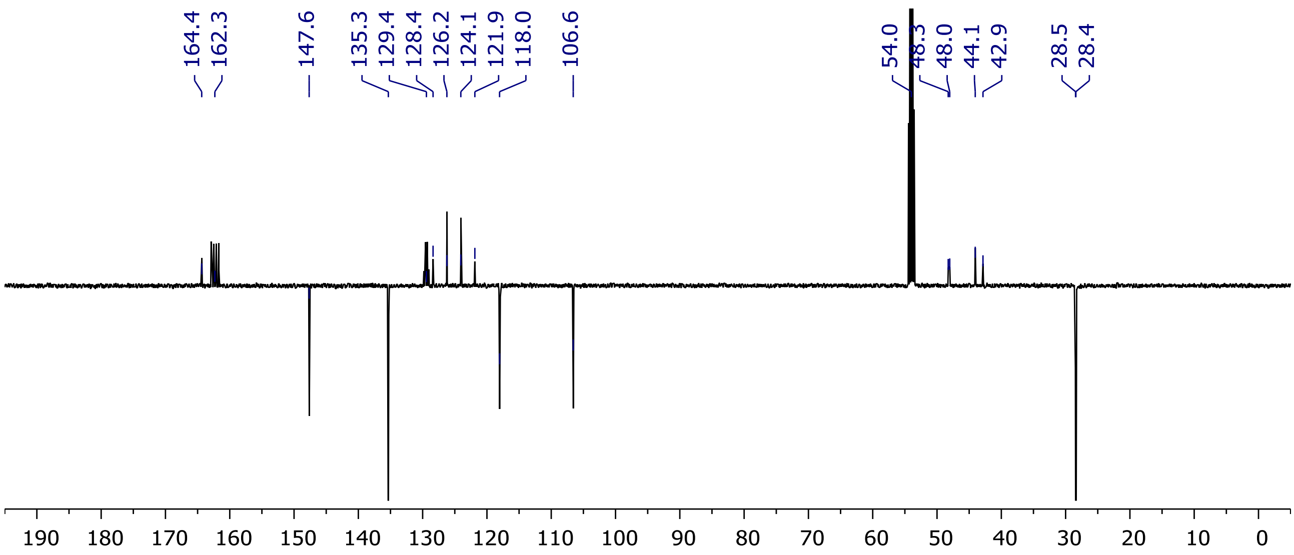

Figure S38. $^{13}C\{^{1}H\}$ APT NMR spectrum of 9:1 **5**:**6** (126 MHz, CD_2Cl_2).

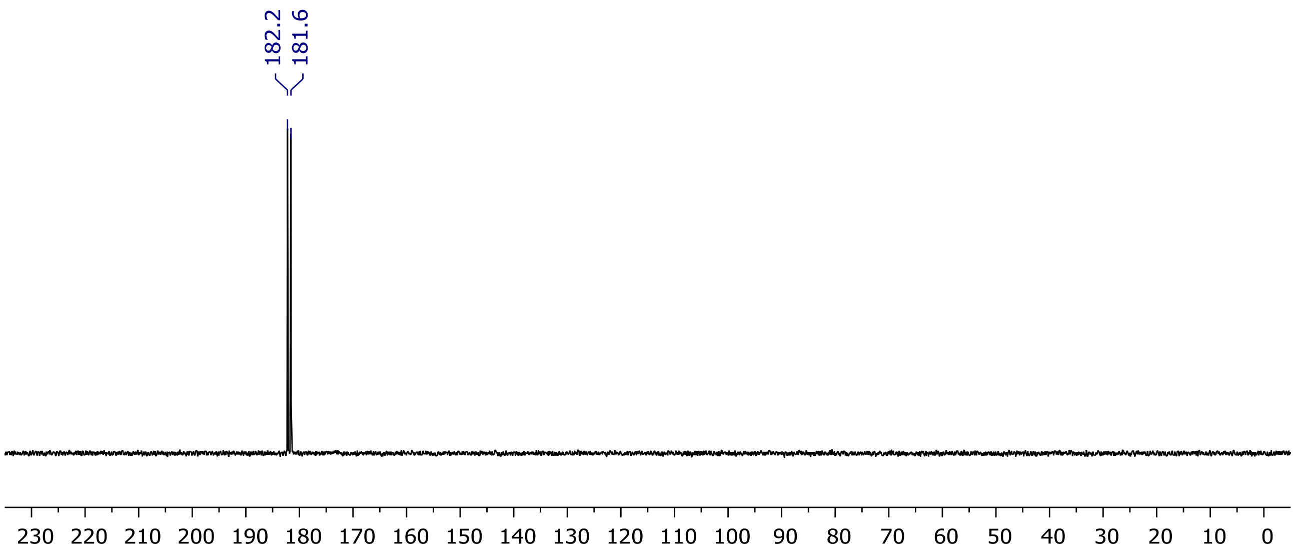

Figure S39. $^{31}P\{^{1}H\}$ NMR spectrum of 9:1 **5**:**6** (162 MHz, CD_2Cl_2).

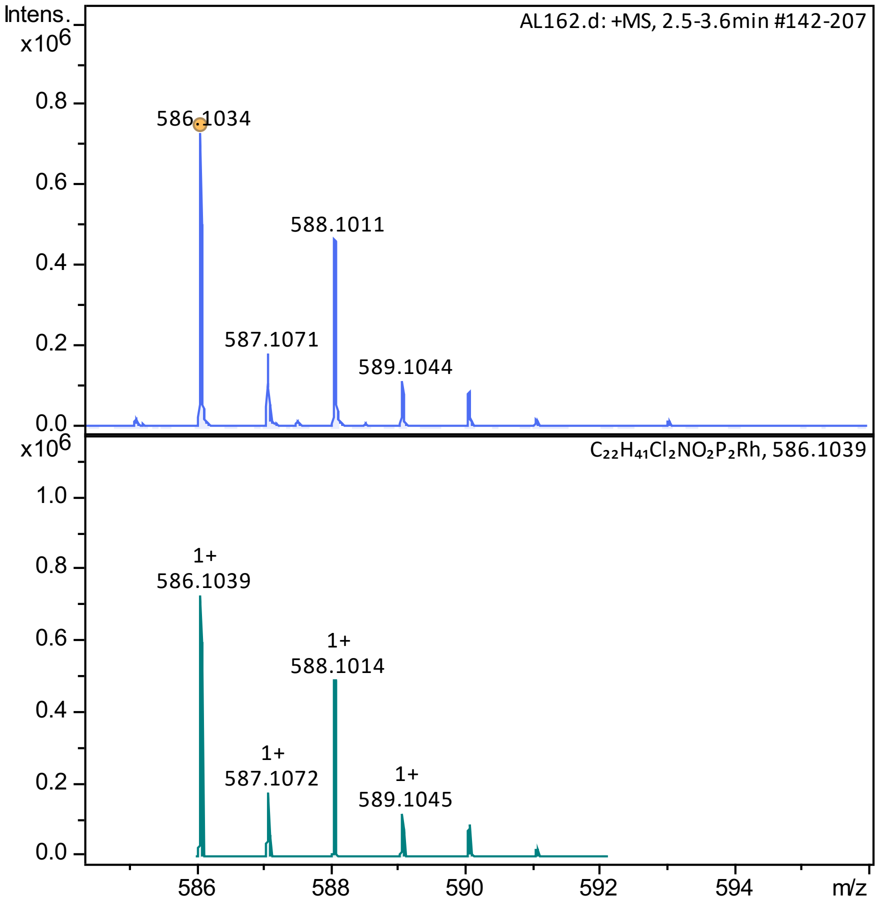

Figure S40. HR ESI-MS of 9:1 **5**:**6**.

8 Cyclic voltammograms for the oxidation of [Rh(PONOP-*t*Bu)Cl]

Cyclic voltammetry experiments were carried out in an inert atmosphere glovebox under argon using a PalmSens EmStat3+ Blue potentiostat and a 3-electrode set-up comprising a glassy carbon (CH Instruments, 3.0 mm-diameter) working electrode (WE), coiled platinum wire counter electrode (CE) and silver wire quasi-reference electrode (RE). All potentials are calibrated to the ferrocene/ferrocenium (Fc/Fc^+) redox couple, which was used as an internal standard. The half-wave potentials, $E_{1/2}$, were determined from: $E_{1/2} = (E_P^{red} + E_P^{ox})/2$, where E_P^{red} and E_P^{ox} are the reduction and oxidation peak potential values, respectively.

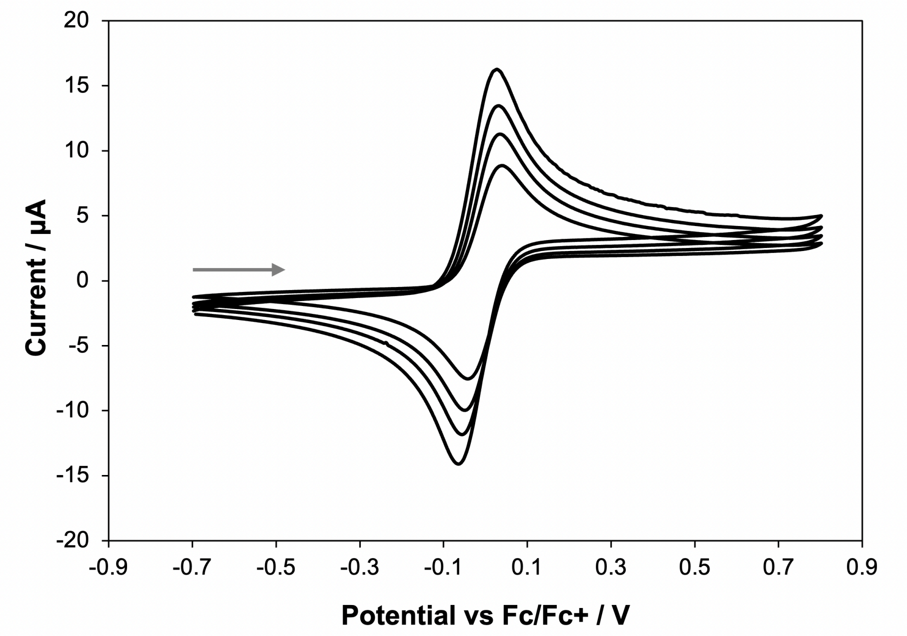

Figure S41. Cyclic voltammograms for the oxidation of [Rh(PONOP-*t*Bu)Cl] in 1,2-difluorobenzene at room temperature (2 mM complex; 0.2 M [$^nBu_4N$][BAr^F_4] electrolyte; glassy carbon working electrode, coiled Pt wire counter electrode and Ag wire quasi-reference electrode; scan rates = 30, 50, 70 and 100 mV·s^{-1}). Calculated $E_{1/2}$ = -0.01 V vs Fc/Fc+.

9 Characterisation of [Rh(PONOP-*t*Bu)Cl][BAr$^F_4$] 6

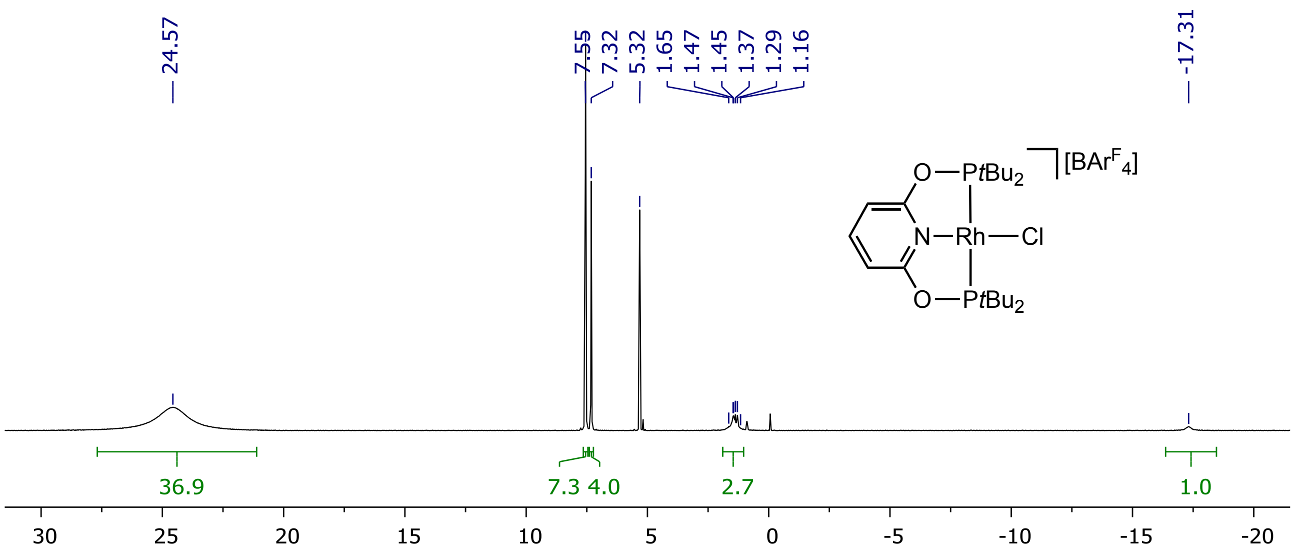

Figure S42. ^{1}H NMR spectrum of **6** (400 MHz, CD_2Cl_2).

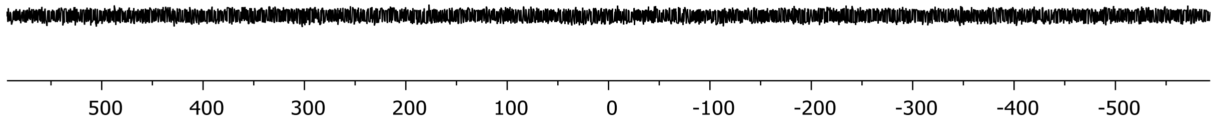

Figure S43. ^{31}P{^{1}H} NMR spectrum of **6** (162 MHz, CD_2Cl_2).

Measurements of ac susceptibility, χ_{ac}, versus temperature, T, were made between 1.8 and 12 K using a Quantum Design MPMS-5S SQUID magnetometer in an ac field of 3 Oe at frequencies between 3 and 1157 Hz (Figure S44). These experiments reveal $\chi_{ac}(T)$ for **6** is frequency independent, with a Curie–Weiss behaviour down to lowest temperature measured. A fit using a Curie-Weiss model gives an effective moment of 2.20(2) μ_B and a Weiss temperature Θ_W of -0.01(4) K, in agreement with the $\chi_{dc}(T)$ data. Measurements of $\chi_{ac}(T)$ in a small (20 Oe) dc field are reversible, with ZFCW and FCC curves coinciding with no signature of long-range magnetic ordering or spin-freezing.

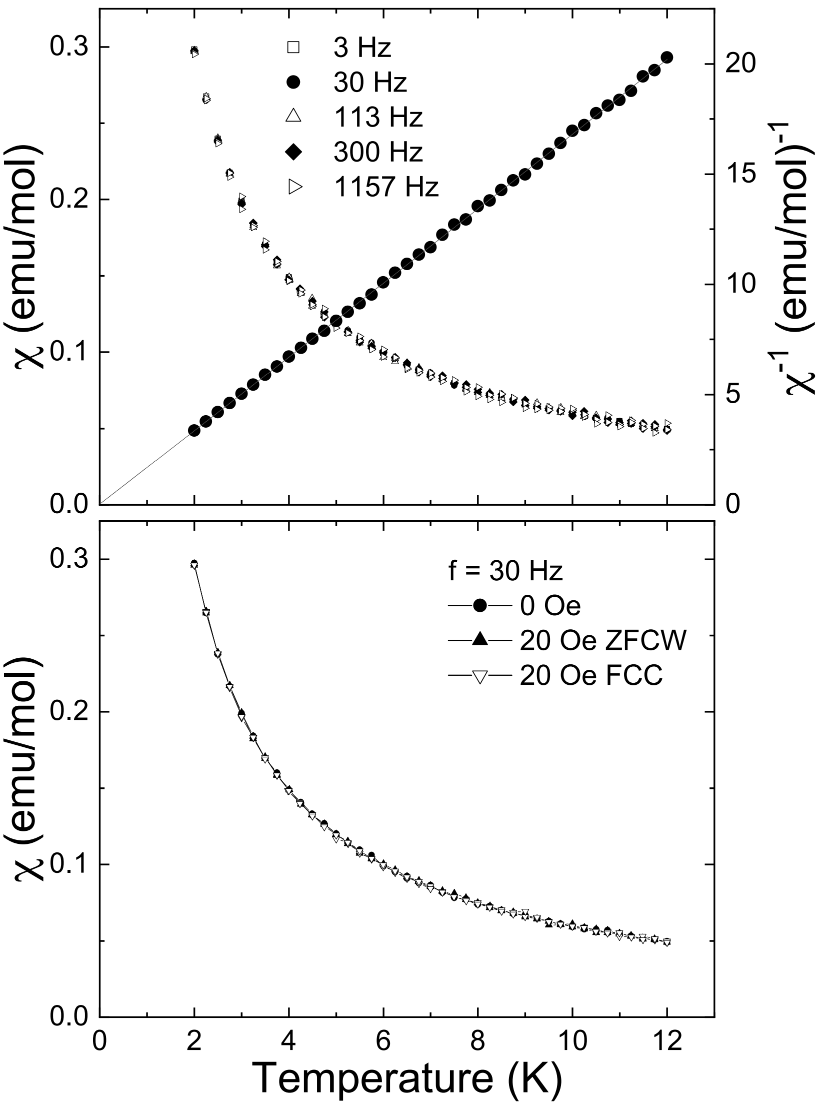

Figure S44. Upper panel: Temperature dependence of the ac magnetic susceptibility, $\chi_{ac}(T)$, and the inverse ac susceptibility versus temperature, $\chi_{AC}^{-1}(T)$, for **6** at different frequencies with an ac field of 3 Oe. The data were collected while cooling. The solid line shows a fit using a Curie-Weiss law $\left[\chi_{AC}(T) = \frac{C}{(T-\Theta_W)} + \chi_0\right]$ between 2 and 12 K. Lower panel: Temperature dependence of the ac magnetic susceptibility, $\chi_{ac}(T)$, for **6** collected while cooling in zero dc field and in zero-field-cooled warming and field-cooled cooling modes in a dc field of 20 Oe. The measurements were made in an ac field of 3 Oe at 30 Hz.

10 NMR scale reactions of [Rh(PONOP-*t*Bu)(CH_2Cl)Cl][BAr$^F{}_4$] 5

10.1 Stability at room temperature in CD_2Cl_2

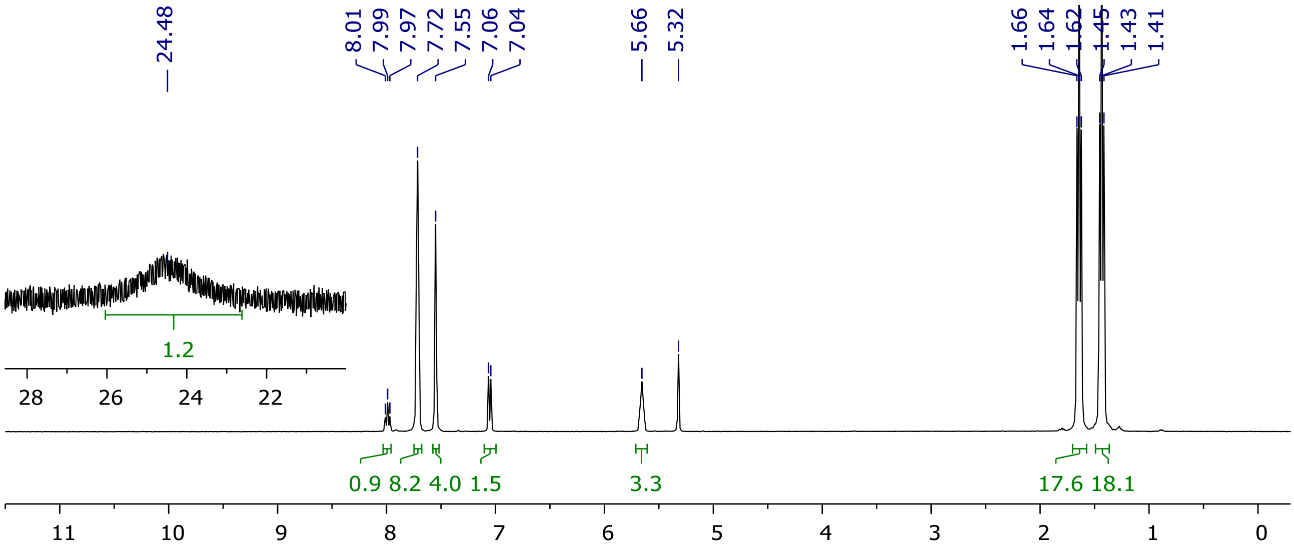

Figure S45. ^{1}H NMR spectrum recorded after 48 h at room temperature in the dark (400 MHz, CD_2Cl_2).

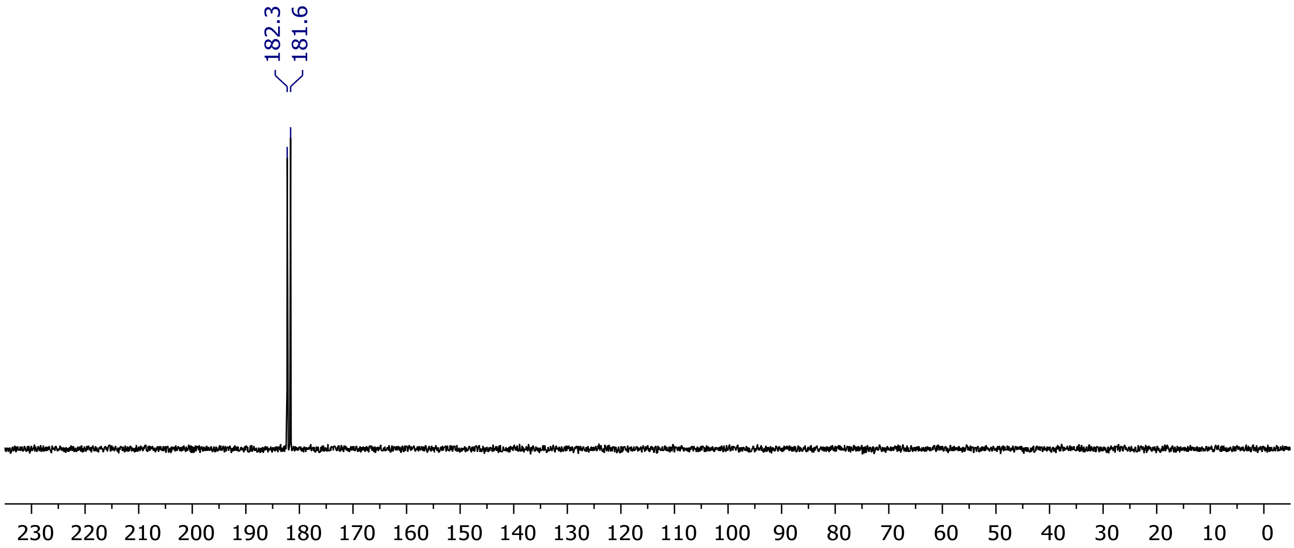

Figure S46. ^{31}P{^{1}H} NMR spectrum recorded after 48 h at room temperature in the dark (162 MHz, CD_2Cl_2).

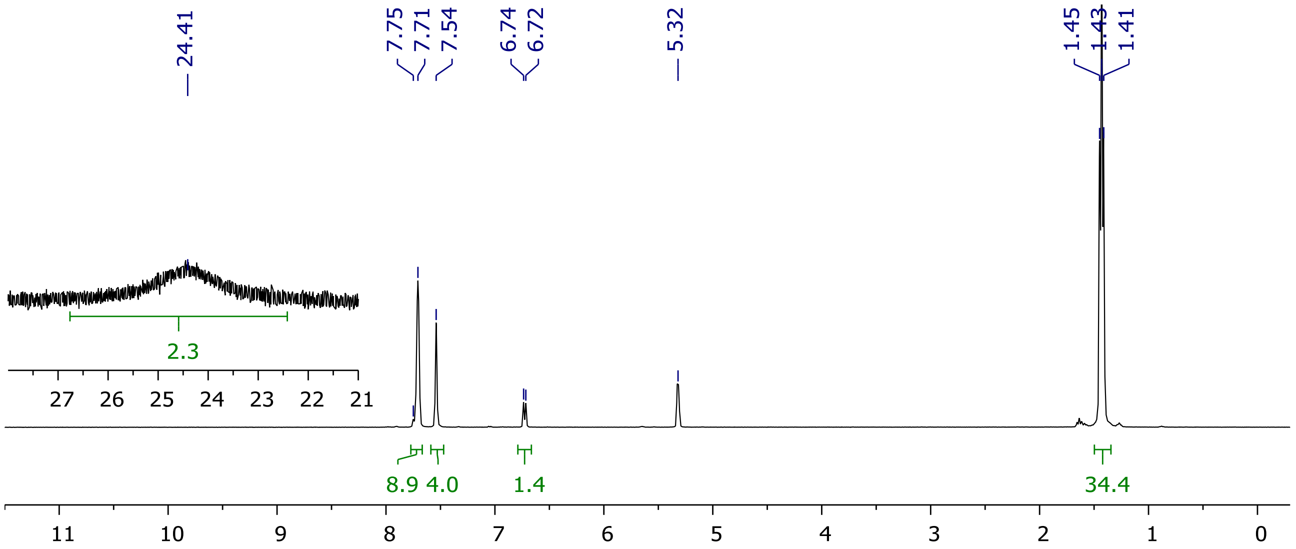

Figure S47. ^{1}H NMR spectrum recorded after 4 h at room temperature in the presence of light (400 MHz, CD_2Cl_2).

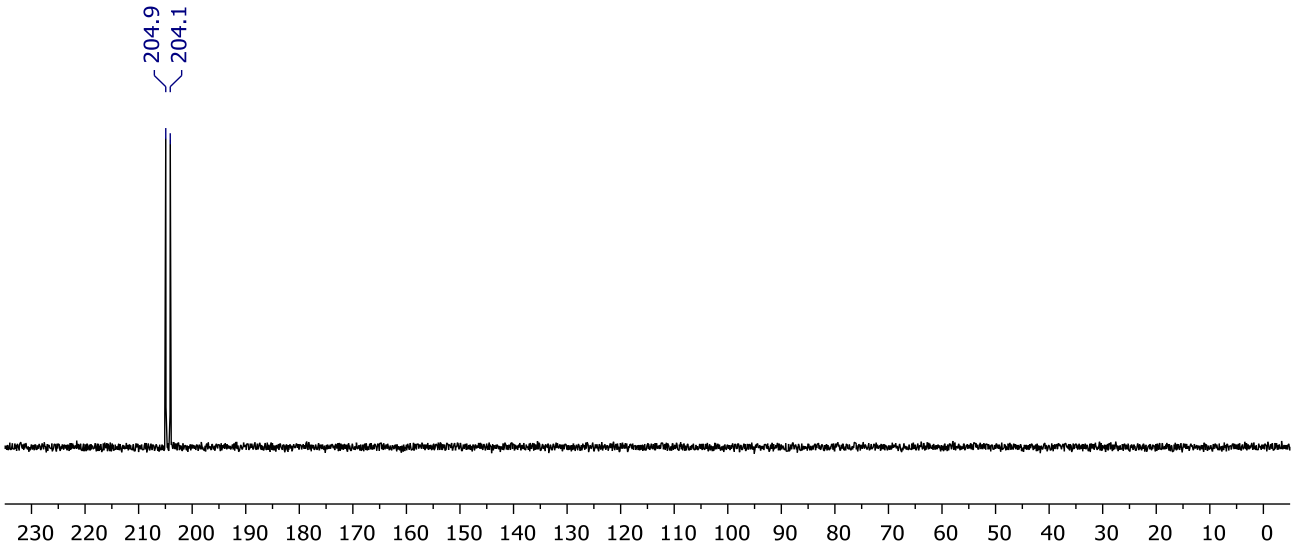

Figure S48. $^{31}P\{^{1}H\}$ NMR spectrum recorded after 4 h at room temperature in the presence of light (162 MHz, CD_2Cl_2).

10.2 Stability in the presence of TEMPO in CD_2Cl_2

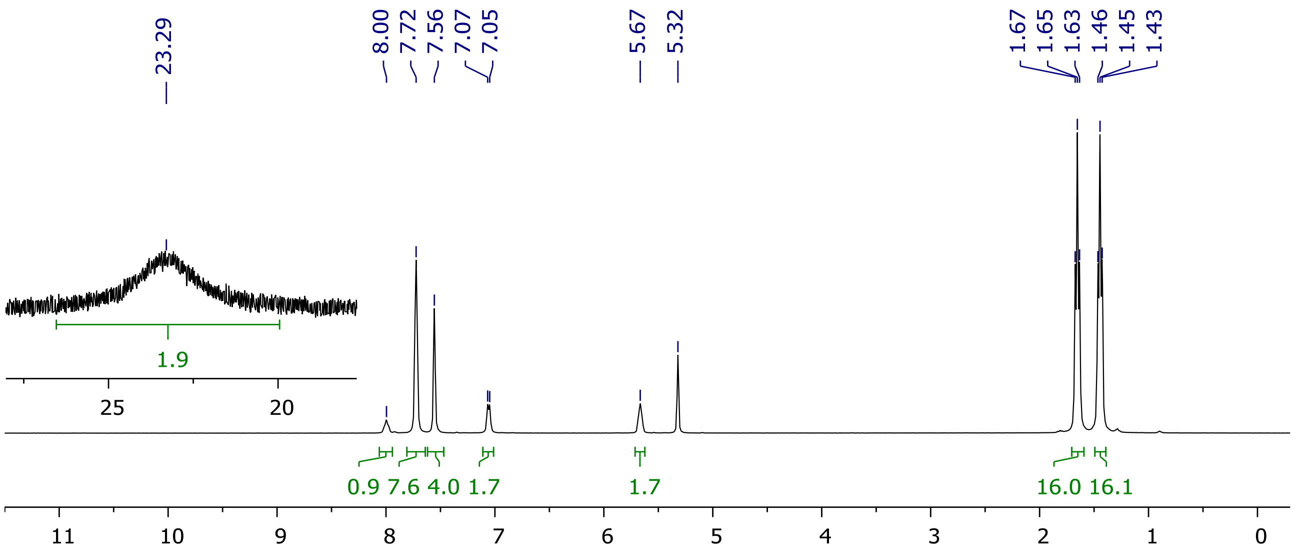

Figure S49. ^{1}H NMR spectrum recorded after 24 h at room temperature in the dark (400 MHz, CD_2Cl_2).

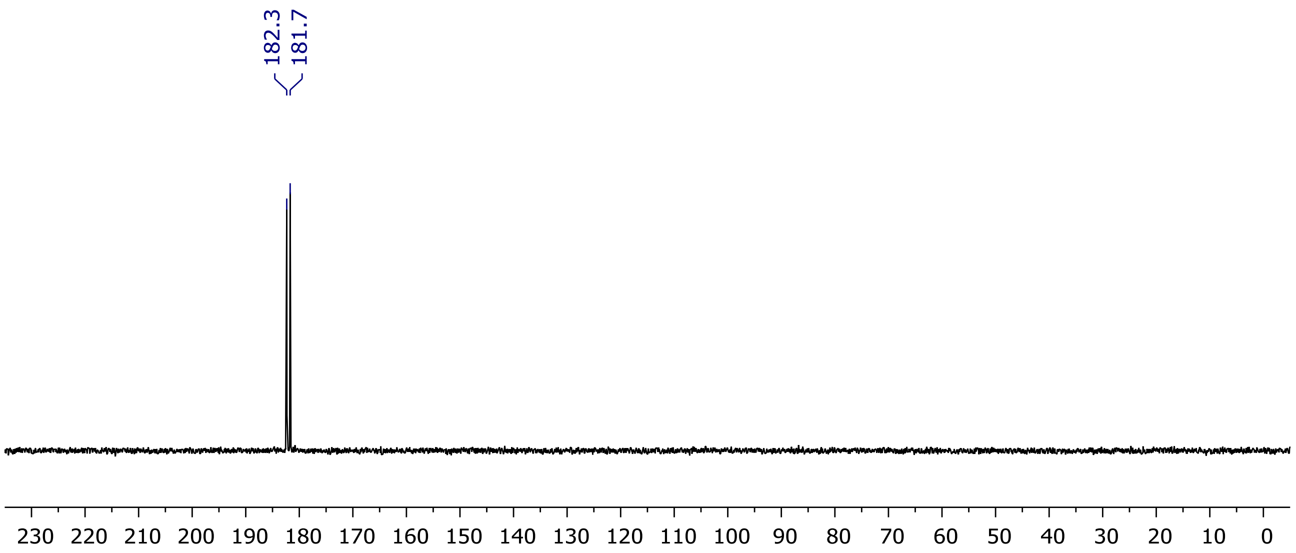

Figure S50. $^{31}P\{^{1}H\}$ NMR spectrum recorded after 24 h at room temperature in the dark (162 MHz, CD_2Cl_2).

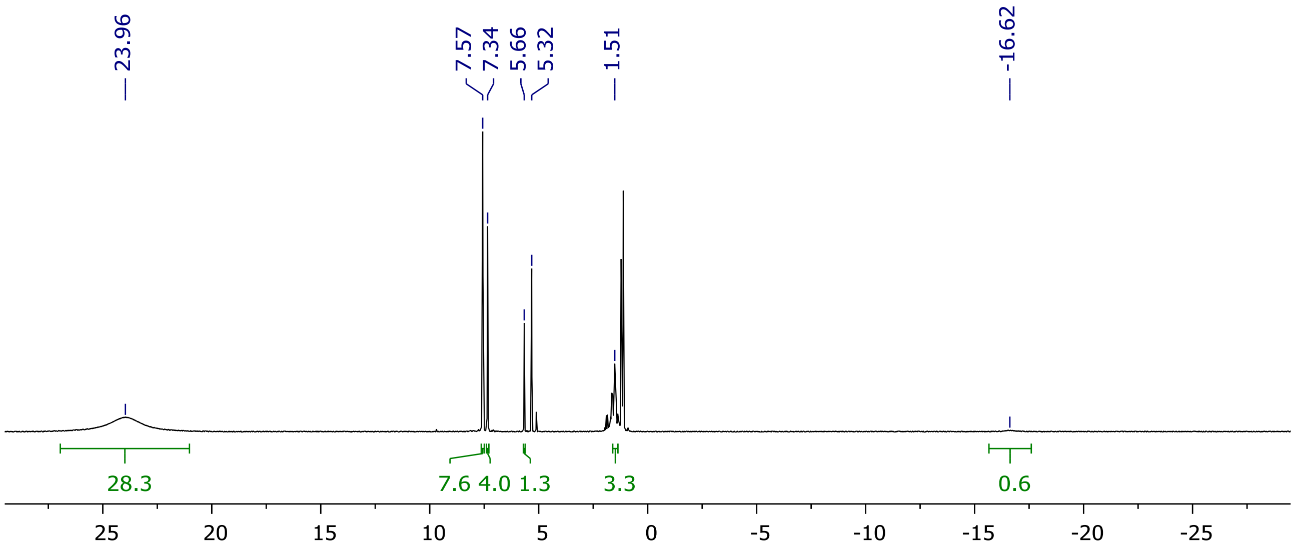

Figure S51. ^{1}H NMR spectrum recorded after 4 h at room temperature in the presence of light (400 MHz, CD_2Cl_2).

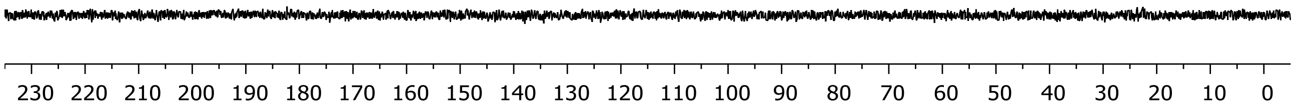

Figure S52. $^{31}P\{^{1}H\}$ NMR spectrum recorded after 4 h at room temperature in the presence of light (162 MHz, CD_2Cl_2).

11 NMR scale reactions of [Rh(PONOP-*t*Bu)Cl][BAr$^F_4$] 6

11.1 Stability at 50 °C in CD_2Cl_2

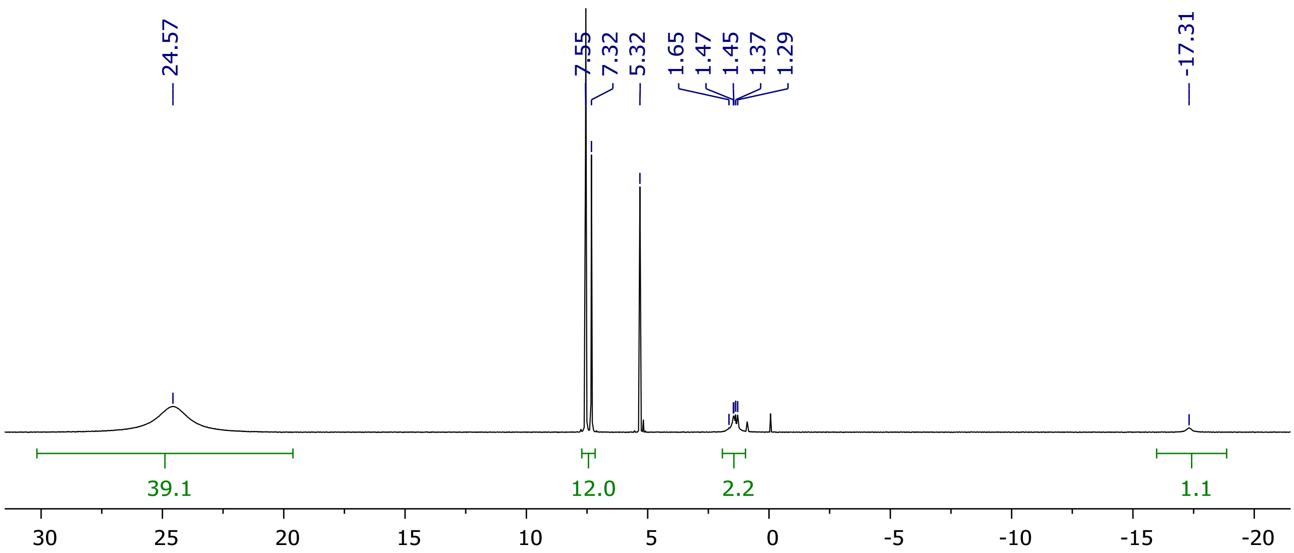

Figure S53. ^1H NMR spectrum recorded after 24 h in dark at 50 °C, followed by 24 h at 50 °C exposed to light (400 MHz, CD_2Cl_2).

11.2 Reaction with dihydroanthracene

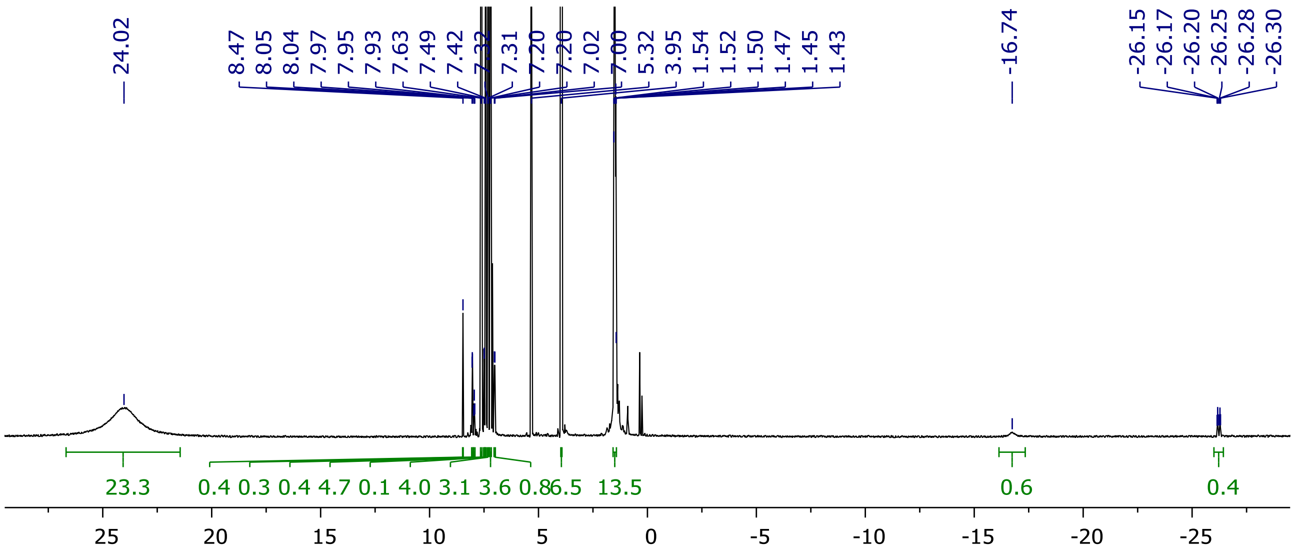

Figure S54. ^{1}H NMR spectrum recorded after heating at 50 °C for 2 weeks (400 MHz, CD_2Cl_2).

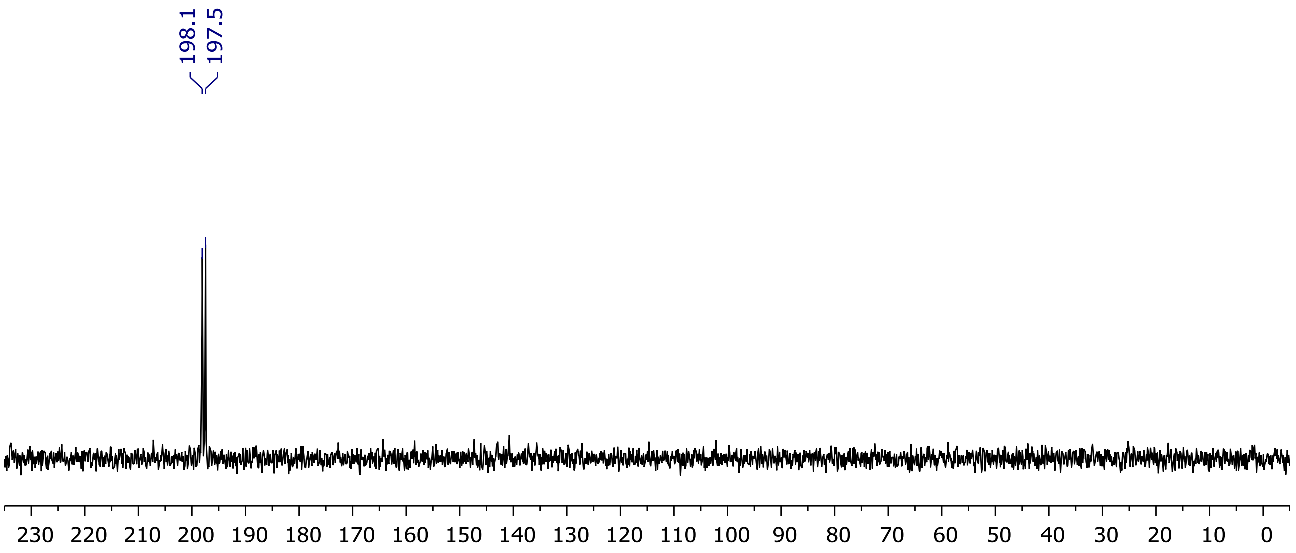

Figure S55. $^{31}P\{^{1}H\}$ spectrum recorded after heating at 50 °C for 2 weeks (162 MHz, CD_2Cl_2).

12 Characterisation of $[Rh(PONOP\text{-}tBu)(\kappa_{Cl}\text{–}ClCy)][BAr^F{}_4]$ 2

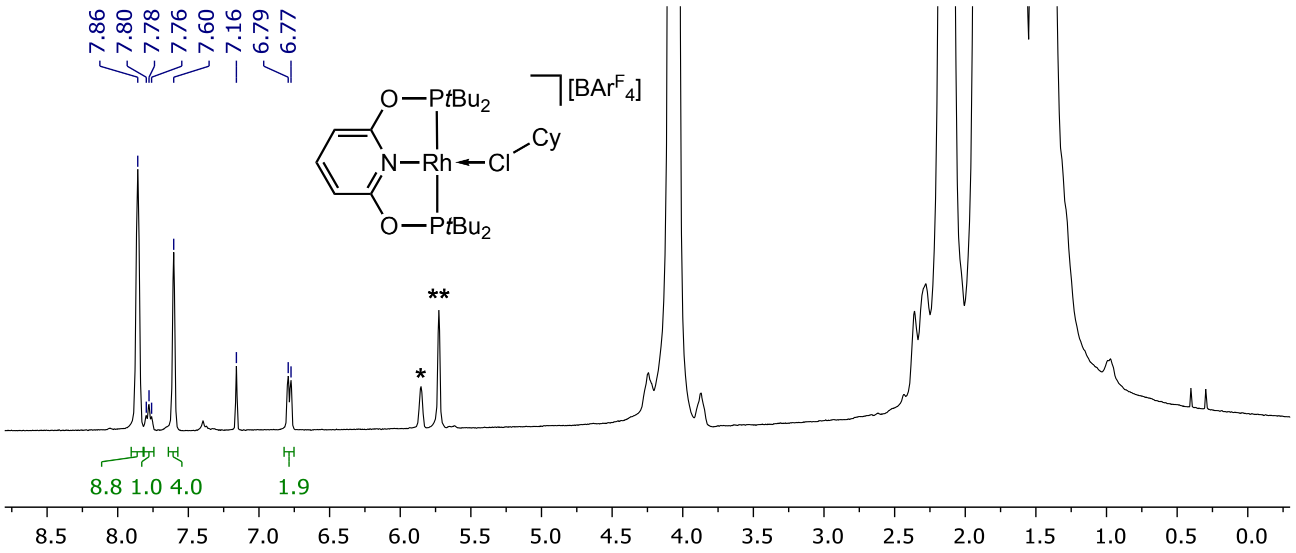

Figure S56. ^{1}H NMR spectrum of **2** (400 MHz, CyCl). * = impurity in CyCl, ** = cyclohexene impurity in CyCl

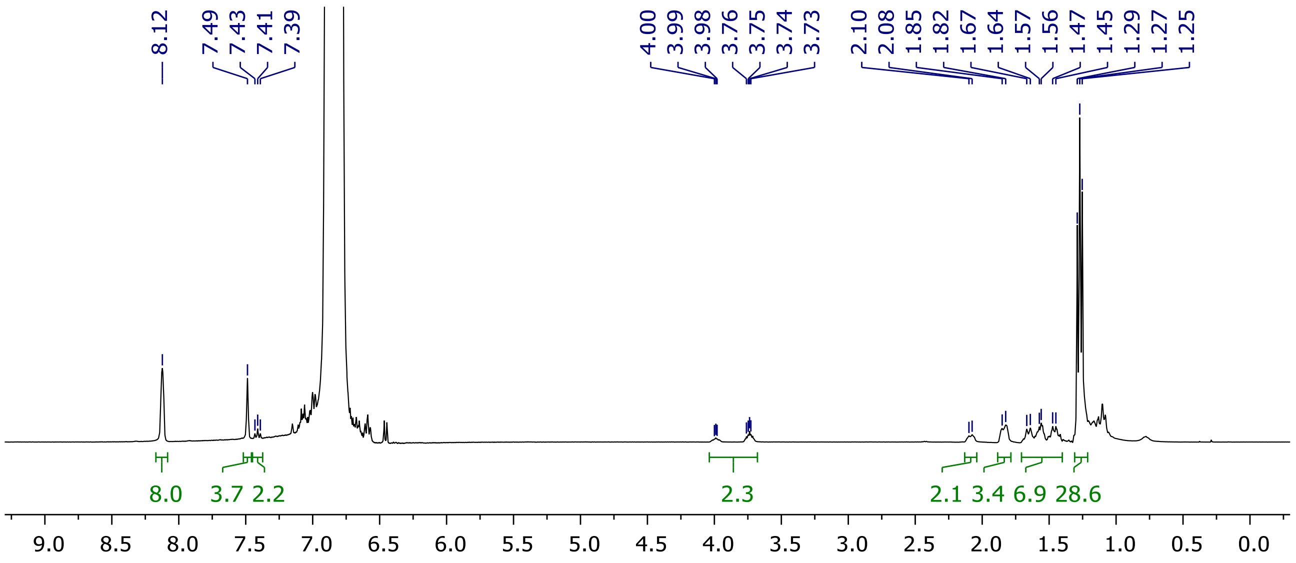

Figure S57. ^{1}H NMR spectrum of **2** recorded within 5 min in 1,2-difluorobenzene to corroborate microanalysis findings (400 MHz). Integration of the ClC<u>H</u> signals δ 3.6 – 4.1 confirms a total of two CyCl molecules per rhodium.

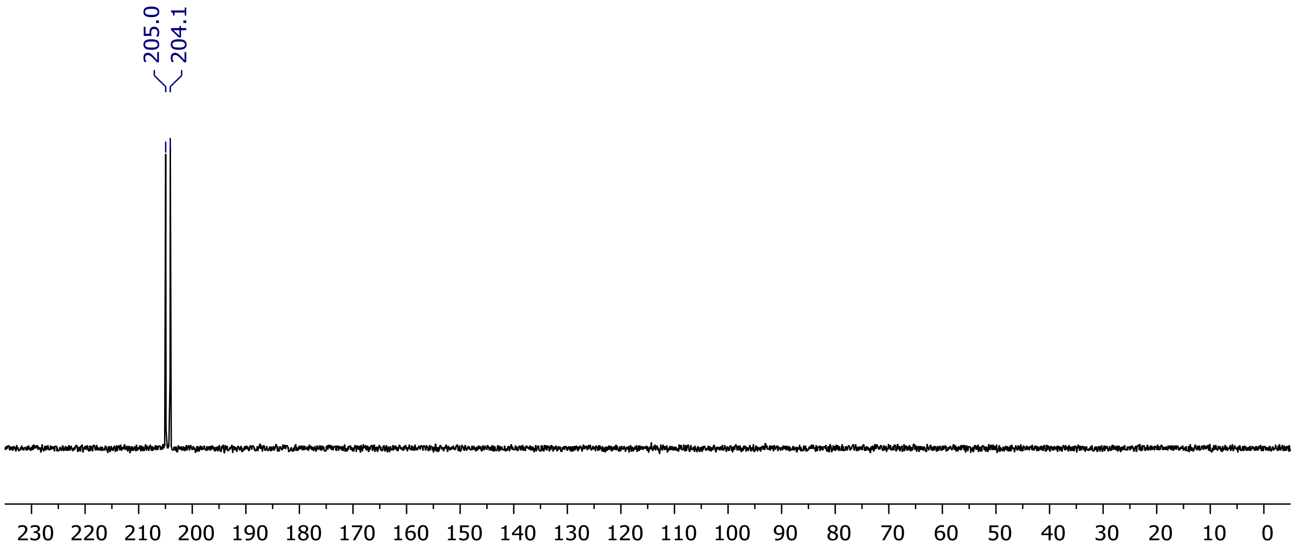

Figure S58. $^{31}P\{^{1}H\}$ NMR spectrum of **2** (162 MHz, CyCl).

13 NMR scale reactions of [Rh(PONOP-*t*Bu)(κ_{Cl}–ClCy)][BAr$^F{}_4$] 2

13.1 Stability at room temperature in CyCl

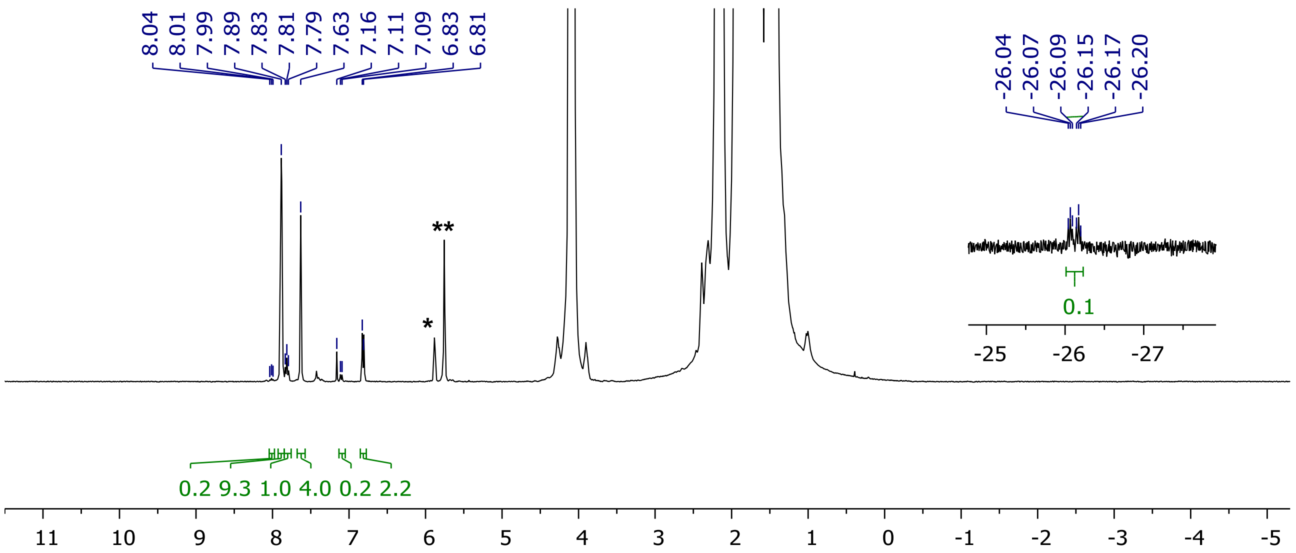

Figure S59. ^{1}H NMR spectrum recorded after 24 h at room temperature in the dark (400 MHz, CyCl). Similar data observed when exposed to light. * = impurity in CyCl, ** = cyclohexene impurity in CyCl

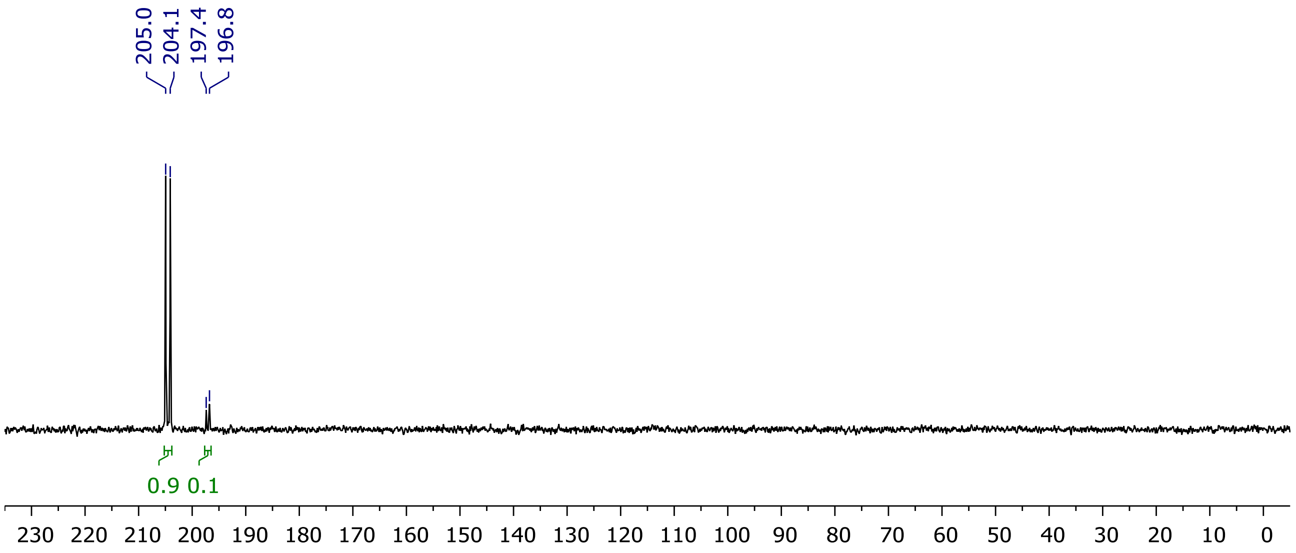

Figure S60. ^{31}P{^{1}H} NMR spectrum recorded after 24 h at room temperature in the dark (162 MHz, CyCl). Similar data observed when exposed to light.

13.2 Stability at 50 °C in CyCl

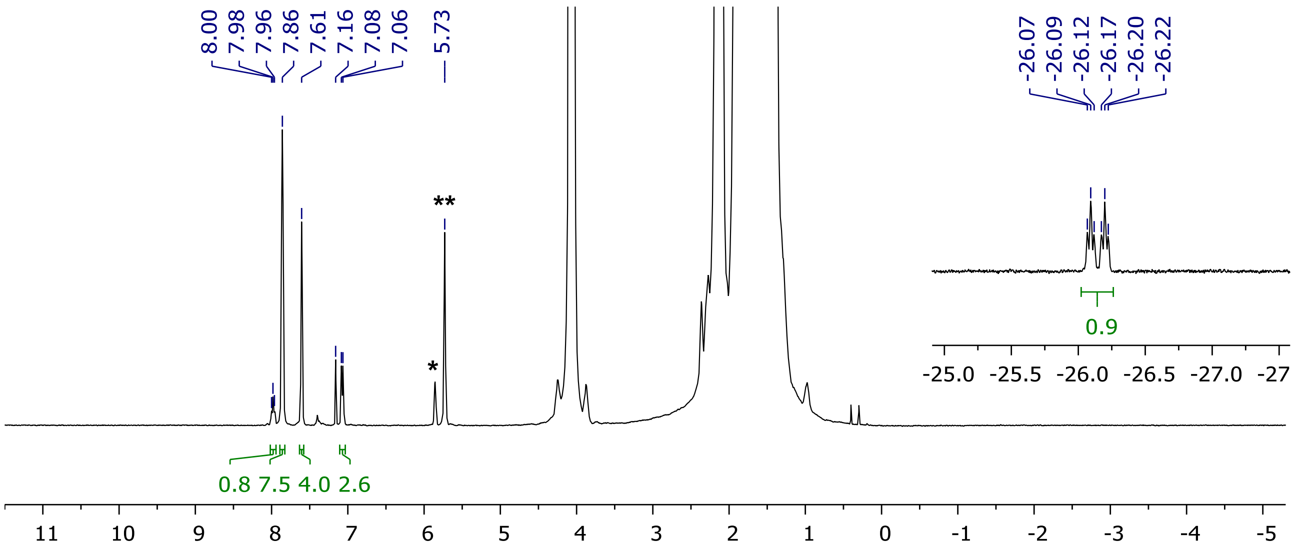

Figure S61. ^{1}H NMR spectrum recorded after 24 h at 50 °C in the dark (400 MHz, CyCl). Similar data observed when exposed to light. * = impurity in CyCl, ** = cyclohexene from reaction (integrating to 2H) and present in the CyCl solvent.

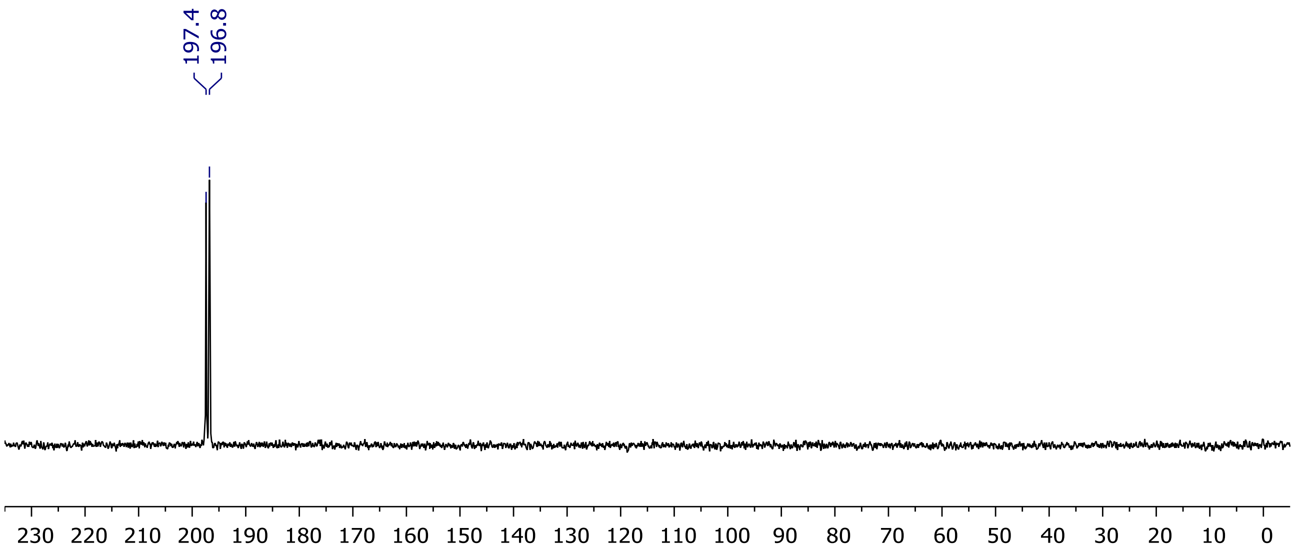

Figure S62. ^{31}P{^{1}H} NMR spectrum recorded after 24 h at 50 °C in the dark (162 MHz, CyCl). Similar data observed when exposed to light.

14 Characterisation of [Rh(PONOP-*t*Bu)(H)Cl][BAr^F$_4$] 7

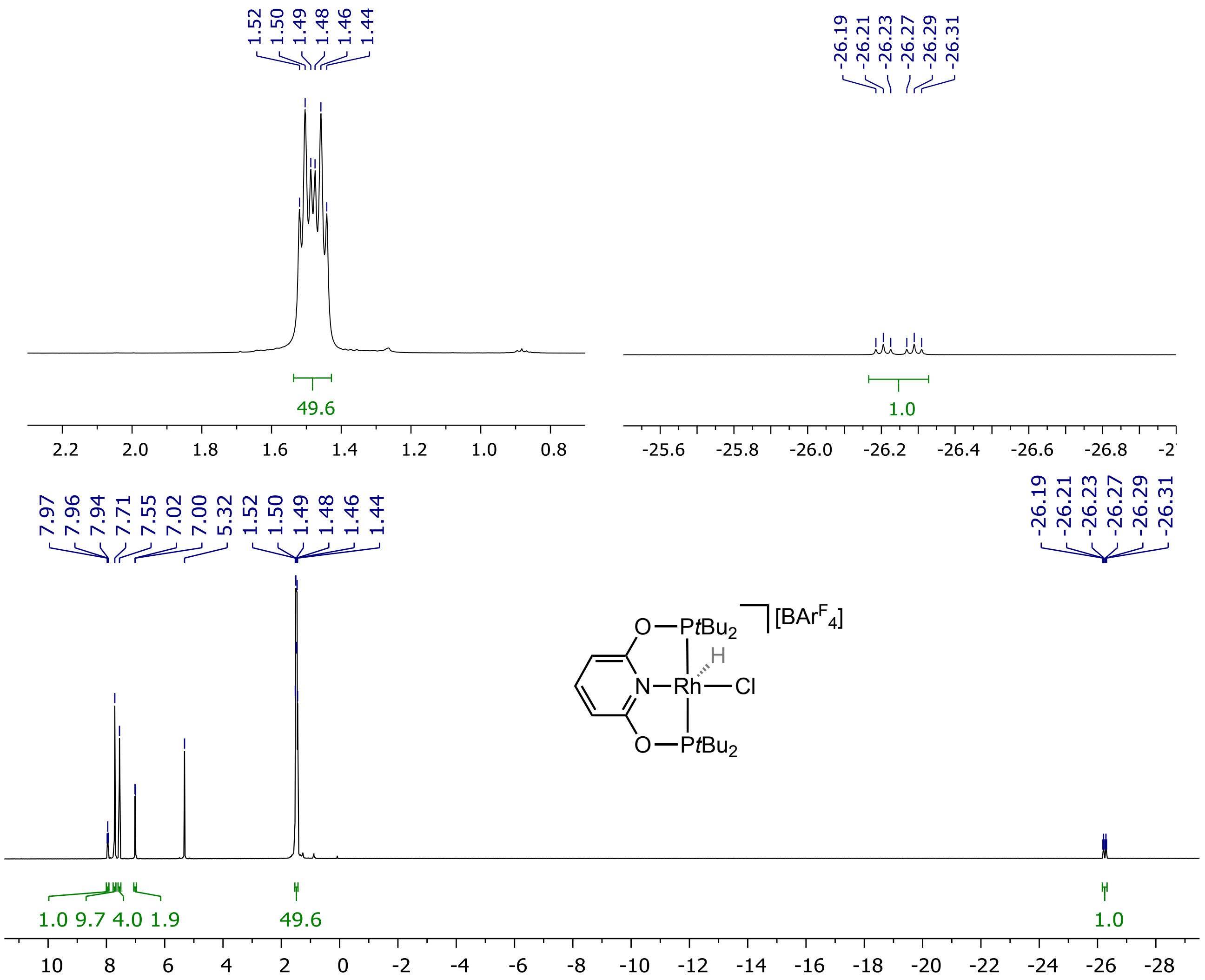

Figure S63. ^{1}H NMR spectrum of **7** (500 MHz, CD_2Cl_2).

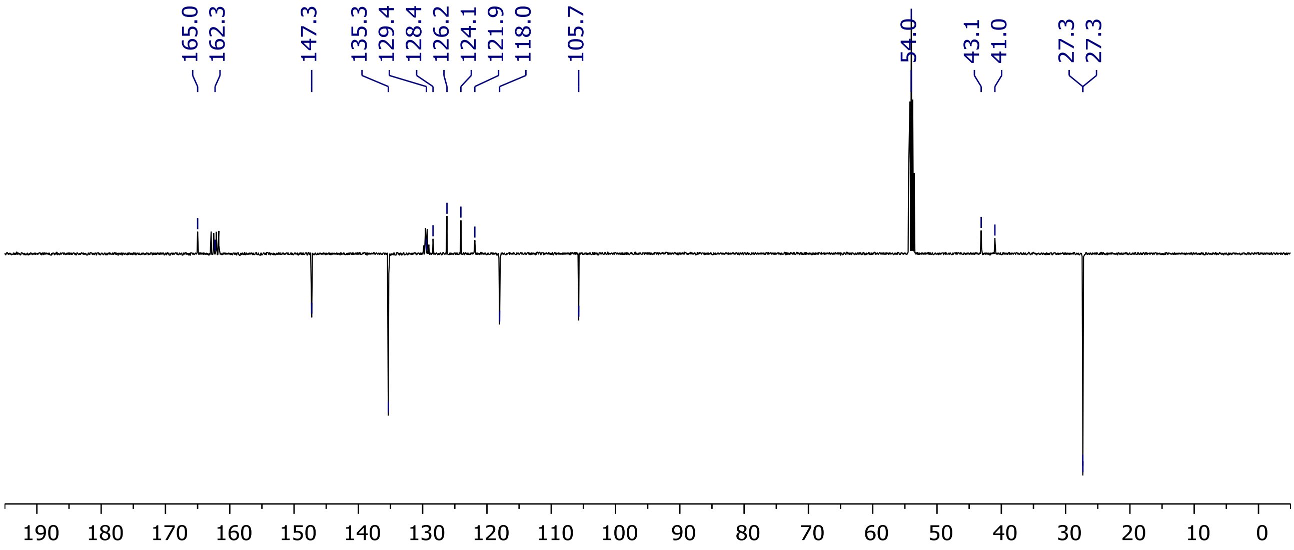

Figure S64. $^{13}C\{^{1}H\}$ APT NMR spectrum of **7** (126 MHz, CD_2Cl_2).

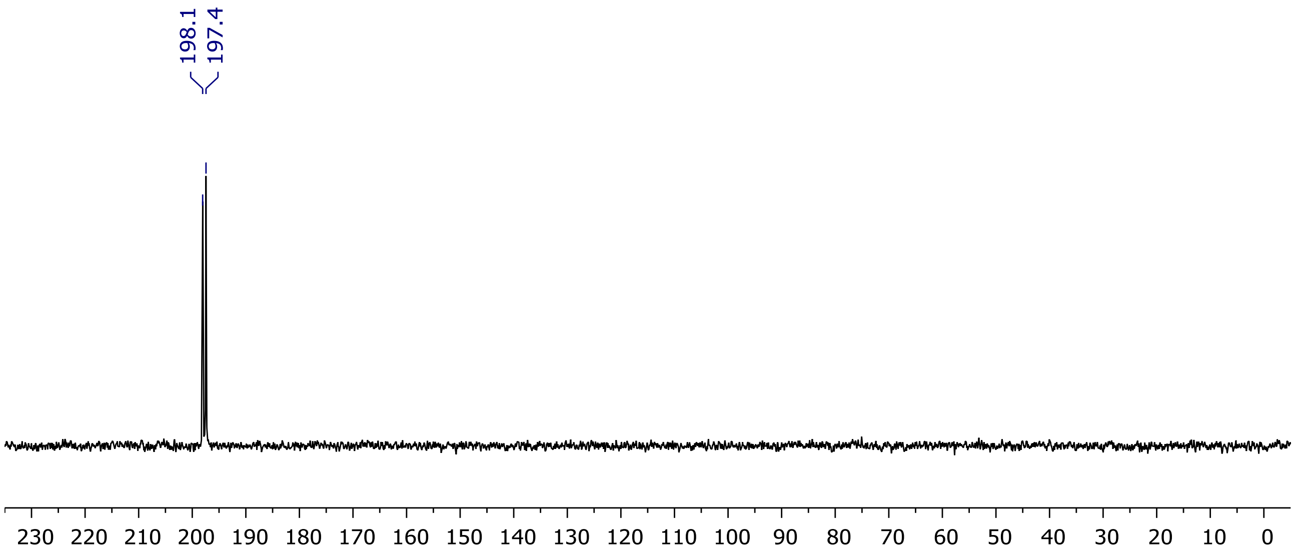

Figure S65. $^{31}P\{^{1}H\}$ NMR spectrum of **7** (162 MHz, CD_2Cl_2).

15 NMR scale reactions of [Rh(PONOP-*t*Bu)(H)Cl][BAr$^F_4$] 7

15.1 Stability at room temperature in CD_2Cl_2

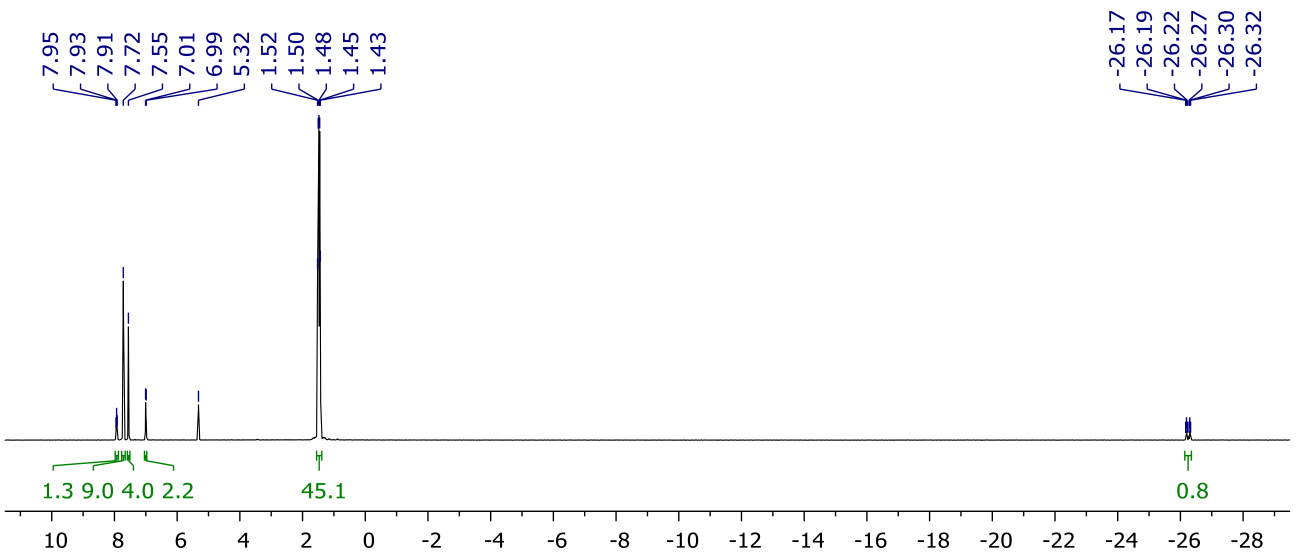

Figure S66. ^{1}H NMR spectrum recorded after 72 h at room temperature in the dark (400 MHz, CD_2Cl_2).

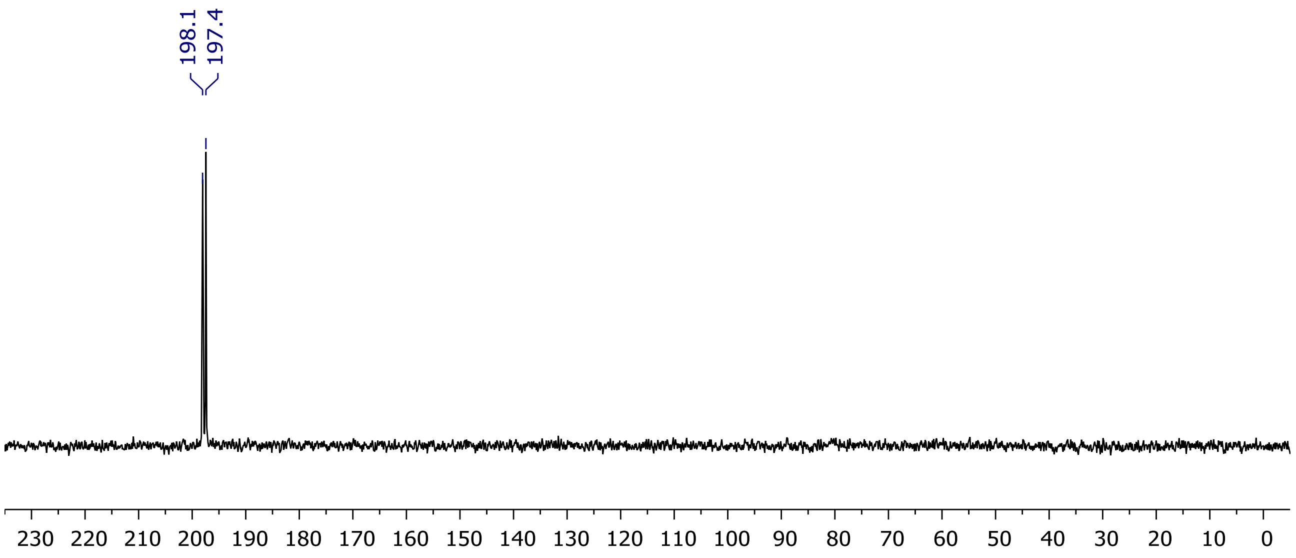

Figure S67. ^{31}P{^{1}H} NMR spectrum recorded after 72 h at room temperature in the dark (162 MHz, CD_2Cl_2).

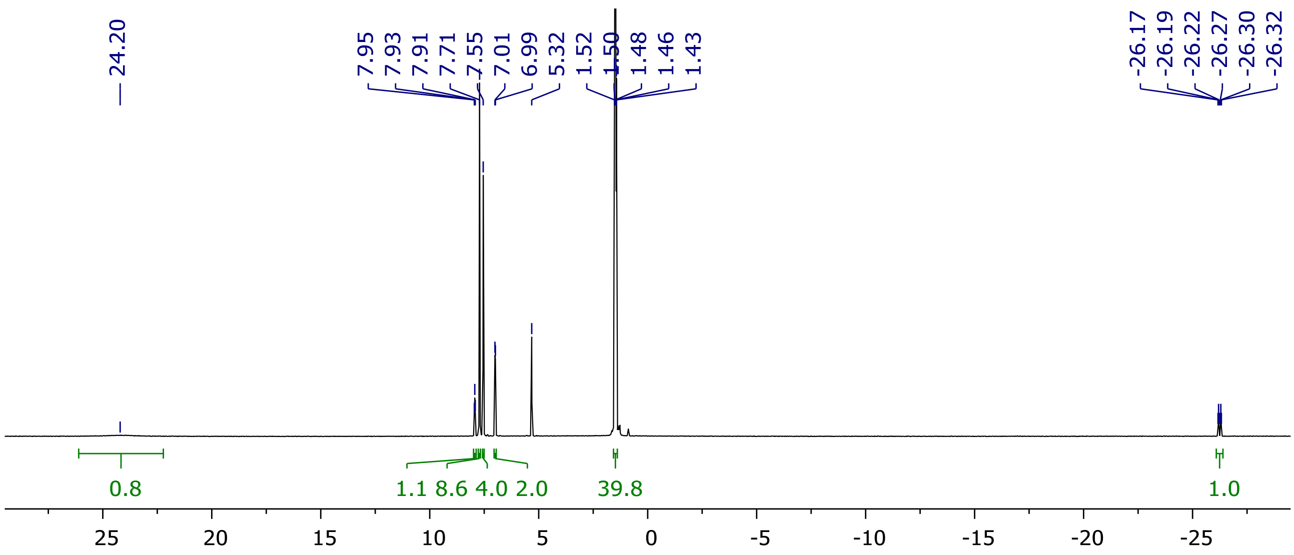

Figure S68. ^{1}H NMR spectrum recorded after 72 h at room temperature exposed to light (400 MHz, CD_2Cl_2).

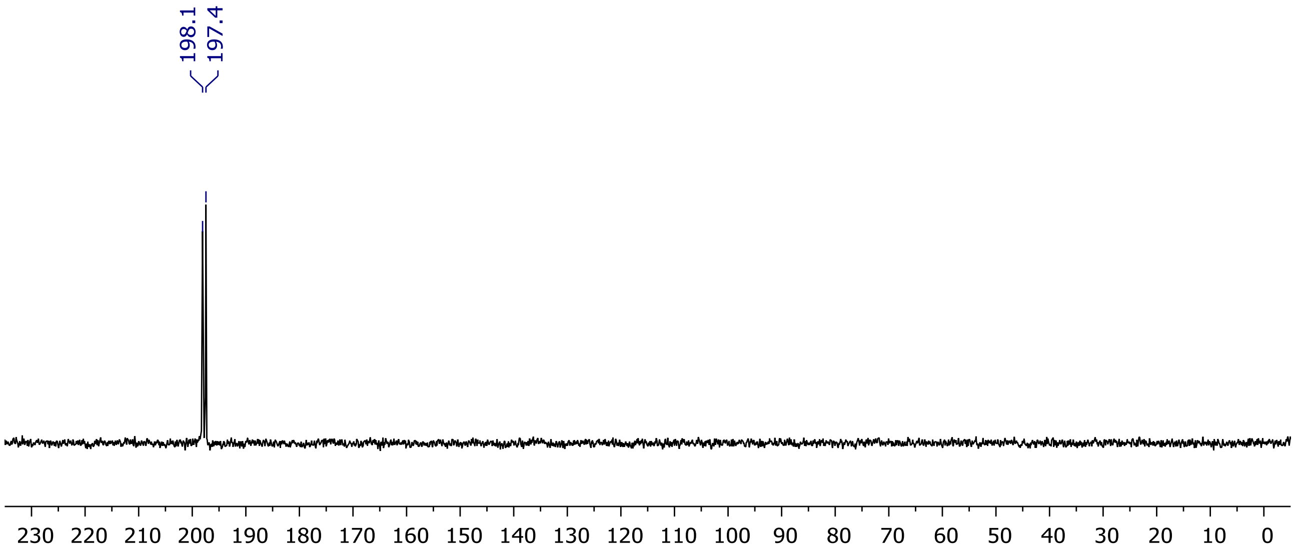

Figure S69. $^{31}P\{^{1}H\}$ NMR spectrum recorded after 72 h at room temperature exposed to light (162 MHz, CD_2Cl_2).

15.2 Reaction with TEMPO in CD_2Cl_2

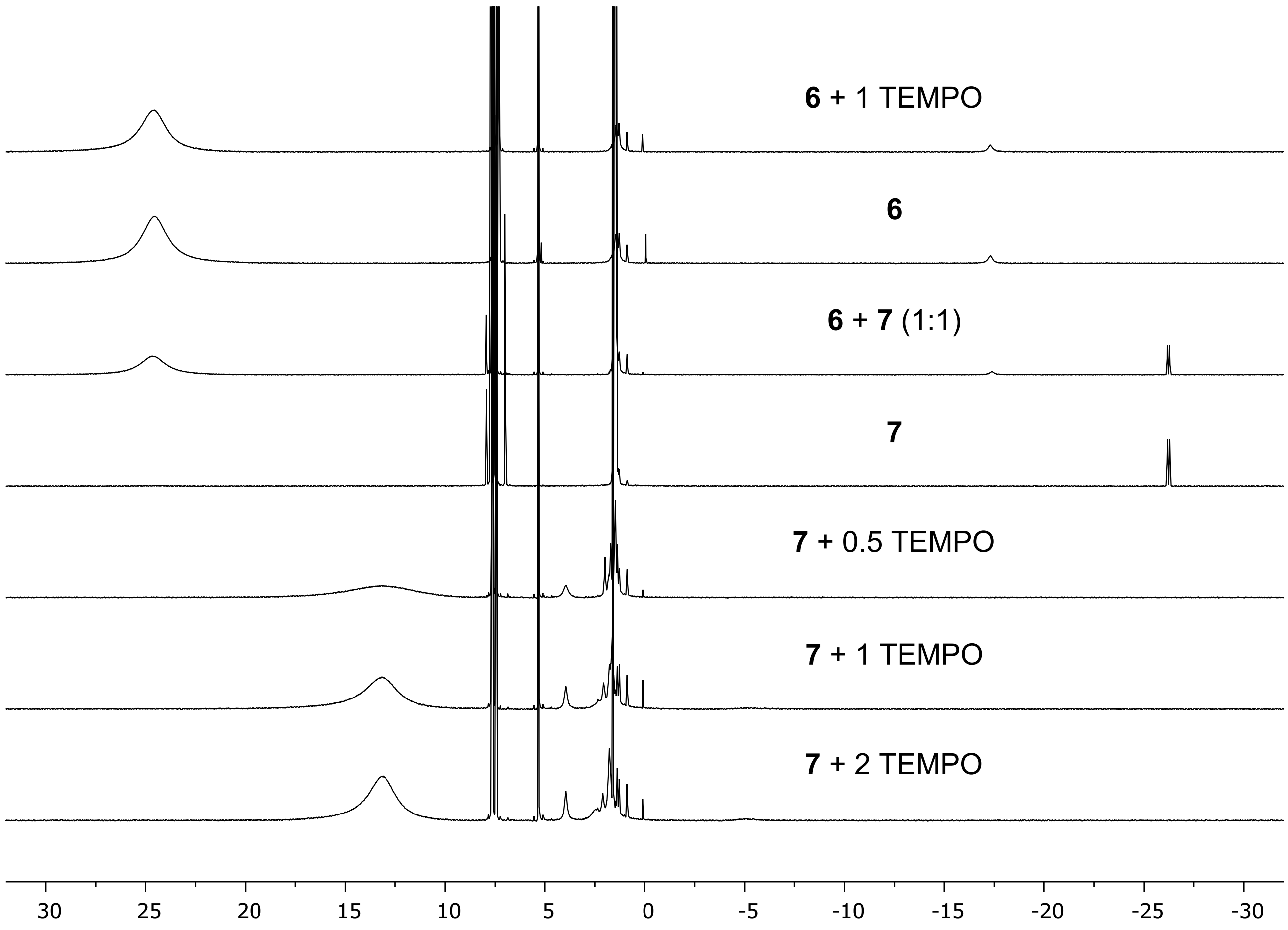

Figure S70. ^1H NMR spectra demonstrating hydrogen atom transfer between **6** and **7** mediated by TEMPO (400 MHz, CD_2Cl_2).

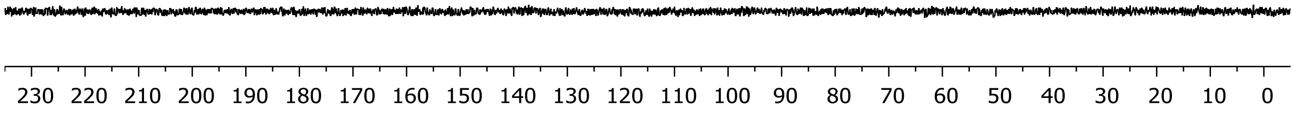

Figure S71. $^{31}P\{^1H\}$ NMR spectrum recorded for the reaction between **7** and 1.0 equivalent of TEMPO (162 MHz, CD_2Cl_2).
